# Microbial Consortium Associated with the Antarctic Marine Ciliate *Euplotes focardii*: An Investigation from Genomic Sequences

**DOI:** 10.1007/s00248-015-0568-9

**Published:** 2015-02-24

**Authors:** Sandra Pucciarelli, Raghul Rajan Devaraj, Alessio Mancini, Patrizia Ballarini, Michele Castelli, Martina Schrallhammer, Giulio Petroni, Cristina Miceli

**Affiliations:** 1 School of Biosciences and Veterinary Medicine, University of Camerino, Camerino, 62032 Italy; 2 Department of Biology, University of Pisa, Pisa, 56126 Italy; 3 Institute of Biology II, University of Freiburg, Freiburg, 79104 Germany

**Keywords:** Psychrophile, *Proteobacteria*, *Bacteroidetes*, Catabolism, Gene transfer, Antifreeze proteins

## Abstract

We report the characterization of the bacterial consortium associated to *Euplotes focardii*, a strictly psychrophilic marine ciliate that was maintained in laboratory cultures at 4 °C after its first isolation from Terra Nova Bay, in Antarctica. By Illumina genome analyser, we obtained 11,179 contigs of potential prokaryotic origin and classified them according to the NCBI’s prokaryotic attributes table. The majority of these sequences correspond to either *Bacteroidetes* (16 %) or *Proteobacteria* (78 %). The latter were dominated by gamma- (39 %, including sequences related to the pathogenic genus *Francisella*), and alpha-proteobacterial (30 %) sequences. Analysis of the Pfam domain family and Gene Ontology term variation revealed that the most frequent terms that appear unique to this consortium correspond to proteins involved in “transmembrane transporter activity” and “oxidoreductase activity”. Furthermore, we identified genes that encode for enzymes involved in the catabolism of complex substance for energy reserves. We also characterized members of the transposase and integrase superfamilies, whose role in bacterial evolution is well documented, as well as putative antifreeze proteins. Antibiotic treatments of *E. focardii* cultures delayed the cell division of the ciliate. To conclude, our results indicate that this consortium is largely represented by bacteria derived from the original Antarctic sample and may contribute to the survival of *E. focardii* in laboratory condition. Furthermore, our results suggest that these bacteria may have a more general role in *E. focardii* survival in its natural cold and oxidative environment.

**Electronic supplementary material:**

The online version of this article (doi:10.1007/s00248-015-0568-9) contains supplementary material, which is available to authorized users.

## Introduction

The Antarctic polar region represents a natural laboratory for evolutionary ecology studies. The cold but thermally stable seawaters of the Southern Ocean offer a large source of microbial ecosystems useful for exploring speciation and evolution with limited phenomena of gene flow from outside biota. This is due to the geographical isolation of the Antarctic continent since its separation from Gondwanaland and the formation of the Polar Front 25 mya ago [1]. Ciliates (Ciliophora, Alveolata) represent a large fraction of eukaryotic microbes inhabiting the Antarctic coastal seawaters [2]. They are unicellular eukaryotes occupying ecological niches in nearly all environments, where they play an essential role in the “microbial loop” [3]. In challenging and harsh ecosystems, ciliates may host different bacterial species [4–6]. For instance, ciliates living in an anaerobic habitat recurrently harbor hydrogen-consuming-bacteria that can use hydrogen as their main and vital substrate [7, 8]. Whether these bacteria have a key role in environmental adaptation and/or in enhancing the fitness of the host is not well established [7]. Even though some associations appear to be obligatory for both ciliates and bacteria, others may be ecologically advantageous but not vital for the host organism [9, 10].

Higher eukaryotic organisms including humans are intimately associated with complex communities of microbes, the so-called microbiome, which are essential for development, health, and interactions with the environment [11]. For example, the marine oligochaete *Olavius algarvensis*, a worm lacking mouth, gut, and nephridia, is associated with a microbial consortium that provides the host with multiple sources of nutrition and energy as it shuttles between the upper oxic and lower anoxic coastal sediments where it lives [12]. According to our best knowledge, bacterial consortia associated with ciliates have never been studied in detail. Among unicellular eukaryotes, such kind of studies have been performed in order to characterize the bacterial community associated to cultures of dinoflagellates and to determine their possible involvement of such consortia in the production of dinoflagellate toxins [13, 14]. It has been shown that clonal cultures of *Ostreopsis lenticularis* show a peak of toxicity during the stationary growth phase, correlated with a significant increase in bacteria directly associated with these cells. This suggests a role in toxin production, at least in the laboratory culture [14].

Here, we report the characterization of the bacterial consortium associated to a laboratory culture of the marine ciliate *Euplotes focardii* [15]. This ciliate is a free-swimming protozoan endemic of the oligothrophic coastal sediments of Terra Nova Bay, in Antarctica. It has been maintained in the laboratory for more than 20 years after its first isolation. Its temperature optimum is about 4–5 °C with a decline at 8–10 °C. *E. focardii* does not show a long survival if exposed to temperatures over 10 °C [16, 17]. Therefore, it is classified as an obligate psychrophilic stenothermal organism [18–24]. By Illumina genome analyser, we obtained 11,179 contigs that were considered to be of prokaryotic origin. Most of these contigs matched with orthologous sequences from *Proteobacteria* and *Bacteroidetes*. The majority of the bacterial sequences were attributed to physiological adaptations to cold, oxidative environments and to facilitate horizontal gene transfer. The characterization of the bacterial consortium in *E. focardii* contributes to understand how different organisms cooperate for environmental adaptation.

## Materials and Methods

### Cell Strains and Growth Conditions

Cell cultures of the *E. focardii* strain TN1 and TN2 [15] were used. They were isolated from coastal sediment and seawater samples collected in Terra Nova Bay (Antarctica) at the beginning of 1988 and 1989 (Ross Sea: temperature, −1.8 °C; salinity, 35‰; pH, 8.1–8.2). These cultures were grown at 4 °C and fed with the green alga *Dunaliella tertiolecta*.

### DNA Isolation and Sequencing

DNA was purified from *Euplotes focardii* cultures as previously described [25]. Sequencing was performed by Illumina paired-end technology (a total of 43,588,788 reads covering 4,402,467,588 bp, with an average read length of 100 bp), in collaboration with Dr. Vadim Gladishev’s research group (Brigham and Women’s Hospital and Harvard Medical School, Boston). The sequences were assembled using Newbler.

### Preparation of Microbial Dataset and Data Analysis

To identify bacterial genomic sequences, all contigs were compared with bacterial genomes available from NCBI (ftp://ftp.ncbi.nlm.nih.gov/genomes/Bacteria/). For the identification of significant similarities, the e-value was set to 1e-1. Thus, identified sequences were subsequently compared with the nucleotide database of NCBI (ftp://ftp.ncbi.nlm.nih.gov/blast/db/). Obtained BLASTn results were uploaded in Linux version of MEGAN5 (Metagenome Analyzer) and binned [26]. Eukaryotic sequences identified after binning were removed from the dataset. The remaining sequences were considered to be potentially of bacterial origin and classified according to the NCBI’s prokaryotic attributes table (derived from:http://www.ncbi.nlm.nih.gov/genomes/lproks.cgi). In total, the genome assembly of the bacterial consortium consisted of 11,179 contigs. The dataset was annotated and clustered using the CAMERA 2.0 (Community Cyberinfrastructure for Advanced Microbial Ecology Research & Analysis) [27] workflow RAMMCAP (Rapid Analysis of Multiple metagenomes with a Clustering and Annotation Pipeline) for the identification of tRNA, rRNA, and ORFs. The ORFs were annotated against Pfam (release 26.0) [28], TIGRFAM 11.0 [29], and COG databases version 4.2.3. [30]. Hidden Markov model (HMM) based rRNA finding option was selected to identify rRNA genes [31]. Gene ontology and Pfam domain families comparison among the largely available groups was done using CoMet [32].

### Phylogenetic Analysis

The 70 bacterial contigs containing (partial) 16S rDNA sequences were mostly non-overlapping and rather short, thus prohibiting a full phylogenetic analysis. Therefore, an alternative approach was applied. A set of almost full-length 16S rDNA reference sequences was selected from the NCBI nucleotide database, starting from the respective BLASTn results. One hundred twelve reference sequences were selected in this way and, together with the above mentioned contigs, aligned with more than 450,000 16S rDNA sequences (from the SILVA 111 database release 2012 according to [33]) using the ARB software package 5.2 [34]. The aligned reference sequences were trimmed adjusting to the length of the shortest one at both ends. Moreover, due to the broad phylogenetic spectrum of reference sequences, more variable positions (i.e., columns comprising a single gap) were removed from the alignment. This final alignment comprised 1043 columns and was used to build the “scaffold tree” containing the selected 112 reference sequences. Phylogenetic reconstruction of the “scaffold tree” was performed using the maximum likelihood program PhyML [35] included in the ARB package [34]. The analysis was performed on the above mentioned final alignment applying the GTR + I + G model. The selection of this model was confirmed by jModelTest2 (version 2.1.4) [36].

Subsequently, all except six short contigs were added to the “scaffold tree” with the Quick-add parsimony function of ARB, with the default ARB settings for bacterial sequences. For this purpose, considering that the reference sequences present in the “scaffold tree” were selected because of their high similarities to the newly characterized contigs, all nucleotide positions of the alignment, including highly variable ones, were used. Only 16S rDNA-flanking regions, if present in the contig, were removed prior to the analysis.

### Identification of *Francisella* Homologues

All available genome sequences of *Francisella* species were retrieved from NCBI site, and blast was performed with the microbial dataset. Reciprocal blast was done, and all the blast hits were collected. BLASTn and BLASTx were performed on NCBI database using default parameters to remove false positives and for sequence annotation.

### Antifreeze Protein Prediction

Antifreeze protein prediction was done by AFP-Pred [37] and iAFP [38].

### Antibiotic Treatments and Growth Rate Estimation of *E. focardii* Cells

To determine the suitable antibiotic concentration to remove the bacterial consortium associated to the ciliate, *E. focardii* cells were treated for 7 days with 1000 U/ml (1 U equivalent to 0.6 μg penicillin-G) of penicillin-G, and 1000 U/ml of streptomycin, or both, in sea water (salinity 33 ‰). The treated *E. focardii* cells showed normal capacity of dividing and moving. A control for each test was conducted with *E. focardii* cells not treated with antibiotics.

For the growth rate estimation of bacteria-free *E. focardii* cells, ten cells were collected from mass cultures, transferred into a 30-mm diameter Petri dish (Sterilin), fed with *Dunaliella tertiolecta*, and treated with both penicillin-G (1000 U/ml) and streptomycin (1000 U/ml). After 15 days, cells were transferred to the normal antibiotic-free culture medium and again fed with *D. tertiolecta*. The same number of cells was taken as control, fed in the same way, and maintained in an antibiotic-free medium. The number of cells was counted every 24 h for 20 day from the beginning of the experiment. Data and statistical analyses were performed using Excel.

## Results

### Characterization of the Microbial Diversity Present in the Consortium

**Fig. 1:**
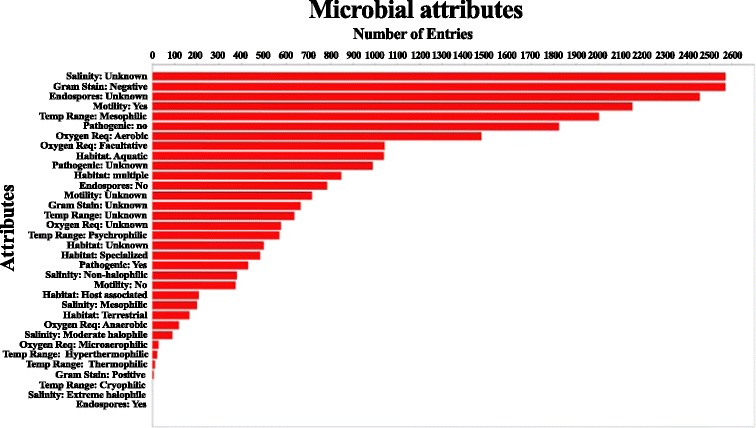
Microbial attributes of the *E. focardii* bacterial consortium dataset

**Fig. 2:**
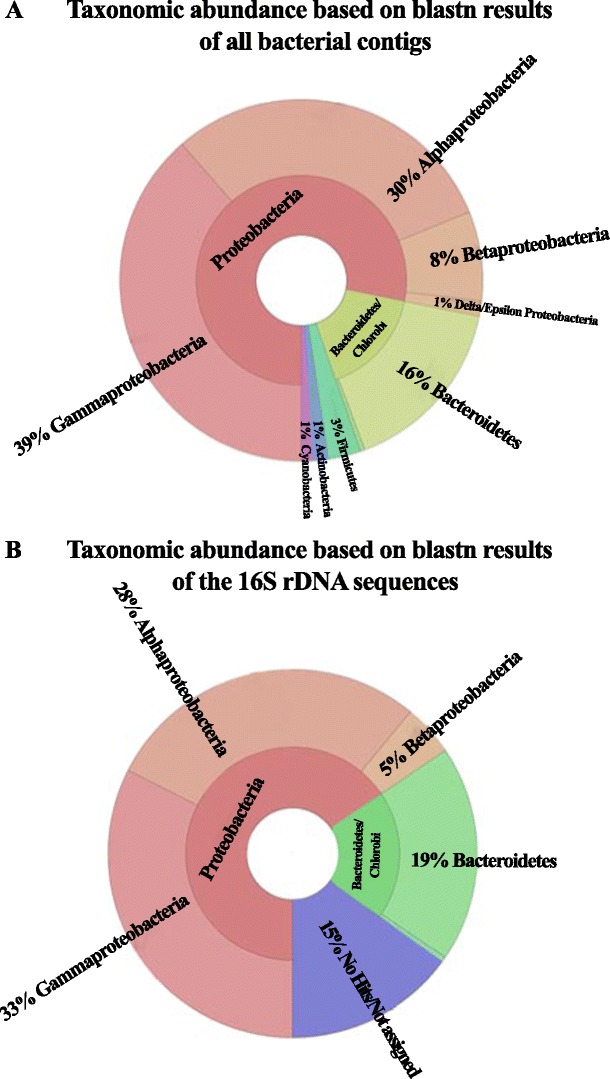
Pie charts of the taxonomic abundance of the bacterial consortium based on BLASTn results of all the contigs (**a**) and of the 16S rDNA sequences (**b**)

By Illumina genome analyser, we obtained 11,179 contigs lacking at their ends the 5′-CCCCAAAA-3′/3′-GGGGTTTT-5′ telomeric repeats characteristic of the *Euplotes* nanochromosomes. These contigs ranged in size from 100 to 25,584 bp and did not correspond to any eukaryotic sequences after binning. Therefore, these contigs were considered to be of potential prokaryotic origin (termed here microbial dataset). Comparison to NCBI’s prokaryotic attributes table showed that about 2600 of the entries were attributed to the categories “marine environment” and “Gram-negative bacteria” (Fig. 1). More than 2100 entries were associated to “motile bacteria”, and nearly 600 entries were attributed to “psychrophilic microorganisms”. *Proteobacteria* (78 %) was the most abundant phylum of bacteria, represented mainly by *Gammaproteobacteria* (39 % of the total bacteria), followed by *Alphaproteobacteria* (30 %), and *Betaproteobacteria* (8 %) classes (Fig. 2a). Other sequences were classified as follows: *Bacteroidetes* (16 %) *Firmicutes* (2.64 %), *Actinobacteria* (1.22 %), *Cyanobacteria* (1.00 %), *Chlamydiae*/*Verrucomicrobia* (0.37 %), *Planctomycetes* (0.22 %), *Spirochaetes* (0.19 %), *Acidobacteria* (0.13 %), and *Fusobacteria* (0.09 %), whereas 0.45 % of the contigs affiliated to sequences from *Archaea* (due to the low number of *Archea* sequences the consortium will be referred as bacterial consortium). A similar distribution of the most represented classes was obtained by analyzing the 70 contigs corresponding to partial sequences of the 16S rDNA gene (Fig. 2b).

### Phylogenetic Analysis of the Bacterial Consortium

**Fig. 3:**
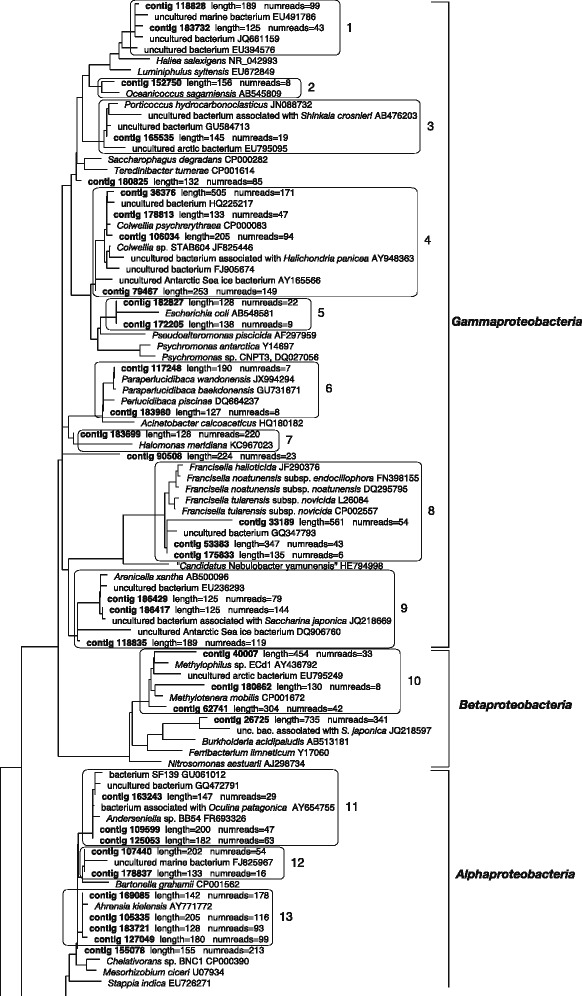

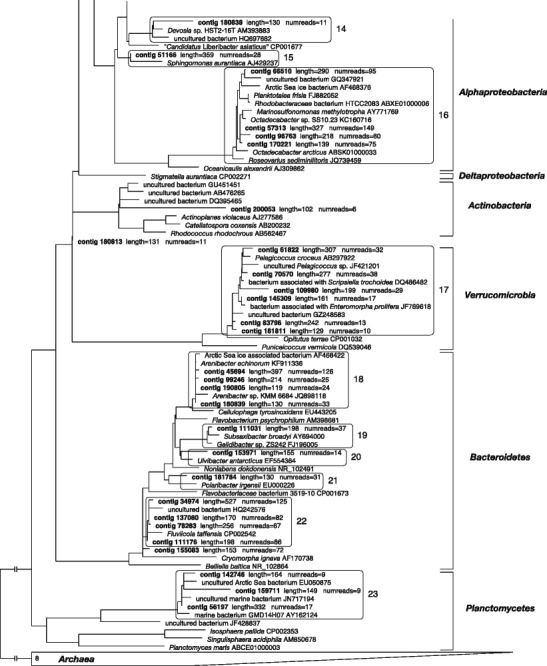
Maximum likelihood phylogenetic tree based on 16S rDNA reference sequences (available in the ARB database) with the subsequent addition of 64 contigs of 16S rDNA retrieved from the consortium (listed in Fig. S1. Due to graphic requirements, the branches between ingroup and outgroup are not shown entirely, the full tree is available in the Supplementary Material). Phylogenetically-related contigs have been enclosed, for clarity, in a box together with their closest relatives. A total of 23 such groups were identified, nine among *Gammaproteobacteria*, one among *Betaproteobacteria*, six among *Alphaproteobacteria*, one among *Verrucomicrobia*, five among *Bacteroidetes*, and one among *Planctomycetes*

Figure 3 shows the phylogenetic tree based on the 16S rDNA sequences selected from the NCBI nucleotide database with the added consortium contigs. At the higher taxonomic levels, the tree reflected almost perfectly the results of the BLASTn analysis. Additionally, in most cases, it was possible to recognize the affiliation of the contigs to the family or even genus rank. Several contigs cluster with bacterial sequences derived from habitats which share similarities to the Antarctic environment where the original sample of *E. focardii* was collected, such as the Alaskian Byron glacier [39], Arctic and Antarctic coastal seawaters, or other cold environments (e.g., *Colwellia psychrerythraea*, *Ahrensia kielensis*, *Octadecabacter arcticus*, etc.) [40–42]. Furthermore, we detected phylogenetic relationships to sequences retrieved from bacteria living in tight association with eukaryotic organisms (e.g., *Arenibacter echinorum*, originally isolated from the sea urchin *Strongylocentrotus intermedius*, and *Porticoccus hydrocarbonoclasticus*, found associated with the dinoflagellate *Lingulodinium polyedrum*) [43, 44], and, notably, even to genera that encompass endosymbionts of ciliates (i.e., *Francisella* and *Devosia*) [10, 45]. We additionally observed that some contigs are affiliated with organisms characterized by special metabolic repertoires (e.g. *Marinosulfonomonas methylotropha*, *Methylotenera mobilis* or *P. hydrocarbonoclasticus*) [43, 46] or possess genomes rich in transposases and other mobile genetic elements (genus *Octadecabacter*) [47].

### Characterization of Enzymes Involved in Recycling of Organic Matter and/or Biodegradation

The phylogenetic analysis showed that some members of the bacterial consortium of *E. focardii* are closely related to species involved in recycling organic material or in biodegradation. In fact, it is already documented that strains of *Flavobacteria* isolated from Antarctic marine waters have an important role in recycling organic material present in the sea floor [48]. Furthermore, *Colwellia psychrerythraea* is able to produce polyhydroxyalkanoate (PHA) compounds, a family of polyesters that serve as intracellular carbon and energy reserves, some of which have been linked to pressure adaptation [49]. To identify bacterial sequences of the *Euplotes* consortium potentially involved in these metabolisms, the microbial dataset was explored by tBLASTn using as query a selection of corresponding key enzymes (Table S1). The results (Table S2) revealed the presence of both polyhydroxyalkanoate synthase and depolymerase, suggesting that also the members of the *E. focardii* consortium are able to produce PHA compounds and use them as energy reserves. Contigs encoding enzymes involved in the degradation of aromatic compounds, as catechol 2,3 dioxygenase and naphthalene-degradation, were also detected. We also identified contigs encoding cyanophycin synthases (cyanophycin-like compounds serve as nitrogen reserves) (Table S2). Furthermore, members of these bacterial consortium possess enzymes that enable the utilization of aromatic compounds for growth, such as Anthranilate 1,2-dioxygenase, and of F420-dependent NADP reductase [50]. Coenzyme F420 was first found in methanogens where it is important in methanogenesis [51]. The bacterial organism *Rhodococcus* uses the coenzyme F420 for polynitroaromatic compound degradation. These results suggest that members of the bacterial consortium are able to carry out aromatic compound metabolism, an important ability for utilizing alternative carbon sources.

### Gene Ontology Annotation of the Bacterial Contigs

**Fig. 4:**
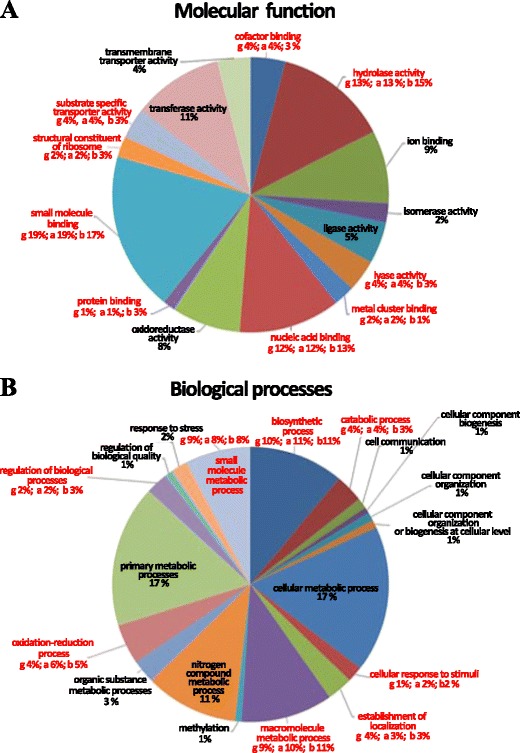
Gene Ontology (GO) annotation of the dataset from the three most represented bacterial groups (*Gammaproteobacteria*, *Alphaproteobacteria*, and *Bacteroidetes*) for molecular function (**a**) and biological processes (**b**). In each panel are reported rounded off percentages of the represented terms. In cases in which rounded off percentage values were identical for the three bacterial groups, only one value is reported in black (e.g., ion binding, 9 %); in cases in which rounded off percentage values were differing in the three group, the different values are reported in red and they, respectively, refer to *Gammaproteobacteria* (g), *Alphaproteobacteria* (a), and *Bacteroidetes* (b)

Gene Ontology (GO) annotation was performed for contigs belonging to the three dominant taxa, i.e.,*Gammaproteobacteria* (g), *Alphaproteobacteria* (a), and *Bacteroidetes* (b) (Fig. 4). A gene description and GO classification based on the “best hit” from the blastx search was assigned to each contig. For simplicity, only the categories of the three bacterial groups whose rounded off values were 1 % or higher were reported (Fig. 4). In the three groups, the most frequent Molecular Function categories were “small molecule binding” (19 % (g), 19 % (a), and 17 % (b), respectively), hydrolase activity (13 % (g), 13 % (a), and 15 % (b)), nucleic acid binding (12 % (g), 12 % (a), 13 % (b)), transferase activity (11 %), ion binding (9 %), and oxidoreductase activity (8 %). For the “Biological Process” categories, the largest number of contigs corresponded to molecules involved in cellular metabolic processes (17 %), primary metabolic processes (17 %), and nitrogen metabolism (11 %) (Fig. 4b).

### Comparative Functional Profiling of Bacterial Contigs: Variation of Pfam and GO Terms

**Fig. 5:**
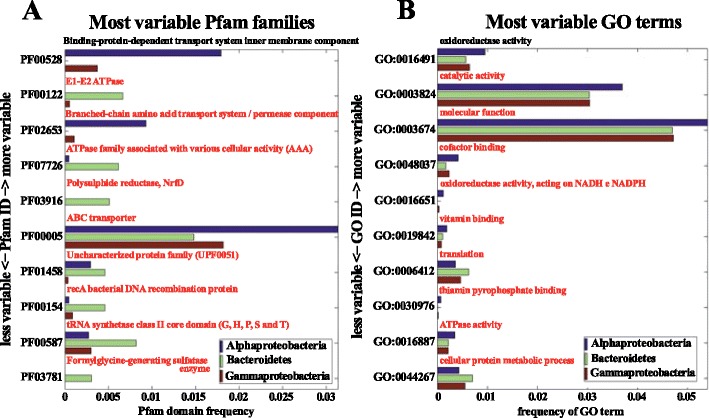
Variable Pfam families and GO terms among the three most represented bacterial groups (*Gammaproteobacteria*, *Alphaproteobacteria*, and *Bacteroidetes*) done using CoMet [32]. The bar charts show the Pfam domain (**a**) and GO term categories (**b**) with the highest frequencies in the consortium (*x*-axis values), and the largest terms variation with respect to precomputed profiles obtained from microbial metagenomes in the CoMet server (*y*-axis values)

We also estimated the most represented functional categories in the three dominant bacterial groups (*Gammaproteobacteria*, *Alphaproteobacteria*, and *Bacteroidetes*) in the consortium with respect to the metagenome dataset available in the CoMet server [32]. The bar charts in Fig. 5a, b show the Pfam domain frequencies and GO term categories, respectively, with the highest frequencies in the consortium (*x*-axis values) and the largest terms variation compared to precomputed profiles obtained from microbial metagenomes in the CoMet server (*y*-axis values). This comparison is crucial for an understanding of community specific properties that are possibly linked with particular environmental factors. From this analysis, it resulted that the Pfam domain family with the largest variation with respect to the other microbial metagenomes is represented by proteins involved in transmembrane transport (Fig. 5a). The GO-terms analysis shows proteins involved in “oxidoreductase activity” at the highest value of variation, thus suggesting a high metabolic potential of the consortium to survive in an oxygen-rich environment, followed by the “catalytic activity” and “molecular function” terms (Fig. 5b).

### Identification of Sequences Encoding Ice-Binding Proteins

We focused on bacterial sequences encoding ice-binding proteins (IBPs) or antifreeze (AFPs) as these are considered the evolutionary key molecules for cold-adaptation. We found two sequences organized in tandem in the same contig: one is 57.43 % identical to the antifreeze protein from the *Stigmatella aurantiaca* strain DW4/3-1, which was isolated from the glacier of the Lower Victoria valley (Antarctica) [52] and the second is 52 % identical to the IBP from the *Flavobacteriaceae* bacterium strain 3519-10, which was isolated from the glacial ice of the Lake Vostok [53]. These two sequences have been previously described in detail in [54]. Furthermore, the complete genomic dataset was explored by performing a tBLASTn using AFP sequences from the diatom *Fragilariopsis cylindrus* as queries. We found four contigs (Table S3) that are similar to the bacterial type I AFP from *Rhodobacteraceae* bacterium HTCC2083 [55] and one sequence sharing 45 % similarity to type II AFP from the catadromous fish *Lates calcarifer* [56]. Finally, we performed a prediction of putative AFP using AFP-Pred and iAFP bioinformatic tool as described above. Following this approach, we found 2755 or 600 (using AFP-Pred respectively iAFP) contigs that potentially encode AFPs in *Gammaproteobacteria*, 2251or 500 (by AFP-Pred respectively iAFP) in *Alphaproteobacteria*, and 942 or 200 (by AFP-Pred respectively iAFP) in *Betaproteobacteria*. These sequences may represent new AFPs whose AFP activity waits for verification.

### Identification of Sequences Involved in Horizontal Gene Transfer

Gene transfer between cells is a powerful mechanism of genome evolution [57]. One interesting mode of gene transfer is carried out by gene transfer agents (GTAs), phage-like elements that package small segments of the genome of a GTA-producing cell and transmit these genes throughout the environment [57]. These genes have been characterized principally in *Alphaproteobacteria* [57].

In the *E. focardii* bacterial consortium, we identified a putative GTA encoding gene with a similarity of 89 % to a putative GTA protein from the *Alphaproteobacterium Ahrensia kielensis* [58]. Furthermore, we also found contigs that correspond to members of the transposase and integrase superfamilies (see Table S4). Transposable genetic elements (TEs) and transposition may play a profoundly generative role in genome evolution and adaptation (see Discussion).

### Identification of *Francisella* Sequences

In addition to 16S rDNA sequences, we identified further 193 contigs (1.70 % of all non-16S rDNA containing bacterial contigs) that showed a high similarity to sequences from representatives of the genus *Francisella* [59]. These species are*: F. cantonensis* (1 contig), *F. novicida* (40 contigs), *F. tularensis* (42 contigs), *F. noatunensis* (30 contigs), *F. philomiragia* (41 contigs), *Francisella* sp. GP-2009 (2 contigs), and *Francisella* sp.TX077308 (37 contigs) (see table S5). Twenty-two contigs correspond to 23S rDNAs or ribosomal proteins. By gene annotation, the following categories were identified: stress response, transport, protective mechanisms against viruses, enzymes involved in substrate degradation, catabolism and metabolism, DNA mobilization and repair, and cell cycle (see Table S5). We also identified a transposase and DNA mismatch repair enzyme with ATPase activity showing high similarity to that of the human pathogen *F. tularensis* [60].

### Contribution of the Bacterial Consortium to *E. focardii* Growth Under Laboratory Conditions

**Fig. 6:**
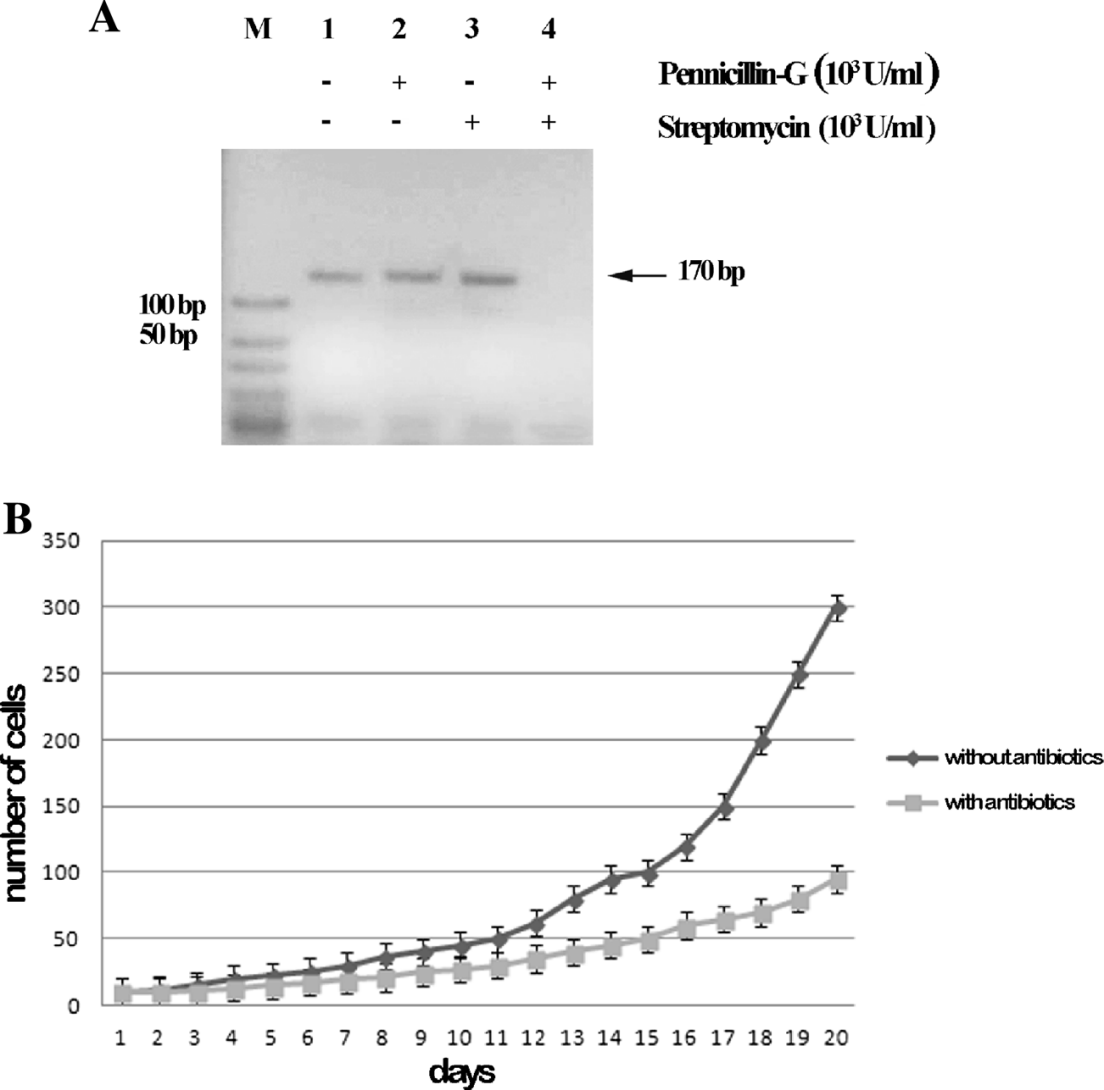
Analysis of *E. focardii* cells previously exposed to antibiotic treatments. **a** Agarose gel of PCR reaction performed on purified genomic DNA from *E. focardii* cells without antibiotics (lane *1*) and after treatment with 10^3^ U/ml of penicillin-G (lane *2*), 10^3^ U/ml of streptomycin (lane *3*), and with a mix containing 10^3^ U/ml of both penicillin-G and streptomycin (lane *4*), using as primers a mix of oligonucleotides that amplify a fragment of 170 bp of the 16S rDNA sequences. **b** Growth rate of untreated *E. focardii* cells associated to their long lasting microbial consortium and *E. focardii* cells preliminary treated with a mix containing 10^3^ U/ml of both penicillin-G and streptomycin for 15 days to deplete the microbial consortium associated to the ciliate. The cells were counted for 20 days

In order to test the possible contribution of the bacterial consortium to *E. focardii* cold-adaptation, the viability and proliferation of *E. focardii* cells under laboratory conditions after treatment of the cultures with the antibiotics penicillin and/or streptomycin was analyzed. The genomic DNA from *E. focardii* cells was extracted after the antibiotic treatment, and PCR was performed using a mix of oligonucleotides amplifying a conserved fragment of the 16S rDNA sequence (170 bp). No PCR product was obtained in the sample from *E. focardii* cells treated with both antibiotics (Fig. 6a), confirming that only the treatment with both penicillin-G and streptomycin efficiently remove the bacterial consortium from *E. focardii* cultures. Subsequently, we analysed the growth rate of *E. focardii* cells after the combined treatment with the two mentioned antibiotics over a period of 20 days. *E. focardii* cells showed a significant lower growth rate (*p* value = 0.0224) with respect to the control (i.e., not previously treated with the two antibiotics; Fig. 6b). This result suggests that the bacterial consortium is not essential for the survival of *E. focardii* cells, but its elimination reduces cell proliferation capabilities.

## Discussion

In this study, we report the characterization of the bacterial consortium associated to a long-term stabilized culture of the psychrophilic ciliate *E. focardii*, a species endemic of the Antarctic coastal seawaters [15]. To our best knowledge, this is the first bacterial consortium associated to a stabilized ciliate culture that has been characterized so far. Indeed, in the last years, several studies on ciliate-bacteria associations focused on the characterization of bacterial symbionts of ciliates [6, 61–64], but in no cases, a detailed analysis of the microbial consortium associated to cultured cells were performed. In the present study, a metagenome-like approach followed by a bioinformatic analysis allowed to characterize the bacterial community presently associated to an *E. focardii* strain culture maintained in laboratory for more than 20 years. Unlike the signature genes approach, that in most of the cases is based on the analysis of the 16S ribosomal RNA genes, this metagenomic approach allowed us to obtain a much wider genetic information of the microbial community, and was used to generate also a description of the functional potential of the investigated bacterial consortium. This consortium may have an important role in defining the ecological potential of the ciliates in terms of growth capabilities and adaptation to cold environments. The binning of the bacterial dataset revealed that most of the contigs of this microbial consortium correspond to orthologous sequences from *Proteobacteria* (66 %) and *Bacteroidetes* (19 %). Intriguingly, the pattern of taxonomical distribution found in this long lasting *E. focardii* laboratory strain is similar to the one recently reported from an environmental metagenome from Alaskan glacier [38]. More than 600 of the characterized sequences showed psychrophilic features suggesting that a significant fraction of the bacterial community associated to *E. focardii* culture may still be representative of the original bacterial community derived from the original Antarctic sample. This possibility is also supported by phylogenetic analysis of retrieved 16S rDNA sequences that, in many cases, showed to be related to bacterial strains or uncultured organisms derived from an Arctic or Antarctic environment. *Escherichia coli* related contigs in the phylogenetic tree may represent a low contamination either in the consortium or that occurred during sample preparation (e.g. DNA extraction, library construction, etc.), or may derive from representatives of *Enterobacteriaceae* actually present in the consortium, but not necessarily from *E. coli*. The 16S rDNA is not commonly used as exclusive phylogenetic marker for the identification of *E. coli* or other members of the family *Enterobacteriaceae* because they all share rather high 16S rDNA similarities. Generally, other genes such as gyrB and rpoA are used for taxonomy in combination with 16S rDNA in this family.

The phylogenetic analysis also enables the identification of the bacterial genera that compose the consortium. Some of these contigs appear closely related to the strictly psychrophilic *Gammaproteobacteria Colwellia psychrerythraea* 34H strain, isolated from Antarctic sea sediments [41]. Other contigs appear closely related to representatives of the genus *Francisella*, which are facultative intracellular microbes causing severe disease in a broad range of animals. In particular, *F. tularensis* is the causative organism of tularemia and a putative warfare agent, whereas *F. noatunensis* is an emerging fish pathogen causing significant losses in wild and farmed fish [64]. Protists have been suspected to serve as a disguised vector of *Francisella*, and co-culturing attempts demonstrated that some species are able to survive and multiply within protozoan cells [65, 66]. A representative of *Francisella* has been previously characterized as endosymbiont in another *Euplotes* species derived from a temperate environment [6]. To our best knowledge, this is the first report of the characterization of *Francisella* sequences associated to an organism derived from Antarctic environment. Our results can contribute to uncover new environmental reservoirs of old diseases.

Most of the *Alphaproteobacteria* sequences of the consortium belong to the order *Rhodobacterales*. Marine *Rhodobacterales* are a widespread, abundant, and metabolically versatile bacterial group in the world’s oceans. They also show a nearly universal conservation of the genes for production of GTAs, virus-like particles that mediate genetic exchange between cells [67]. A GTA encoding gene is present in the *E. focardii* bacterial consortium, as well as members of the transposase and integrase superfamilies. The significance of horizontal gene transfer in bacterial evolution and creation of “novel” catabolic pathways for bacterial adaptation is now well established. Fully sequenced genomes revealed that a substantial fraction of ORFs have been horizontally transferred [68, 69], and many of these acquisitions are thought to have driven adaptation to new ecological niches [70].

Transposable genetic elements (TEs) and transposition may play a profoundly generative role in genome evolution [71] and in the responsive capacity of organisms in the face of environmental challenges. TEs are likely to play a relevant role in adaptation because of their ability to generate mutations of great variety and magnitude, and their capacity to be responsive and susceptible to environmental changes [72–75].

Although our study has been performed on a long lasting *E. focardii* laboratory strain, the obtained results strongly suggest that the bacterial consortium presently associated to the ciliate culture largely reflects the original psychrophilic one from the Antarctic environment. Our analysis suggests unique cold adaptation characteristics of the consortium such as the abilities to synthesize and breakdown high molecular weight compounds, as well as carbon metabolism, important in carbon and nutrient cycling in the cold marine environment [48]. Therefore, it appears that the ecological role of the consortium includes catabolism of complex substances for carbon and energy reserves that insures survival when cold environmental conditions may set limitations on uptake of such carbon and nitrogen reserves from other sources [41]. Bacterial intracellular carbon and nitrogen reserves most probably contribute to nutrient provision also to *E. focardii*. In other words, the presence of this consortium associated to *E. focardii* cells may have aided this ciliate to adapt and survive under the highly selective cold conditions of the Antarctic habitat.

Previous papers reported that some *Euplotes* species cannot divide properly and eventually die when the endosymbiontic bacteria are removed by antibiotic treatment [76, 77]. Our analysis shows that antibiotic treated *E. focardii* cells are still able to divide even though antibiotic treatments reduce cell proliferation. This result suggests that the bacterial consortium, beside not essential, is probably helpful to the ciliate in the specific laboratory growing condition and might be useful to the ciliate also in the natural Antarctic environment. This hypothesis is supported not only by the identification of enzymes responsible for carbon and nitrogen storage, but also by the result obtained from the GO annotation in which most of the terms include proteins involved in oxidoreductase activity. It is well known that one of the major problems of marine cold-adapted organisms is to cope with increased O_2_ solubility at low temperatures that determines an increased level of reactive oxygen species (ROS) in their cells. Therefore, increased defenses against oxidative stress most likely constituted an important aspect of evolutionary adaptation of these organisms in their oxygen-rich environment.

An additional contribution of the bacterial consortium to cold-adaptation most likely derives from the synthesis of antifreeze proteins. Among the bacterial sequences, we identified contigs that encode proteins highly similar to the antifreeze proteins identified in other organisms, including *S. aurantiaca* strain DW4/3-1, which was isolated from the glacier of the Antarctic Lower Victoria valley and *Flavobacteriaceae* bacterium strain 3519-10, which was isolated from the glacial ice of the Lake Vostok [54]. Furthermore, by using AFP-Pred and iAFP bioinformatic tools, we predicted 2755 (using AFP-Pred) and 600 (using iAFP) contigs that potentially encode AFPs in *Gammaproteobacteria*, 2251 (by AFP-Pred) and 500 (by iAFP) in *Alphaproteobacteria*, and 942 (by AFP-Pred) and 200 (by iAFP) in *Betaproteobacteria*. These sequences may represent new AFPs which activity must be verified but potentially may contribute to the survival of both bacteria and *E. focardii* cells in the cold.

To conclude, our results support the concept that bacterial consortia associated to other living organisms represent major players in the ecology of species adaptation.

## Electronic supplementary material

Below is the link to the electronic supplementary material.

Fig. S1 (PDF 263 kb)

Fig. S2 (DOCX 20 kb)

Table S1 (DOC 46 kb)

Table S2 (DOC 53 kb)

Table S3 (DOC 45 kb)

Table S4 (DOC 44 kb)

Table S5 (PDF 422 kb)
