# Supplementary figures and images for: Microbial Consortium Associated with the Antarctic Marine Ciliate Euplotes focardii: An Investigation from Genomic Sequences
Source: Microb Ecol. 2015 Feb 24;70(2):484–97. doi: 10.1007/s00248-015-0568-9 (PMC4494151; doi:10.1007/s00248-015-0568-9)

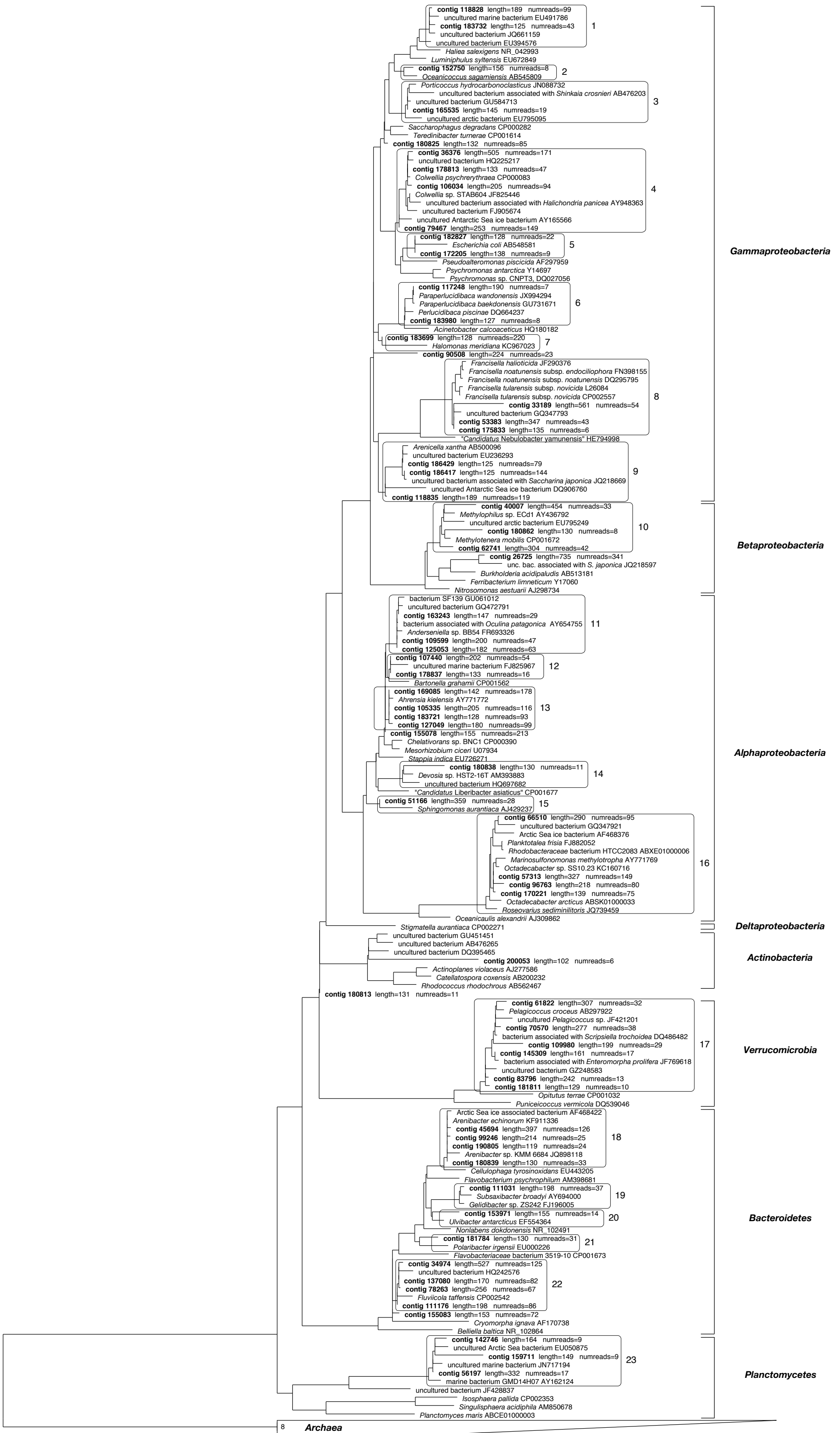

Supplement: Supplementary file 1 — (PDF 263 kb) [file 248_2015_568_MOESM1_ESM.pdf]
