# Supplementary material for: Microbial Consortium Associated with the Antarctic Marine Ciliate Euplotes focardii: An Investigation from Genomic Sequences
Source: Microb Ecol. 2015 Feb 24;70(2):484–97. doi: 10.1007/s00248-015-0568-9 (PMC4494151; doi:10.1007/s00248-015-0568-9)
Supplement: Supplementary file 2 — (DOCX 20 kb) [file 248_2015_568_MOESM2_ESM.docx]

>contig34974
GGGCAATGGAGGCAACTCTGACCCAGCCATGCCGCGTGCAGGAAGAAGGTTCTATGGATCGTAAACTGCTTTTATTTGTGAATAAACTCTCGGACGTGTCCGAGCCTGAATGTAGCAAATGAATAAGCACCGGCTAACTCCGTGCCAGCAGCCGCGGTAATACGGAGGGTGCAAGCGTTATCCGGAATCATTGGGTTTAAAGGGTCCGCAGGCGGACTTATAAGTCAGTGGTGAAATCTCACAGCTTAACTGTGAAACTGCCATTGATACTGTAAGTCTTGAATTCGGTCGAAGTGGGCGGAATATGTCATGTAGCGGTGAAATGCTTAGATATGACATAGAACACCGATAGCGAAGGCAGCTCACTAGGCCTGGATTGACGCTCAGGGACGAAAGCGTGGGGAGCGAACAGGATTAGATACCCTGGTAGTCCACGCCGTAAACTATCAATACTCGTTTTCAGCGATATACAGTTGGAGACTAAGCGAAAGTGATAAGTATTGCACCTGGGGAGTACGATCGCAAGG

>contig45694
CGGCTAACTCCGTGCCAGCAGCCGCGGTAATACGGAGGaTCCGAGCGTTATCCGGAATCATTGGGTTTAAAGGGTCCGTAGGCGGGcCAtTAAGTCAGGGGTGAAAGTCTGCGGCTCAACCGTAGAATTGcCCTTGATACTGGTgGTCTTGAGTTATgGTGAAGTAACTAGAATATGTAGTGTAGCGGTGAAATGCATAGATATTACaTAGAATACCGATTGCGAAGGCAgGTtACTAACATATACTGACGCTGAtGGACGAAAGCGTGGGGAGCGAACAGGATTAGATACCCTGGTAGTCCACGCCGTAAACGATGGATACTAGCTGTCCGGATGCAAATCTGGGCGGCCAAGCGAAAGTGATAAGTATCCCACCTGGGGAGTACGtTCGCAAGaA

>contig137080
TCATCACGGCCCTTACGTCCTGGGCCACACACGTGCTACAATGGTAGATACAGAGGGCAG
CTACCCCGCGAGGGGATGCGAATCTCGAAAGTCTATCTCAGTTCGGATTGGAGTCTGCAA
CTCGACTCTATGAAGCTGGAATCGCTAGTAATCGCGCATCAGCCATGGCG

>contig155083

CGGTGAATACGTTCCCGGGCCTTGTACACACCGCCCGTCAAGCCATGGAAGCtGGGAGTG
CCTGAAGTCGgTAaCCGTAAGGAGCtGCCTAGGGTAAAACTGGTAACTGGGgCTAAGTCG
TAACAAGGTAGCCGTACCGGAAGgtGCGGCTGG

>contig170221

TCTCAGTTCGGATTGGGGTCTGCAACTCGACCCCATGAAGTCGGAATCGCTAGTAATCGCGTAACAGCATGACGCGGTGAATACGTTCCCGGGCCTTGTACACACCGCCCGTCACACCATGGGAGTTGGTCTACCCGaA

>contig105335

GAGACGGCAACGTCGAGCTAATCTCCAAAAACCATCTCAGTTCGGATTGGGGTCTGCAACTCGACCCCATGAAGTTGGAATCGCTAGTAATCGTGGATCAGCATGCCACGGTGAATACGTTCCCGGGCCTTGTACACACCGCCCGTCACACCATGGGAGTTGGTTCTACCCGAAGGTGCTGTGCTAACCGCAAGGAGGCAGGCaA

>contig111176
AGAGGGAATGATGTGGAAACATGTCAGTCTTCGGACCGTCTACAAGGTGCTGCATGGCTG
TCGTCAGCTCGTGCCGTGAGGTGTTGGGTTAAGTCCCGCAACGAGCGCAACCCCTATCTT
TAGTTGCCAGCGAGTAATGTCGGGGACTCTAAAGAAACTGCCTACGCAAGTAGTGAGGAA
GGCGGGGACGACGTCAAG

>contig111031
GGTCTGACAGCTTTAGAGATAGAGCCTCCTTCGGGCAGAtCACAAGGTGCTGCATGGTTG
TCGTCAGCTCGTGCCGTGAGGTGTCAGGTTAAGTCCTATAACGAGCGCAACCCCTGTtGT
TAGTTGCCAGCGAGTCATGTCGGgAACTCTAACAAGACTGCCAGTGCAAActGTGAGGAA
GGTGGGGATGACGTCAAA>contig70570
GGTTAAGTCCAGCAACGAGCGCAACCCCTGTCACTAGTTGCCATCATTAAGTTGGGGACT
CTAGTGAGACAAACTCTCTTTGAGAGTGGGAAGGTGGGGACGACGTCAAGTCAGTATGGC
CCTTACGTCTAGGGCTGCACACGTGCTACAATGCCCAGTACAGAGGGAAGCAATACCGCG
AGGTGGAGCCAATCCTTAAAGCTGGGCCCAGTTCAGATTGGAGTCTGCAACTCGACTCCA
TGAAGTTGGAATCGCTAGTAATGGCGCATCAGCTACG

>contig33189

ATACAAACACTTTGTTAATGAATTTGAGTGAACAGTAGTTAGAGTCAGAAATAGATTAAACTGAAGAGTTTGATCCTGGCTCAGATTGAACGCTGGTGGTATGCTTAACACATGCAAGTCGAACGGTAACAGAAAGAGCTTGCTCTTTGCTGACGaGTGGCGGACgGGtGAGTAACGCGTAGGAATCTACCTATCTGTggGGGATACCGTTTGGAAACGAACGTTAATACCGCATAATATCtTCGGATTAAAAgtGGCGCTTGCGCTGCTGCGGATAGATGAGCCTGCGTTAGATTAGCTTGTTGGTGGGGTAAAgGcCTACCAAGGCTGCGATCTATAGCTGAtTTgAGAGGATGATCAGCCACATTGGAACTGAGACACGGTCCaAACTCCTACGGGAGGCAGCAGTGGGGAATATTGGACAATggGGGCAAcCCTGATCCAGcCATgCCgcGTGTGTGAAGAAGGCTCTAGGGTTGTAAAGCACTTTcAGTAGGGAGGaaAAGtTAgtagTTAATAtCTGCTAGCCGTGACGTTACCTACAGAAGAAGCAcCGGCAAA

>contig57313

CACTGGGACTGAGACACGGCCCAGACTCCTACGGGAGGCAGCAGTGGGGAATCTTaGACAATGGGCGCAAGCCTGATCTAGcCATGCCGCGTGaGTGAtGaAGGCCTTAGGGTcGTAAaGCTCTTTCaGccagGGaTGATAATGACaGTACCtgGaaAAGaAACCCCGGCTAACTCCGTGCCAGCAGCCGCGGTAATACGGAGGGGGTTAGCGTTGTTCGGAATTACTGGGCGTAAAGCGcACGTAGGCGGAtTAGTAAGTTAGaGGTGAAATCCCAGGGCTCAACCCTGGAACTGCCtTTaAtaCTgCTaGTCTTGAGTTCGAGAG

>contig125053
TACTTCGCGAGAAGATGCTAATCCCAAAAAACCGTCTCAGTTCGGATTGCACTCTGCAAC
TCGAGTGCATGAAGTTGGAATCGCTAGTAATCGTAGAACAGCATGCTACGGTGAATACGT
TCCCGGGCCTTGTACACACCGCCCGTCACACCATGGGAGTTGGTTTTACCTTAAGACGGT
GT

>contig53383
ttactgggcgtaaagggTCTGTAGGTGGTATAGTAaGTCAGATGTGAAAGCCCAGGGCTC
AaCCTTGGAACTGCATTTGATACTGCtAAACTAGAGTATAGTAGAGGAATGGGGAATTTC
TGGTGTAGCGGTGAAATGCGTAGAGATCAGAAGGAACACCAATGGCGAAGGCAACATTCT
GGACTAATACTGACACTGAGGGACGAAAGCGTGGGgaTCAAACAGGATTAGATACCCTGG
TAGTCCACGCTGTAAACGATGAGtACTAGCTGTTGGGTTCGGTGTAAaGGATCTAGTGGC
GCAGCTAACGCGTTAAGTACTCCGCCTGGGGacTACGGCcGCAAGG

>contig62741

CGTGCGCAGGCTGTTTTGTAAGTCAGATGTGAAATCCCCGAGCTCAACTTGGGAACTGCGtTTGAAACTACAAGACTAGAATAGGTCAGAGGGGGGTAGAATTCCACGTGTAGCAGTGAAATGCGTAGAGATGTGGAGGAATACCAATGGCGAAGGCAGCCCCCTGGGATCATATTGACGCTCATGCACGAAAGCGTGGGGAGCGAACAGGATTAGATACCCTGGTAGTCCACGCCCTAAACGATGTCAACTAGTTGTTGGTGGAGTAAAATCCATGAGTAACGTAGCTAACGCGTGAAGTTGA

>contig180839

GGACAATGGGCGGGAGCCTGATCCAGCCATGCCGCGTGCAGGAAGACGGTCCTATGGATTGTAAACTGCTTTTGTACGGGAAGAACAAGGGGCACGTGTGCCCCTCTGACGGTACCGTAAGAATAAGGAT

>contig66510

AACCCTTGACATACTCGTCGTCGCTCCAGAGATGGAGCTTTCAGCTAGGCTGGACGAGATACAGGTGCTGCATGGCTGTCGTCAGCTCGTGTCGTGAGATGTTCGGTTAAGTCCGGCAACGAGCGCAACCCACgTcCTTAGTTGCCAGCAgGTTaaGCTGGGCACTCTAGgGaAACTGcCCGTGATAAGCgGGAGGAAGGTGTGGATGACGTCAAGTCCTCATGGCCCTTACGGGTTGGGCTACACACGTGCTACAATGGCATCTACAGTGagTTAATCtcCAAAAGATG

>contig180838
CCAGCCACTTCGCGAGAAGGAGCTAATCCCTAAAAGCCGTCTCAGTTCGGATTGcACTCT
GCAACTCGAGTGCATGAAGTTGGAATCGCTAGTAATCGCAGATCAGCATGCTGCGGTGAA
TACGTTCCCG

>contig79467_uncultured_bacterium_gamma?

GACAGGTGCTGCATGGCTGTCGTCAGCTCGTGTTGTGAAATGTTGGGTTAAGTCCCGCAACGAGCGCAACCCCTATCCTTaTTTGCCAGCGCGtTATGGCGGGAACTCTAAGGAGACTGCCGGTGATAAACCGGAGGAAGGTGGGGACGACGTCAAGTCATCATGGCCCTTACGGGATGGGCTACACACGTGCTACAATGGCAGGTACAGAGGGCAGCAATACCGCGAGGTGGAGCGAATCCCACAAAGCTTG

>contig190805
CAGAGAGCAGCCACTGGGCAACCAGGAGCGAATCTATAAAACCGGTCACAGTTCGGATCG
GGgTCTGCAACTCGACcCCGTGAAGCTGGAATCGCTAGTAATCGGATATCAGCCATGAT

>contig200053
CGAAGCACATGGCCTTACCCGCAAGGGaGGGAGTGTTCGAAGgtGGGGGTGGCGATtGGG
gTGAAGTCGTAACAAGGTAGCCGTACCGGAAGgtGCGGCTGG

>contig175833

TACCGCGAGGTGGaGCGAAACTCAGAAAGGTATTCTTAGTCCGGATTGCAGTCTGCAACTCGACTGCATGAAGCAGGAATCGCTAGTAATCGCAGGTCAGAATACTGCGGTGAATACGTTcCCGGGTCTTGTACA

>contig56197

TGACATGTATGGATTAACTCCTGGAAACAGGAGCAACGCCCTTGGGTGGAACATACACAGGTGCTGCATGGCTGTCGtcAGCTCGTGCTGTGAAGTGTCGgGTTAAGTCCCTTAACGAGCGCAACCCCTATCGTTAGTTACTAACGCGTCATGGCGAGGACTCTAGCGAGACTGCCGGTGTCAAACCGGAGGAAGGTGGGGATGACGTCAAGTCCTCATGGCCCTTATGTCTAGGGATGCaAACGTGCTACAATGGTATGGACAAAGCGATGCAATACCGCGAGGTGGAGCAAATCGCAAAAACCATGCCCCAGTTCGGATAGCAGGCTGAA

>contig181811

CGGCGCCGTGAATACGTTCCCAGGCCTTGTACACACCGCCCGTCACGTTATGGAAGCCGGTCTTGCCCGaAGTATGTTAGCTAACCCGCAAGGGAGGCGATGTCCTAAGGTGAGGCTGGTaACTGGaAC

>contig83796
TTGTTGGTGAGGTAACGGCTCACCAAGGCAAAGACGGGTAGCTGGTCTGAGAGGATGATC
AGCCACACTGGAACTTAGACACGGTCCAGACACCTACGGGTGgcAGCAGTTTCGAATCTT
TCACAATGGGCgAAaGCCTGATGGAGCAACGCCGCGTGGGGGATGAAGGCCTTCGGGTCG
TAAACCCCTGTCACCAAGGATAAAACGCTGCcTATTAATACTAGGTAGccTGATGTAACT
TG

>contig36376
CCTAGTTCTTCACTTTTCGAAGTGAGAACAAAATAAAACGCTTAATGAATGATGTTCATG
CAAATAAATACAGTTACTTATCTTTAGTTAGGTAGGTAACGACTATGTAATGCGATATCA
GCTTCGGTTGGTATCACGACAGAATTCATTGAGCAGTAACACATCATTTATGGTGAGGTT
ACACAAACGATTTTTAATTGAAGAGTTTGATCATGGCTCAGATTGAACGCTGGCGGCAGG
CTTAACACATGCAAGTCGAGCGGTAACATTCctaGCTTGCtaGgAGATGACGAGCGGCGG
ACGGGTGAGTAATGCTTGGGAATATGCCTTATGGTGGGGGACAACAGTTGGAAACGACTG
CTAATACCGCATAAcGTCTACGGACCAAAggaggggATCTTCGGACCTTtCGCCATTTGA
TTAGCCCAAGTGAGATTAGCTAGTTGGTGAGGTAATGGCTCACCAAGGCGACGATCTCTA
GCTGGTTTGAGAGGATGATCAGCCA

>contig51166

GTTGGCATTGGGGCGGTCGTCTGAATGATATTGTGCAGAGCGGCTCCTTGAGATGAGCTGGTTGATCGTGGAATTCCACTGCGATTGGCTgGtGACATAAACTTGAGAGTtTGATCATGGCTCAGAATGAACGCTGGCGGCATGCCTAACACATGCAAGTCGAACGAAGGcTTCgGcCTTAGTGGCGCACGGGTGCGTAACGCGTGGGaATCTGcCCCTTGGTTCGGAATAAcAgTTGGAAACGAcTgCTAATACCGGATGATGACGTAAGTCCAAAGATTTATCGCCGAGGGATGAGCCCGCGTAGGATTAGGTAGTTGGTGTGGTAAAGGCGCACCAAGCCGACGATCCTTAGCTgG

>contig180825

GATTAAACTGAAGAGTTTGATCATGGCTCAGATTGAACGCTGGCGGCAGGCtTAACACATGCAAGTCGAGCGtGAAAGCACTTCGGTGtGAGTAGAGCGGCGGACGGGTGAGTAACGCGTAGGAATCTACCT

>contig181784

GGACAATGGAGGAGACTCTGATCCAGCCATGCCGCGTGCAGGAAGACGGCCCTATGGGTTGTAAACTGCTTTTATAtGGGAAGAAACACCTCTACGTGTAGAGGCTTGACGGTACCATAAGAATAAGGAC

>contig155078
GGGTTAAGTCCCGCAACGAGCGCAACCCTCGCCTTTAGTTGCCAGCATTAaGTTGGGCAC
TCTAGAGGGACTGCCGGTGATAAGCCGGAGGAAGGTGGGGATGACGTCAAGTCCTCATGG
CCCTTACGGGcTGGGCTACACACGTGCTACAATGG

>contig169085

GACAATGGGCGAAAGCCTGATCCAGCCATGCCGCGTGTGTGATGAAGGCCTTAGGGTTGTAAAGCACTTTCAACGGTGAAGATAATGACGGTAACCGTAGAAGAAGcCCCGGCTAACTTCGTGCCAGCAGCCGCGGTAATAC

>contig145309

AGCTTACTGGACGATTACTGACGCTCAGGCTCGAAAGCATGGGGAGCGAAAGGGATTAGATAcCCCtGTAGTCCATGCCGTAAACGTTGTACACTAGGTTTCGGGACATTCGACCGTCTCGGAGCTCAAGCTAACGCGATaaGTGTACCGCCTGAGGACTA

>contig89951

TAGCCGTACCGGAAGGTGCGGCTGGAACATCTCCTTTCTAGAGTGTCTCTTGACACCAAGTACCGTAAAACGACAATCTTTGTTGTTTCCATTTCTCGATAATAtTTAAAAAAGGaAAATCCtCCTAACAtTTGTTAGAAGCGTAGGAGGGAtaaaTAGTCCCGTAGCTCAGTTGGTTAGAGCACTACACTGATAATGTAGGGGTCAGCAGTTCAAATCTGCTCGGGACTAC

>contig86408

TAGCCGTACCGGAAGGTGCGGCTGGAACACCTCCtTTcTAGAGAATTTGCCCATGTCGGCTTTTTCAAGGGCCCGTTTTTTCGATTTCTGTTTTTACAACATAAAAAACTATAGTGAGTCTCATAGCTCAGCTGGTTACCCGcCCGCGCCGAAAGGCACGTTCGGACGGGGagcGCGGCGCCCTTATAAGGAATTATCGGGGCGGTTGTCCACAGAAAGGCTATTAGGGATTTGAAC

>contig78263

CGGCAGGCCTAACACATGCAAGTCGAGGGGTAACATGATTGAGCTTGCTTAATTGATGACGACCGGCGCACGGGTGCGTAACGCGTATGCAACCTACCTTATACTGGGGAATAGCCAGAAGAAATTCTGATTAATGCCCCATAGTATTTATTAACCGCATGGTTTTATAAATTAAAGATTACGGTATAAGATGGGCATGCGTCCTATTAGCTAGATGGTAAGGTAACGGCTTACCATGGCAACGATAGGTAGGGGG

>contig61822

CGTAGTTCAAGAATCCATGGATTCATGaACTCTCTAtTTTCGGAGAGTTTGATCCTGGCTCAGAATGAACGCTGGCGGCGTGGTTCAGACATGCAAGTCGAACGAGATTGTCCAGCTAGCTTGCTAATTGGACATGACAGTGGCGAACGGGTGCGTAACACGTAAAGAACCTACCCTTATGTGGGGGATAGCTCACCGAAAGGTGAATTAATACCGCATGTGGTCTCTCTTCTCATGAAGAGTACACTAAAGCTGGGGACCTTCGGGCCTGGCGCATAGGGAGGGCTTTGCGGCCTaTCAGCTTGTT

>contig26725

TCGAGACAGCAGATACGCTTTTAAAGCGTTGAAAACGGCTTCGAAAACAGCTTTAAAACTTGATCAAAAATAAGTTTACGAAAGGTGTTGACGgGCTGGCTAGAATGAGTAGAATGCACCAACCTTGAGACAGCGAAGCAAGGGCTTGAAACTAAGCACTTGTAACGGTTTaCaACGGTTTGAATTATTTCAAAAAGTTGTTGACAAAGCTTTTCAAGCGAGTATGATTCACCTCCCTTGAgGCAAcTCAAGAAGCGCCAATAAGGCGCTAGAGTTTGAAATTAGGTTAACCCTTAAAATCAAACGCCGCTCTTTTAAAAATCAAACCAAACAATTTGTGTGGACGTTTTCTTAAATGAATAGGTTGCACAAAAACCGGTAAACCAAGTTTTATGTTATCGCAAGATGGCAGCTCATTCCGAGAAACTTGAGAGACCCAGCTTTTATTATTTTTGGCTGTAAAACAAAAAATTCCTTTAGTAATTAGATTGAACTGGAGAGTTTGATCCTGGCTCAGATTGAACGCTGGAGGTATGCTTAACACATGCAAGTCGAACGCGAACGTTCCTTCGGGAGCTATTAGAGTGGCGGACGGGTGAGTAACGCGTAGGAATCTACCTAAGTGTGGGGGATAACATGGAGAAATTCATGCTAATACCGCATACGCCCCACGGGGTAAAGAGGGCCTCTTCTTGAAAGCTCTTGCATTTAGATGAGCCTGCGTCGGATTAGCTT

>contig130196
AGGGAgtGCTTGcacTCCGCTGACGACcGGCGCACGGGTGCGCACCGCGTATGGAACCTA
CCTTTTAcaGGGGAATAGCCTTTGGAAACGAAGATTAATGCCCCATTGTACCTTTTGTAG
CATTGCAAGACGGTTAAAGACTTCGGTCGGTAAAAGATGGCCATGCGTCCCATTAG

>contig183699

GGGTTAAGTCCCGTAACGAGCGCAACCCTTGTCCTTAGTTGCCAGCaCgTaATGGtGGGAACTCTAAGGAGACTGCCGGTGACAAACCGGAGGAAGGTGGGGACGACGTCAAGTCATCATGGCCCTTA

>contig159711

GGATCGGCTTCTtTTGATGCATAtcAGTTTCTATTGACGCAAGTCATGGAGGCTGTGCAAAAAATATCAACTGAAGAGTTTGATCCTGGCTCAGATTGAACGCTGGCGGCATGGCTAAAACATGCAAGTTGTACGACCTCTTCGGAggG

>contig180813

GCCGCCAGCGTTCGTTCTGAGCCAGGATCAAACTCTCAAGTTGAGAAtTcGATTTTGGCaTTTaTGGTCACGCtTgaAtcGACGAGAACTTCACACCTAAACCAACATCAATAAATCAATGCCAGTCCAGT

>contig106034
CGTTCGTAGGCGGTTATTtAAGCAAGATGTGAAAGCCCAGGGCTCAACCTTGGAACTGCA
TTTTGAACTGGgTAACTAGAGTACTGTAGAGGGTGGTGGAATTTCCAGTGTAGCGGTGAA
ATGCGTAGAGATTGGAAGGAACATCAGTGGCGAAGGCGGCCACCTGGACAGATACTGACG
CTGAGGAACGAAAGCGTGGGGAGCG

>contig142746

GCGAGGAATATAATCGAATGTACCCCAAGGTAAAGGATAGCTTCTGGAAACGGGAGATAATACTTTATGTGCTCTACGGAGGAAAGGTTTACCGCCTTGGGAGCGGCGATTATcCTATCAGGTAGTTGGTGAGGTAAAGGCTCACCAAGCCGAAGACGGGTAGC

>contig163243

AAACTACCTTTCGGTACGGAACAACAGTTGGAAACGACTGCTAATACCGTATACGTCCTTAGGGAGAAAGATTTATCGCCGATAGAGGTGCCCGCGTTGGATTAGCTTGTTGGTGAGGTAATgGCTCACCAAggcgacgatccatag

>contig40007
TTAATGCTTGACTGGCTTCAAAaCCCCTGTACAATGCGCACCTCGTTAACGCAAAGCGAT
tAACGATTGAGCCGgAAGGCACTGTTCTTTAACAAATTGAATGACTGATAAGTGTGGGTG
TTTGTGACTGAGTtATAAGCATTATATTTTCGGATATGATGTACTCAAGATACAAATGCT
TACACTTAAATTTATGACAAGTATGTAATAACgCATtagTTAATTTATTAGCTAaTGTGT
ACAAGACACACCTTGGAAATGAATTTGAGTTTTTATAGAGAGCAATCTTTATAGATATAA
AAGCAAAAGCGAAATACCACCTCGCAAGAGGAATAAGTAATAGTTGTGAATTAAACTGAA
GAGTTTGATCATGGCTCAGATtGAACGCTGGCGGAATGCTTTACACATGCAAGTCGAACG
ATGAACCTTAGCTTGCTAGGGGGATTAGTGGCGG

>contig19040

TAGCCGTATCGGAAGGTGCGGCTGGATCACCTCCTTTCTAGAGACTTTGACTTTAGTTGCAAGCATTCACACTTATCAGTCGTTCGGTATTAGCAGTATCAAAGATTGGCCAAACGATTAGCGTTGATGCTAATTGAGCCTTTAATTAGGGTCTGTAGCTCAGCTGGTTAGAGCACTGTGTTGATAACGCAGGGGTCGATGGTTCGAGTCCATCCAGACCCACCAGATATCACCTTATATGGGGGATTAGCTCAGCTGGGAGAGCACCTGCTTTGCAAGCAGGGGGTCAACGGTTCGATCCCGTTATCCTCCACCAATATTATTTGGAAGATCTTTGATTAAGTAAAACCTAGAAGCAAGTCTTGTGAGGGTAGTTTAAATCACAATATTTGCTTCTAGCTTTTTAGCTAGACTGTTCTTTAACAAAATGGAAGAAGTaAAgAGAATATACAAGTTGTGATGACTTGCTATATTCAAATGGGTAATATTATCTTAGTCGGATAATATTATTGATTGCAAAATCGAAAATCATTTCGCTTTGTTGTGTTTAATTGAACGTTAAGTTTGATTGAAGACGATTGAAGTTGAAATAACAAACCAGTCATTATTTTAATAACCTTGGAATAATGACGAAGTTGCTTTAGAAAACCTATAGCGGGCGTCTCATGCGCCGCGAGATTTGAATTTAAAAGGGTTCAGGTTTTAACGTTATAGGGTCAAGTGAATAAGTGCATATGGTGGATGCCTTGGCGATTACAGGCGATGAAGGACGTGATAATCTGCGAAAAGCTTCGGGGAGCTGATAAATAAGCTTTGATCCGGAGATGTCCGAATGGGGaAAcCCACCCGCAAGGGTAACCGCTCCTGAATATATAGGGAGATGGTGGCAAACCGAGTGAACTGAAACATCTAAGTAGCTCGAGGAAAATAAATCAACCGAGATTCCGTAAGTAGTGGCGAGCGAACGCGGAACAGCCTGTTATTTTTAGCACATGCGATAGTAGAACGGAATGGAAAGTCCGGCCATAGAGGGTGATAGCCCCTTATACGAAATCCCGTGTGTGGAACTAGGATAACGACAAGTAGGGCGGGGCACGAGAAACCTTGTCTGAAC

>contig165535

CGCGCgTAGGCGGTTTGATAAGCTGGATGTGAAAGCCCTGGGCTCAACCTGGGAACTGCATCCAGAACTGTCTGACTAGAGTACAGTAGAGGTGAGTGGAATTTCCTGTGTAGCGGTGAAATGCGTAGATATAGGaAGGAACATC

>contig118828
CGCGCGTAGGCGGTTtGTTAAGTCGgATGTGAAAGCCCTGGGCTCAACCTGGGAACTGCA
TTCGATACTGGCAaCTAGAGTATGaaaGAGGGaGgTaGAATTCcaTGTGTAGCGGTGAAA
TGCGTAGATATagtGgAGGAAtACcAGTGGCGAAGGCGGCctcCTGGttCAATACTGACG
CTGAGGTGC

>contig109980

TACAGAGACTCCAAGCGTTATTCGGATTCACTGGGCGTAAAGGGAGCGCAGGCGGCCAGATGTGTCAGATGTGAAATACTGCAGCTTAACTGTAGAACTGCATTTGAAACTATCTGGCTAGAGTATCGGAGAGGTAAGCGGAATTCCAGGTGTAGCggTGAAATGCGTAGAtATCTGGAGGAAtACCggTGGCGAAGGC

>contig99246

aGCTtGCTTATCCGCCGGCGACCGGCGCACGGGTGCGCAACGCGTATAGAATCTGCCCTG
TACTGGGgAATAGCCCAGAGAAATTTGGATTAATGCCCCATGGTATGTATTAGCGGCATC
GCTTATACATTAAAGGCTACGGTACAGGATGACTATGCGTCCCATTAGTTAGTTGGTAAG
GTAACGGCTTACCAAGGCAGCGATGGGTAGGGGC

>contig96763

AGCGCTtCCCTTCGGGGaGGAGCGGCGGACGGGTTAGTAACGCGTGGGAACATACCCTTT
tCTACGGAATAGCCTCGGGAAACTGAGAGTAATACCgTATACGCCCTTCGGGGGAAAGAT
TTATCGGgAAGGATtGgCCCGCGTtAGATTAGATAGTTGGTGGGGTAATGGCCTACCAAG
TCTACGATCTATAGCTGGTTtgAGAGGATGATCAGCAA

>contig183980

CCAGCCATGCCGCGTGTGTGAAGAAGGCCTTCGGGTTGTAAAGCACTTTAAGCGAGGAGGAATGGCTCTGGGTTAATACCCTTGAGCAGTGACGTTACTCGCAGAATAAGCACCGGCTAACTCTGTG

>contig90508

CATAGtCtgaCttTCtTCGGaaAGAAcGaCAAAAAAACGCTTTAATGAATGaaGTTCACATAAATGAATacTTTTCTTCGGaaaagTAATACTTATATATGTAATGCGTATCAAACTTGTTTTGATACACGACAGAATTCATTGAGCAGATGTTCACTTTTGTGACATCACaAACGATTTTTaATTGAAGAGTTTGATCATGGCTCAGATTGAACGCTGGCGGC

>contig186429

GACAATGGGCGCAAGCCTGATCCAGCAATACCTCGTGTGTGAAGAAGGCCTTAGGGTTGTAAAGCACTTTCAATTGGGACGAAGGGAGTAAGTTTAATACGCTTATTTCTTGACGGTACCTTTAG

>contig153971
TACTATGGGATAGCCCAGAGAAATTTGGATTAATACCATATAGTATCATATTACGGCATC
GTATTTATGATTAAAGGTTACGGTAAGAGATGAGTATGCGTCCTATTAGTTTGTTGGTAA
GGTAACGGCTTACcAAGACTACGATAGGTAGGGGC

>contig152750
ttgcgcaatggaggaaactctGACGCAGCCATACCGCGTGTGTGAaGAAGGCCTTAGGGT
TGTAAAGCACTTTCAGCGAGGAGGAAAGGgTGTAGGTTAATAGCcTGCATCTGTGACGTT
ACTCGCAGAAGAAGCACCGGCTAATTCAGTGCCAGC

>contig118835

CGCGCGTAGGTGGTTTGTTAAGTTAGATGTGAAAGCCCTGGGCTTAACCTAGGAATTGCATTTAAAACTGGCTAACTAGAGTATGGTAGAGGGAAGTGGAATTCCACATGTAGCGGTGAAATGCGTAGAGATGTGGAGGAACACCAGTGGCGAAGGCGACTTCCTGGACCAATACTGACACTGAGGTGC
