# Supplementary material for: Microbial Consortium Associated with the Antarctic Marine Ciliate Euplotes focardii: An Investigation from Genomic Sequences
Source: Microb Ecol. 2015 Feb 24;70(2):484–97. doi: 10.1007/s00248-015-0568-9 (PMC4494151; doi:10.1007/s00248-015-0568-9)
Supplement: Supplementary file 3 — (DOC 46 kb) [file 248_2015_568_MOESM3_ESM.doc]

**Table S1**. Amino acid sequences used as query to explore the *Euplotes focardii* bacterial consortium dataset by tBLASTn for the identification of antifreeze proteins or enzymes involved in bioremediation.

| **Query sequences** | | **Amino acid sequence** |
| --- | --- | --- |
| **Antifreeze** | | |
|  | ***Colwellia* sp. SLW05 ice-binding protein (ABH08428)** | MKTLISNSKKVLIPLIMGSIFAGNVMAAGPYAVELGEAGTFTILSKSGITDVYPSTVTGNVGTSPITGAALLLNCDEVTGAMYTVDSAGPLPCSINSPYLLELAVSDMGIAYNDAAGRVPADHTELGTGEIGGLTLEPGVYKWSSDVNISTDVTFNGTMDDVWIMQISGNLNQANAKRVTLTGGALAKNIFWQVAGYTALGTYASFEGIVLSKTLISVNTGTTVNGRLLAQTAVTLQKNTINAPTEQYEEAPL |
|  | ***Marivirga tractuosa* DSM4126 antifreeze-related protein (YP_004052221** | MKLIKTMPILAMMLFMLMVSCKDKDNNDDDPTPSEDVIPTVLSTMPENNDIDVARNHNVTITFSEAMDPSTINSATFTVEQGTTDISGSVVYSGVEATFTPSVAFSAAREFTATISTTAKSQKGINIEENYEWSFTTSGSESTISGIDLGASGNYVILSKTAINNSGTSSITGDLGLSPAATSYITGLALTDFTGYATSAQVTGEVFASDMADPTPINLTTAVENMITAYNNAAGRSETDFLELGAGNIGGLTLTTGVYKWTNTVTIPTNVTISGSSTDVWIFQIAQDLTMSSATNIILTGGAQAKNIYWQVAGQATLGTTSHFEGIILSMTGITFQTGASINGRALAQTAVVLDANAVVQPQ |
|  | ***Rhodobacteraceae bacterium* HTCC2083 antifreeze protein, type I (WP_009830968.1)** | MGIFDFLTGEFIDVIHWTDDTRDTMVWRFEREGHEIKYGAKLTVREGQAAVFVHEGQMADVFTPGLYLLETNNMPILTTLNHWDHGFQSPFKSEVYYVNTTRFNDLKWGTKNPIIARDPEFGPVRLRAFGTYSVKVSDPARFLTEIVGTDGEFTMDEISFQIRNIIVQEFSRTIARAGIPVLDMAANTRELGQLVAREIEVQLAEYGLSLPELYIENISLPPAVEAVMDKRSSMGVIGNLNEYIQFQAAEALGRDGGGAAAIQAGVGAGLGMQIAGTMAQQVGPWGARSTSAPATTAPMAPPPPPPVEHVWHIAANGETTGPFSKAKMGRMATEGTLTRDSLVWTAGQDGWKAAQEIIELAQLFTIMPPPPPPAA |
|  | ***Lates calcarifer*** **type II antifreeze protein (ABV66064.1)** | MLTVSLLVCAVMALASADDNSTTSIVDVSVAEDNSTTSLDVNAAEDNSTTSKKEEVAPSCEIGWSEFNGRCFLFVSTEMSWADAEKNCLLKKGHLASVHNEEEYKHIQAVVNAHTGGHPTTWVGGSDCQKEGIWLWSDGSGFEFDSWCEGEPDNYVGAESCLQINANESHCWNDFPCSTVLPSVCASVPQSS |
|  | ***Flavobacteriaceae bacterium 3*519-ice-binding protein (YP_003095014.1)** | MNKFLLLAASVAFMSFSGKAHAQAPTLGAAANFALFTTAGAVTNTGLSHITGDVGTNNAASTNFGNVDGVMQDSNGATSAAAADLLIAYNLLNAAIPTATLAPLLGNGTTLTAGNYFIGQGASLSGTLTLDGGGNSNSVFIFKIQGALSSAANTQVLLTNGALACNVFWKVEGLVDLATNTVMKGNVVANNAAIVLQSGVSLEGRALSTTGAITVTGVTVRKPILCGSAVLTGPVAPNLGTVVCYTIFSGNGALTNAGITYVTGDVGTNVGLTTGFQADNVNGTIHSNPDTSTAQAALDLNNAYTYLNTLPTDIELLYPAAFGQNLVLTPHTYLLNAATVLNGKVTLDAQGNENAVFVIKINGALSTTVNASVELINGAIAKNVFWKVDGAVDLNDYTKFKGSVIGNNGAVIINTGVEIEGRVLSTSGGISTFGINAQMTPGCELLGTGSNTVAIQAAKFYPNPFSSVLNVTMEDLNGGSTLTIYNAAGSQVFSKVLSTKTTSLSMKLPAGVYFYQMIGKNGAKQAGKLIAKP |
|  |  |  |
| **naphthalene dioxygenase** | | |
|  | ***Pseudomonas* sp. PZT2 naphthalene dioxygenase AAV33341.1** | KKELYGESLNKKCLGLKEVARVESFHGFIYGCFDQEAPPLMDYLGDAAWYLEPMFKHSGGLELVGPPGKVVIKANWKAPAENFVGDAYHVGWTHASSLRSGESIFSSLAGNAALPPEGAGLQMTSKYGSGMGVLWDGYSGVHSADLVPELMAFGGAKQERLNKEIGDVRARIYRSHLNCTVFPNNSMLT |
|  |  |  |
| **catechol 2,3 dioxygenase** | | |
|  | **Pseudomonas putida Mt-2 (gi|5107613)** | MNKGVMRPGHVQLRVLDMSKALEHYVELLGLIEMDRDDQGRVYLKAWTEVDKFSLVLREADEPGMDFMGFKVVDEDALRQLERDLMAYGCAVEQLPAGELNSCGRRVRFQAPSGHHFELYADKEYTGKWGLNDVNPEAWPRDLKGMAAVRFDHALMYGDELPATYDLFTKVLGFYLAEQVLDENGTRVAQFLSLSTKAHDVAFIHHPEKGRLHHVSFHLETWEDLLRAADLISMTDTSIDIGPTRHGLTHGKTIYFFDPSGNRNEVFCGGDYNYPDHKPVTWTTDQLGKAIFYHDRILNERFMTVLT |
| **NADPH-dependent F420 reductase** | | |
|  | **Colwellia psychrerythraea 34H (gi|71281606)** | MKIAILGGTGPQGRGLALRLAKAGVDVVLGSRDAARADEIAIELNQIIKPSSDELGVISGANNEDAVDAADKMVVLAVPYSAHNTTLEAIKAQLADKILVDIVVPLAEGNPKAVDMPAEGSVTEEAQALLGDDIPVVGALHNVSAHTLNKLDQAINCDILVCGNDLAAKEQVIALIQRMDVCAYNVGPAVNARCVEAITPMLIRLNISKKVPFTHSGLKIWAPGT |
|  |  |  |
| **poly (3-hydroxyalkanoate) polymerase** | | |
|  | **Pseudomonas putida (gi|326314770)** | MKDKPAKGSTTLPATRMNVQNAILGLRGRDLLSTLRNVGRHGLRHPLHTAHHLLALGGQLGRVMLGDTPYQPNPRDARFSDPTWSQNPFYRRGLQAYLAWQKQTRQWIDESHLNDDDRARAHFLFNLINDALAPSNSLLNPLAVKELFNTGGQSLVRGVAHLLDDLRHNDGLPRQVDERAFEVGANLAATPGAVVFRNELLELIQYSPMSEKQHARPLLVVPPQINKFYIFDLSATNSFVQYMLKSGLQVFMVSWRNPDPRHREWGLSSYVQALEEALNACRSISGNRDPNLMGACAGGLTMAALQGHLEAKQQLRRVRSATYLVSLLDSKFESPASLFADEQTIEAAKRRSYQRGVLDGGEVARIFAWMRPNDLIWNYWVNNYLLGKTPPAFDILYWNADSTRLPAAMHGDLLEFFKLNPLTYASGLEVCGTPIDLQQVNIDSFTVAGSNDHITPWDAVYRSALLLGGERRFVLANSGHIQSIINPPGNPKAYYLANPKLSSDPRAWFHDAKRSEGSWWPLWLEWITARSGLLKAPRTELGNATYPPLGPAPGTYVLTR |
|  |  |  |
| **polyhydroxyalkanoate depolymerase** | | |
|  | **Pseudomonas aeruginosa (gi|50235377)** | MPQPFVFRTIDLDGQTIRTAVRPGKEGSTPLLIFNGIGANLELVFPFVQALDPELEVIAFDVPGVGGSSTPSVPYRFPGLAKLAARMLDYLDYGQVNAIGVSWGGALAQQFAHDYPERCKKLILAATSAGAVMVPGKPKVLMRMASPRRYIQPSYGVHIAPDIYGGAFRRDPKLAMAHASKVRSSGKLGYYWQLFAGLGWTSIHWLHRIRQPTLVLAGDDDPIIPLINMRVLAWRIPNAELHVIDDGHL |
|  |  |  |
| **cyanophycin synthetase** | | |
|  | **Colwellia psychrerythraea 34H (gi|71281643)** | MKIQSSNVYVGPNVYAHFPVIRHIVDIGILEKYPSVRLGNEFIQGLLTHLPSLDQHGCSYGEPGGFIRRLKEDDGTWIAHIWEHVTLELQCIAGTEVTFGKTRGTGKVAEYNMVFQYKQRDVGLEAAILARNLLISLMPVAIQVELQTKIEDDFDFQQELADFIRFAQRKEFGPSTQSLVDAAEERDIPWLRLNEYSLVQFGHGKYQKRIQATITSETKHIAVEISCDKEDTHNLLNDLGLPVPQQRMVYSDTQAVRMAKRIGYPVVLKPLNANHGRGVSIDLNTEEQVITAFAFAKEQGTSRAVLVESFLTGLDHRMLVINGELVAVAKRVPGHIIGDGVNNISQLIDIVNEDPRRGVGHEKVLTQLELDTQAERLLEEADYTQGTVLPKGEIFYLRSTANLSTGGTAIDMTDVVHPDNKTMAERAVKAVGLDIGGVDFLTSDITQSYKDIGGGIVEVNAAPGFRMHVAPSEGKPRDVAGKVIDMLFPPSIPKRIPIAGITGTNGKTTTSRMLAHILKSAGHVVGMTSTDGVYVDGQLSVKGDMTGPVSSQIVLRDPSVDIAVLETARGGIARSGLGYNECDVAACINVQEDHLGLRGIDTLDQLAEVKRIVVEVAKDSVVLNADDPQCLKMAEHTKAKHLCYVTMNTGHSLVREHIRAGGRAVVLEKGINGDMITIFDNGTHIPLLWTHLIPATLEGKALHNVQNAMFAAALAYCLDKPLEAIQQGLRTFTTTFYQAPGRMNVFDEHHFRVILDYAHNADGVRCMSELASKLEVKGKRITVLAGPGDRRDEDIVNIAKAAAGHFDIYICKADDNRRGRGVNEVPELLASSLRAEGIDESQIYCISDEVEAINKGLELANTDDLLMIFGDAITRCWKQIINFNSGHEPEQEAEKTAVQTVVSMLETSEPDTFVLESGMKIVTDERGVRVVSEHDEDSD |
