# Supplementary material for: Microbial Consortium Associated with the Antarctic Marine Ciliate Euplotes focardii: An Investigation from Genomic Sequences
Source: Microb Ecol. 2015 Feb 24;70(2):484–97. doi: 10.1007/s00248-015-0568-9 (PMC4494151; doi:10.1007/s00248-015-0568-9)
Supplement: Supplementary file 4 — (DOC 53 kb) [file 248_2015_568_MOESM4_ESM.doc]

**Table S2**: List of the contigs, and corresponding predicted amino acid sequences, resulting from the tBLASTn operation for the identifications of enzymes involved in the recycling of organic substances.

| Enzymes | contigs | predicted amino acid sequence | Blastp results |
| --- | --- | --- | --- |
| catechol 2,3 dioxygenase | contig43371  contig100280 | >contig43371  MRTVTSDNITQAFIDYMADDTDERVKFLMTKLVEHIHDFVRETNLSHDEWRKALELLFRAGEISDSERNEFVLFSDVLGLSSIVDMINSPDDGTPSSVLGPFHVLGAPDLPIGADMKGDNEGDLVVVGGVVKSTDGSPIQGATLEIWQTADNGLYSGQDEAQPEYNLRARMKTDGDGRYLFSTVRPAPYVVPDDGPVGDLLRATGRHPWRPSHLHFIATADGYRQLVTEVFPSDDIYLDEDAVFGVREKLVMEYQERDDASGLPDDLAIKDKISGKFFAVEFDFTLSPSA | 77% identical with the catechol 2,3 dioxygenase from Rhodobacteraceae bacterium HTCC2150 (e-value: 2e-133) |
|  |  | >contig100280  MKYSNFSRRHFLGASSLAGAAFAFTAAGQSALKVTPPHTEGPFYPIVEQDDKDTDLTLYGDSKVPANGEIITVEGLILDDSNAPIANAVVDIWQANAVGRYAHERDPNSAPLDPNFQGWAIIKTDSEGRYLFKTVKPGAYPVSENWTRPPHIHFKVSRRGYREITTQMYFDDEPLNDIDKLLNELPTDLQSTLIATRKSKAESFKFNIVLAMV | 50% identical with the catechol 2,3 dioxygenase from Pseudoalteromonas luteoviolacea HTCC2150 (e-value: 6e-61) |
|  |  |  |  |
| naphthalene dioxygenase | contig00329 | METYTNNPKAVADLVNGHQVHRDVYISDEVYKLEMQHLFANTWVFVGHESQTANKGDYFTTQVGDQPVIQVRHTDGEIKVLYNRCPHKGTKIAIDRQGNTGKFFRCPYHAWSFKTDGCLLAIPLKKGYEGTGLKETESAKGIKAVGAVENYRGFIFARLAHEGISFADFFGGSLSSIDNMVDRSPAGRLVVAGPPLRYMHQCNWKMLVENQTDTCHPMVAHESSAGTAIRLWEEMDMPADAPKPPAMEIIAPFMSSYEFFEGMGIRTWPNGHGHTGVNHSIHSDYSVIPGYEDAMIANYGEEKAHKILGENRHNTVYFPNIMIKGPIQQLRNFIPMGPNKTLVESYIYRLVDSPDELLARTAMYNRMINAPTSIVGHDDLEMYERAQEGLHSDGLKWVNIQRLHTEDEDFNQEKVENGTTERQMRNQFDAWRKFMTASMEQTPQETTE |  |
| co-enzyme F420 reductase | contig56385  contig87840  contig176575 | >contig56385 (partial)  AGTLKLVKPALQGKILIDVTVPLVPPKVARVQLPEQGSAGQIAQELLGEEVSVVSAFQNVAAAHLQEGHGLECDVLVSGNKKAAREEVIKLVEAAGMRGFHAGMINNAA | 95% identical with the F420-dependent NADP reductase from the marine gamma proteobacterium HTCC2148 (e-value: 3e-61) |
|  |  | >contig87840 (partial)  PFAHQQGTLELVKDALKGKILIDVTVPLVPPKVARVQLPEQGSAGQIAQNLLGEDVQVVSAFQNVAAHHLQEGQGLSC | 85% identical with the F420-dependent NADP reductase from the marine gamma proteobacterium HTCC2148 (e-value: 2e-36) |
|  |  | >contig176575 (partial)  SVECDVLVCGNKKAAREQVIALVEAAGMRGYHAGMINNAAAAEA | 97% identical with the F420-dependent NADP reductase from the marine gamma proteobacterium HTCC2148 (e-value: 2e-18) |
|  |  |  |  |
| poly(R)-hydroxyalkanoic acid synthase | contig17932  contig14420  contig32897  contig31257  contig73789  contig49695  contig32713  contig83948  contig73825  contig37865  contig103421  contig106359 | >contig17932 (partial)  FKQAYLLTARWAHDTIANTADTDEATRHRANFYFDQIANAMSPSNFAVTNPEVLKQTLASNGQNLLDGMNNLARDLEAGHGSLRIKQTDMSAFALGENIALSPGQVVYQNELMQLIQYAPTTDKVYQTPLLIVPPWINKFYILDLNPKKSFIRWCVEQGQTVFVISWVNPDARLAAKGFADYMHQGILEAISQIENQTGESKVNAIGYCIGGTLLASTLGFMAARKDKRIKSATFFTTQVDFTYAGDLKVFADEDQIAMVEQRMAKDGFLDGKEMSNAFNLLRSNDLIWSYVVNNYLKGKDPMPFDLLYWNADPTRMPAATHSFYLRECYLYNNLSRGKMTLDQVRIKLGKVKVPVYNLATREDHIAPLKSVFKIGEILGGDTQLVVAGSGHIA | 85% identical with the poly(R)-hydroxyalkanoic acid synthase from Roseibium sp. TrichSKD4 (e-value: 0,0) |
|  |  | >contig14420  MSEKGQKSEKSSDDTKQQFALEPYVVKNPDEFSKNLAKMIEAAGHAASEWLKPREQGNIEETSDPSAELTNTLSKLTEYWLSDPQRAIEAQTKLFSGFMGVWSNSIQRLNDTNVPEAMDASRDKRFTDEDWSNQPFFSFLKQTYLVTSQWAEDLVTDSELDPHTKHKAEFYVKQVTGALSPSNFMISNPEVFKETIATNGANLVEGMKMLADDIRDGKGELKMRQVDGSKFVVGENLATTPGKVIARSELIELIQYTPTTDKVLKRPLLICPPWINKFYILDLNSEKSFIKWAVDQGHTVFVMAWVNPDERHKDMDWTDYIHEGLFFALDNIEKATGEKDVNAIGYCVGGTLLAA | 94% identical with the poly(R)-hydroxyalkanoic acid synthase from Ahrensia kielensis (e-value: 0,0) |
|  |  | >contig32897  GYCVGGTLLAAALGYMAQIGDKRIATSTFFTTQVDFIYAGDLKVFVDEEQLHKLEEQMHKKGYLEGSKMATAFNMLRAGDMIWPYMVNNYMRGKAPTPFDLLYWNSDATRMACTNHLFYLRNCYHENRLSQGTMRIGGKLIDMKKVTIPIYNLAAKEDHIAPALSVFEGSKYFGGNVTYVLGGSGHIAG | 94% identical with the poly(R)-hydroxyalkanoic acid synthase from Ahrensia kielensis (e-value: 0,0) |
|  |  | >contig31257  TASILSSYLKQMGSDVVNSLSLSVCMLSMRSEDMELNAFTSSGALQTARANSQEKGILEGAELAKVFSWMRPNDLVWNYHVNNYLLGEAPPAFDLLYWNSDSTNLPAQLHSDFLDLYEENLLYKGEMEVLGKKIDLADLDCDKYVTAGLTDHITPWKACYKTTQFVGGDIRFVLSSSGHIQSLVNPIANEKAQYFCADNLP | 94% identical with the poly(R)-hydroxyalkanoic acid synthase from Pseudomonas sp. GM17 (e-value: 1e-65) |
|  |  | >contig32713  LSLFAAQADFTEAGELMLFINESQVTFLEDMMWEQGFLDTKQMAGAFQLLRSNDLVWSRMVHDYLMGGRAKMNDLMAWNADATRMPYKMHSEYLRKLFLNNDFAEGRYNVDGRPVTISDIRVPVFGVGTEWDHVAPWRSVYKLHLLTDTEITFLLTNGGHNAGIVSEPGHPRRHYRMSTKQNDDPYSDP | 94% identical with the poly(R)-hydroxyalkanoic acid synthase from Leucothrix mucor (e-value: 4e-108) |
|  |  | >contig37865  PLPQDRRFQGEAWQQWPYNLMYQSFLLNQQWWHNATVGVRGVTQQHENVVEFAARQLLDVFSPSNYAFTNPEVMEKTVKEGGQNLVRGFQNFMEDWDHAVSGKKPAGTEEFKLGETLAVTPGKVVYRNRLIELIQYEPVTKKVHAEPILIVPAWIMKYYI | 74% identical with the poly(R)-hydroxyalkanoic acid synthase from Leucothrix mucor (e-value: 2e-79) |
| polyhydroxyalkanoate depolymerase | contig00480  contig22559  contig25988  contig26685  contig41271  contig19147  contig67930  contig49207  contig93044  contig105115  contig121414  contig82573  contig115699  contig138680  contig149842 | >contig00480  MFYKFYEMNHALMQPWRTMAEATGMFYRNPINPVSTTKYGRAMAAWAEVFERTTRRYGKPTFGIHETIVDDDEIAVVEKVEWEKPFCKLLHFSRALKKPRGDDPKILLVAPMSGHYATLLRGTVERLIPEAEVYITDWVDARGVPVSKGSFDLSDYIDYLIEMVEHLGPDTHIIGVCQPSVPVLAAVAVMEKRKGGVSPLSMTLMGGPIDTRRNPTAVNDLAEEKDIEWFRDNVIMDVPFPNPGLGRQVYPGFLQLTGFMSMNLDRHMIAQKDFFMHLVRDDGDSAEKHRDFYDEYLAVMDLTAEFYLQTIEVVFIEHSLAKGTMMHRGEKVDLSAIKNVALFTIEGENDDITGRGQTEAAQDLCPNIPAAFKQHYEQPSVGHYGVFNGSRFREEVAPRILEFMANSKTRKNSKRPELRAVS | 69% identical with the polyhydroxyalkanoate depolymerase from Sinorhizobium fredii NGR234 (e-value: 0,0) |
|  |  | >contig22559  QHLGENVHVMAVCQPTVPVLAAVSLMHQRKDKLLPASMTLMGGPIDTRRNPTTVNEMATERGIDWFKRHVIVRVPLPNAGFMRQVYPGFLQLSGFMTMNLDRHVDAHRELFRHLVDGDGDSAEKHEEFYDEYMSVMDLTAEFYLQTVDRVFVKQALPKGEFTYRDQLIKPASITETALMTVEGENDDISGIGQSEAAHGLCTSLPEDMRDHHLQEKVGHYGVFNGSRFRAHIVPRLVAFMHKHNAQT | 71% identical with the polyhydroxyalkanoate depolymerase from Rhodopseudomonas palustris DX-1 (e-value: 2e-123 ) |
|  |  | >contig25988  MLYMMYELNHAAVAPLRLAAKYSHQFWSNSANPIADTEFGRSYAASLGMFERLTRRYGKPEFGITATEVEGKACAVHEASIWEKPFCSLLHFNRQFETAPTDPKQHQKILIVAPMSGHYATLLRGTVEAMLPHGDVYVTDWVDARDVPGSKGSFDLDDYIDYVIEMIQHLGENVHVMAVCQ | 62% identical with the polyhydroxyalkanoate depolymerase from Chelativorans sp. BNC1 (e-value: 2e-69) |
|  |  | >contig26685  RERDIPWFEDNVLRCVSSSQPGYGRSVYPGYLQLASLMAYLNRHLMQQGELFYKIYNDDGLNPVGHSFFTLFTSVMDLPGEFFLDNISAVFHRRDICCRSLSWHGETVDPGRITTIPLMTIEGEDDDIAAPGQTSAAHGLCPLIPESARRRLLVAKCGHFSLFHGNKWRSEILPELVRFLEGHGPGSDTDQGNTCRSDFS | 40% identical with the polyhydroxyalkanoate depolymerase from Beijerinckia indica subsp. indica ATCC 9039 (e-value: 9e-42 |
|  |  | >contig19147  MKPNFDIDLVVIEGVVSTVRERVVSDKLLCSLVEFSTAQSTRFPALLLVAPLSGHYAVLLTDMIIGLLPRFRVYVTDWTNASEVPVSAGSFGLEQNIDYVLDFIGVAGEGTHVVALCQGGVPALATAAILAAQQRESDIGSLTLIGGPIDPMANPTRVVNLLRERDIPWFED | 42% identical with the polyhydroxyalkanoate depolymerase from Janthinobacterium agaricidamnosum (e-value: 2e-35) |
|  |  | >contig41271  HRDFYDEYMAVLDMDADFYLETMDRIFLDQHLPKGLMQFQGKAVNCTDITDVAILTIEGEKDDMISLGQT  EAALNMCDNLPKKLKKHYIQKGVGHYGIFNGSKYRKGVAPKIKGWIKKHQD | 57% identical with the polyhydroxyalkanoate depolymerase from Grimontia sp. AK16 (e-value:7e-42) |
| cyanophycin synthetase | contig01110  contig60399  contig95991  contig59880 | >contig01110  MKILATNVYVGPNVYANFPVIRHTLDLAELENWPSVKLGEDFINGLINALPTLQTHGCSYGEEGGLIRRLRDDQGTWIGHIWEHVAIELQNLAGSPVTFGKTRGTGELGCYTVVYQYQQREVGLDAGELGLKLLTHLMPAKVQQLIDAEIDPEFDFSEELHSFVRSAQRKELGPSTASLVQAAEGRGIPWLRLNDYSLVQFGHGKYQQRIQATITSQTKHIAVEISCDKQETHDLLNDLGLPVPKQIMVYSEREAIRYAKRIGYPIVVKPLNANHGRGVSINLSSDEEVALAFAQAQEVGTSRAVLLESYITGFDHRMLVVNNELVAVAKRVPGHVVGDGKSSIEQLIDKVNEDPRRGIGHEKVLTRLEMDSQALRLIEDAGHTPETVLEPGVQFFLRSTANLSTGGTAIDVTDIVHPDNRDMAIRAIRAIGLDIGGVDFLTDDIGKSYKDIGGGICECNAAPGFRMHVAPSEGESRDVAGKVIDMLFPIGSNAKIPVAAITGTNGKTTTSRMLASIMGSAGFTTGMTSTDGVYIDGHLTVKGDMTGPTSAQIVLRDPSVDFAIMETARGGIAKRGLGYNECNVGACLNVSGDHLGLDGVDTLEQLAAVKRVVVEVAKDCVVLNADDNLCLAMADFCDAKKICYVTTNSGHGLVREHIRSGGLAVVLELGINGEMITIYDNGAHMPLLWTHLIPATIEGKAMHNVQNAMFAAAMAYSFDTSLENIRQGLRIFDTSFYQAPGRLNIYDQHPFKVILDYAHNPAAFAMITDLAGRLDVAGKRRIVISVPGDRRDEDITEAAKIIAHGFDSFVCKADDNRRGRGHDEVPQLLKAALIEQGVAESAIEVVASEEDAVNFGLNQCGPADLLVVLGDAITRCWKQIIGFNSGALVESLESSAPQTSAEKLYQPVEQIFELEEGQKLVKDNRGVRIVVEHDEESD | 79% identical with the cyanophycin synthetase from Glaciecola nitratireducens FR1064 (e-value:0,0) |
|  |  | >contig60399 (partial)  TTSRLIAHMAKMTGYRVGYTTSDGVYIQNRLLMTGDCTGPSSAEFVLKDPTVNFAVLECARGGLLRAGLG  FKNCNVAVVTNVAADHLGLKGIHTIDQLARVKGV | 98% identical with the cyanophycin synthetase from Winogradskyella psychrotolerans (e-value:4e-59) |
|  |  | >contig95991 (partial)  MEESPSNKIPGFSERLKTMFPSMFSHRCSVGEPGGFFQRVEDGTWMGHIIEHIALEIQTLAGMDTGFGRTRDY | 98% identical with the cyanophycin synthetase from Polaribacter franzmannii (e-value:3e-36) |
|  |  | >contig59880 length=315 numreads=19 Translated - Frame 4  PWIRLNKYSLCQLGYGANQKRIQATVTSETSSIGVELACDKEDTKYLLEQAEIEVPRGDIISRESSLEDA  CKYVGYPLVIKPIDGNHGRGITVDIQNYEEAVVAF | 91% identical with the cyanophycin synthetase from Flavobacteriales bacterium ALC-1 (e-value:5e-61) |
