# Supplementary material for: Microbial Consortium Associated with the Antarctic Marine Ciliate Euplotes focardii: An Investigation from Genomic Sequences
Source: Microb Ecol. 2015 Feb 24;70(2):484–97. doi: 10.1007/s00248-015-0568-9 (PMC4494151; doi:10.1007/s00248-015-0568-9)
Supplement: Supplementary file 5 — (DOC 45 kb) [file 248_2015_568_MOESM5_ESM.doc]

**Table S3**: List of the contigs, and corresponding predicted amino acid sequences, resulting from the tBLASTn operation for the identifications of antifreeze proteins.

| Contigs | Predicted amino acid sequence | Antifreeze protein type |
| --- | --- | --- |
| >contig82207 length=245 numreads=8  TTCCAGGGTCACCAATCTTGAAGGCATACGTTCCAAAAGCGCGAATTTCAACCAAACCAAATCGATCATCATTCAATGTGATTGGGTTTTTGGTTCCCCATTTTTCGTCTGTAAAAAGATGTGTGTTTACGAAATAAACTTCGGCTTTAAAGGGACTGTTGAAACCATATTTCCAACCCTTCAGCGTAGTTAAGATGGGAAGATTTTGCGTATTTAAAGTATAGGTGCCGGGTTTAAAAACATCT | DVFKPGTYTLNTQNLPILTTLKGWKYGFNSPFKAEVYFVNTHLFTDEKWGTKNPITLNDDRFGLVEIRAFGTYAFKIGDPG | Bacterial type I (e-value:7e-37) |
| >contig134148 length=172 numreads=6  CCAAAACGTTCATCACTTAACATAAAAGGGTTTTTTGTTCCCCATTTTTCATCTGTAAACAAACGAGTGTTTACAAAATACACTTCAGCTTTAAACGGACTATCAAAGCCATACTTCCATCCTTTTAGCGTTGTCAAAATTGGAAGGTTCTGTGTGGTTAAATCGTACGTGC | TYDLTTQNLPILTTLKGWKYGFDSPFKAEVYFVNTRLFTDEKWGTKNPFMLSDERF | Bacterial type I 3e-22 |
| >contig120446 length=187 numreads=9  ATTCATTCACGAGGGGCAGCTTGCGGATGTATTCACTCCTGGGCTTTATCTGTTAGAAACCAACAATATGCCAGTCATGACGACCCTGCAACACTGGGACCATGGCTTTGAAAGCCCGTTTAAATCCGAGATCTATTTCGTCGACACAACCCGCTTTAGTAACCTCAAATGGGGCACGAAAAACCCA | FIHEGQLADVFTPGLYLLETNNMPVMTTLQHWDHGFESPFKSEIYFVDTTRFSNLKWGTKNP | Bacterial type I (e-value: 2e-35) |
| >contig74690 length=264 numreads=11  TCTGAAATGGGGCACCAAAAACCCGATCATCGCCCGCGACCCCGAGTTCGGACCCGTGCGTCTGCGCGCCTTTGGCACCTATTCGGTCAAAGTGTCCGATCCGGCCAAATTCCTGCTGGAAATCGTCGGCACCGATGGCGAATTCACGATGGACGAAATCAGCTTCCAGATCCGCAACATCATCGTGCAAGAATTCTCGCGCACCTTGGCTGGTGCTGGAATTCCCGTGCTGGACATGGCCGCCAACACCCGCGAACTGGGCAC | LKWGTKNPIIARDPEFGPVRLRAFGTYSVKVSDPAKFLLEIVGTDGEFTMDEISFQIRNIIVQEFSRTLAGAGIPVLDMAANTRELG | Bacterial type I (e-value: 3e-50) |
| >contig00947 length=5380 numreads=769  GCGTCGTtCCTTCTGATTTAGCGCGCAATGTGATTGCGGCACCGTGTTCATCACTACCACAAATGAAAGCGACATCATGGTCGTTCATGCGTAGAAATCGCGTAAAAATATCTGAGGGAA  GGTAGACTCCTGCTACGTGTCCTAAATGAAGGGGGCCATTTGCATACGGCAAGGCCGCGCATACGGTGTATTTTTCTTTTGACATTCTGAAATGATAGAATTTATGGGGCGAAGATAGGT  ATTATTTGGATCGCTTCGCTTTtGGAGCGTTCATTCTGAACTTTTGGATCGGTCTGCGACCTTCTGGATCGCCTTCGGCTTTTAGATATTAGAATTTAGACGACCCGGTAACGTCTAAAT  TCTAATATCTAATATCTAATATCCAAAAAAGAGTTCACGACTGATCCCAAATTCTACTTCGTAATCTGCGTTCGCACAACTTCTCCGTTACTCAGCGTTCCTACAAGGATGTAATTGCCT  GGGAGAAAATTAGATAGATCTAACGTTATTATAGTCTTAGACGGATACTCTGTTGATTGAATCAATTGTCCAGTGACAGTTAAAAGATCTACTTTTTCGATGAATTCAGAAGCGGAATAT  TCAATGTTTATTTCGTCTACAAAAGGAACGGGGTACACATTTAAATTCTTATTAAGAAGTTGGCTTTCATTCAATCCCGCAGACGTTGAATCGATTTCAATAAGGAAATAGAGACTTGAA  GGAGCATGGAGATCATTCCAAAGTCCAGTGTTATTTCCATTCAGACGCCATGTTAAGTAATTTTGATCACCACCAGAATTGTCTGGTTCAGGAGGAGTTACACCCCACGCTGAAAATGCT  CCGCCTACCGCTGCaCcACTAAAATTCCCCAACCAAAAATCTTGGCCCGAACCATCTCCAGTACCGTCCCAAATCCagTTTCCTTCCGTATACTTATCTGTTCCTCCTAACCAAATTGAA  GAAGCCCCAAAAACGTCAATTGTTCCATTTGGATTGGTCCCGGGATCTGTGGATAACCATTGAAAGATGCTATCATGCTCTAGTTGACTTTCTATTGAAGCCAAGTAACCTCCACGAGCA  ACAGCACAAACTGTAGCTTGTTCCCAAGTTAATGTCTCCTTGATAAGCTCATACGTGTGCCCGTCATAGGTGTGTGTTATTAAACTTGGAGATGCCGTGCATTGCGCTTCGGATTGAAAT  GACAATAGGAGAGCTGTTATTGGGAGAATTAATTTTAGCATAATCTTTAGTTTGAATTTAGAATATTCAGGAAGGTAGCTAAATTCGTCCGCGTATACTTTGTGAAAAGGCACGACGGTT  GAAAGGCTAATTTTTTCGAAGGTGGATCGGTCATTCTGAACTTTTGGATCGCCTGCGGCTTTTGGATCGCCTGCGGCTTTTGGAGCGCTACGCTTTTAGATATTAGAATTTAGACGACCC  TGGAACGTTTAAATTCTAACATCTATGAAGGAGTTTACGACTGATCCAAAAGGCGTTAGCCGATCCAATATCCAACCcTACTCAACCAATACTCGAATCCCCTCCGAATGCGAAGTAAAC  TCTGGCGCATACATGCATTGAATTGTAGAAATTCCATTCGAGAAATCACCGAAATGACTCACGCGAAGATCGTATTCAAAAACATACGTACCCTTGCTCAAGTATTCAATAAAGAAGTTG  GTCGCCGCGTCTTTTGTACTTTCGTAATACCCCAATCCGTCCTGATATTTGTAACGAGAGAATACATTTATAGGCTCGAATCCAGCCGCGCGCATGTCTTTTAGGTGCACGTATTCCATG  TCTCTATCACTTCGAAGTTCAATACGAACTCTGACTTTGTCACCAGGTTTCAATTGTGTTTCATCCGTTATTGGCGAAATAACTGGTCCGCTATCCGTATTTTGAACAAGGAACAATTTT  TTGCTTAATTTCAATGGCGTTTCGTGCGGTGTAATTTTATCGAGGTCTTCGAAATATTGCCAGTACATTGCTCCCCAACTTACGCCTTCAGTTGTTCGAGTCACGCTTACGTTCCCCAAT  TCAGGTTCAACTTCAGTTCCTGACCATGATGTTTTGAAATAACCCGTTCCCGCTTCAACTTTTGCGCCCAACGCCTCAGGGTCGAGCACAGAACCGTTTATTTTAATCTCCACTTGTTCA  TCGTTTGCAAGGATGTCCGTTCCGCGAAGTAAGAGCGCATAACAAGCTTCGGCTGTTGCTTTTGTGGTTTTCCAATCTGTAGTTTGCTTTTGTTTCAGTAACCAAACTTTCATGTCTTCA  ACCGATCTTTGGTCATTCGTCACTTCGTCAAACGCTTCGATTAATAAAGCTTGTGTTTCAATTGGCGCTTGGTACCAGTAATAACCGCGAACATTATCTTTCCAATACATTCCAAGTTCT  TCGTGTTGAATCGCGCGTTCCTTAATGGAAGCCATTATTCGTTCTGGAATCAAATCAATTTCATACCGTTTGGCTTGAAGCGCGATCATTCCTTCATTGTAAATGTTGAATTTCATCCAA  TACGTTGCCGCCTGATCTTGGTAATAGTCAAAAGCAGTCTTCACTTCCTTGCTCATTGGCACATCTTTGAAATAACTTCGTGCATACAAATACTGTACTTGAAGGTTTGAAATATGTTGC  TCTTTTAAGTAATTTTTATTGTGCGTTTTGACCCAATTATAGTCGTCAACAATTCGATTATCCAGATAGCGAACTCCATTTTGAACCATGTTCCAAACGGATCGATCCTCGCGAATGTCT  TTCACACCCAAGTTGTCCAAATGGCCCATTCCGGTCACAATATGTTGTGTAATGTAGCGGCTCTCTTTCATTCCCGGGAACCAAGGCCAGCCTCCATTGGAAACTTGCATTTTCTCTAGT  TTTCGAATCGCTTTGCTCAGTTGATTGTCCATTTTGTTCAGGTCGAACAACAAGGCAATTCGTTTCTTGCGCTCGCTTTCGTTTTTAGCGTCGAGCACCCAAGGCGTCTCTTCGAGCATC  AATGATTTCAATTCTTGATTCTTCTCAAGGTTCGATAAAAAAGCATCGGGACTGCTTTCTTTCCAACTTTCAAATACATTTTTGATTTTTGGACTCGAATTCACAATGTTCGAAGCCAGC  GCATTTGAATAATACCGTGTAAAAGTCTGTTCAGCACATTCGTACGGATACTCCATCATGTATGGAATCGCTTGAACGGCATACCATGCTGGATTCGAAGTATATTCCAATGTCAATTTA  TGGTGTTTAATGCTTTCAGAACCTCCGCTGTTCACCAATTTATCGAAAGTGAAATTTTTCGTTCCGATTCCTCGAGATGGCAATGGCATGGATTCCGTCACTAACATTCTGTTGGATAAG  ATTGGAATGGCCATTTCTTCACCATCCGTATAATTTCCAGATTTTGCTACAACTCGATACGTCACTGCTCCTATCCCAAATGGAATTTCAATGTTCCATGAAACCGCATCACTTtGGCCT  TTTTTCGCAGAGAAGCTAACGATAGAATTGTCCATATTGAACAATGAATCGATTGGTTTCATTGACATTGCATCAAAAAGCATTAGTTGCGCGGAACCGTTCAAGTCTTCTTCTGCCAAG  TTGGAAATTTTGGCTGTGAAAGTCATTTTATCTCCCTCTCGAAAGAAACGCGGCGCATTTGGAACAACCATCAGCTCTTTTTGCGTTACGATTTCTTCTTGGATATACCCGATTTTCAAA  TCTTGAGTATGGGCCATTCCTAGAAACTTCCATTTCGTTAAGGCTTCTGGAATTGTAAACTTGATAATGACCGCTCCATCCGCATCCGTTTCCAATTGAGGATAGAAAAAAGCTGTTTCA  TTCATGTTCGTCCGAGCTTTTATTGCTCCCAAATTGCCCTGTTCTAGTTCTCCGCCAAAGTCACCTCCAGAGGGCATGACACTGGATTCTTTGAACCTTTGGTCTTTGTCCATTTTATAG  CCAACCGTCATCGTCTGTCGATTCGCACTTTGTCCTTCTGCACGGCTTTCAGATTTGGGTGGAGCTGCCATTGGTGAATCCATAACTTCTTCTTCGGCCCATTCATAAACATTTCCAGTA  GAATTTGATGAGTAATTGAATGAATCAAAGCCGTTATAGTTGCCATATCTATACGTATTATAACCCCACCAATTCAATTGATCGTACGTTCGAGATGGAGAAGATAAGTACTCGTTCCAA  TCGTTTCGATGCAGCTGTGATTGCCTAGAAGTAAAGCAGTTGCTGTTCCAGTTTTTCTCTGAATAATTAAAATTGTAAACGCTCATGTAAATGGAGTTTGCAGCAAACGCATCGAGTGAG  GCGTCGTACATTGTAGCCAACATCTCGGCCGCAACTTTCTCTCCTTTCGGTCCTGTAATCTTTACTTTCCATTCTTCTTTCGATCCCGGCAGAAGTTTGTTTCTAAACGTTTCAAATTCC  AATTTGAGATCCTTATTGGAGTAGGGAACATAGATGGCACTTTTTGAGGTGTGCGATCGGCCGTACTTCACCGTGGTAAAATGAACCATAATATTACCGCGGTACTTCTCTAGAATTGGA  ATTGATATTTTTTGCTGAGAAGAACTTAAGGTGATTAATTtCTTGCTGATGATTTTTCCTTTGTGCTCAATTTCATACAAAACCGAAAGGTCTTTCGCTGCAGAATAAATAATGAATTCC  GCATTTTCTCCCGGTTCACAAAAAACGTTCAATGGAACAAATGACCAGATATCATTGGTAGGGTTCGTTTTCTCAAATTTATCAATCAATCGAATGTACTGCGTTTCTGTAACTGTTTCT  CCAAACGCATCTTTGGACTCAATGTCGATAACGTACCTTCCTGGTTTCCAAGTTTTCAAAGATGTAAAATCAACCGTGTCTCTTTTATCAGTGTCAAATGAAGTACTGTAAATTTGATTG  CCTCTTTTAAAGTCTTTCACTTCTAATTTTTCGTCGTATGCATCGTGAGGAAAGAGCTTTTGATATTCTGATTTTGTAAATTTTTGAATGTCTGGTTTTTCCCAAAGCGATGTTCTAAAA  AGTCCTTCAGGCTCAATCAATCGTGTAATTTTCaCAGTTCCTTTTGCTCCAACATCTTGACCGTTTAAATTGGTTGTACGAATTGGAAACTGGTTGTTTCCGTTTCGTTCTAATGTTTCG  GAAATCGGCATTGAAACATTCATGCAATTGTACCCcACTCGAACCCATTGAGATGAACTGTGCGTTTCGCCGTTAACATCGGTAACGTCTGCCGTTATTTGATAGGAATACGTTGGGTAG  AATGTTTTTTTAATAGAAGGATCTTCCTTGGCTTTAAATTCAATGATAAATTcACCGTTCTCATCGGTTTTTGTTTCTCCATGTGCCACTTCAATGGCAG | MLKLILPITALLLSFQSEAQCTASPSLITHTYDGHTYELIKETLTWEQATVCAVARGGYLASIESQLEHDSIFQWLSTDPGTNPNGTIDVFGASSIWLGGTDKYTEGNWIWDGTGDGSGQDFWLGNFSGAAVGGAFSAWGVTPPEPDNSGGDQNYLTWRLNGNNTGLWNDLHAPSSLYFLIEIDSTSAGLNESQLLNKNLNVYPVPFVDEINIEYSASEFIEKVDLLTVTGQLIQSTEYPSKTIITLDLSNFLPGNYILVGTLSNGEVVRTQITK | type II AFP (e-value: 4e-05 ) |
| >contig118102 length=189 numreads=15 AACCAAATATGATCCTTCTTCAATGGCATAAAAGTAAACTCCTGACTGCACCTCTAAATTTTCTATAGTAAGCTCATTTTCAAACTCACGAGACACGATTTTACTGCCATAAATATCATA AAGCGTAAATACTGCAGTACTAGATTTATCCGATGTTACTTGtATCGTATTCGTAAATGGATTTGGATA | YPNPFTNTIQVTSDKSSTAVFTLYDIYGSKIVSREFENELTIENLEVQSGVYFYAIEEGSYLV | ice-binding protein (e-value: 0.009) |
| >contig03294 length=3221 numreads=477  ACGAAATGGGCTTTTTTTGTGGTTGCTTGGTTTGTCAATTATTACTCGTTTTTCATTCTAAGTTACAAGCAATACTCCATTCAAATTCCAAACACGCATACCAGAAATGACGCTGTCAAAATAGTATCTCAGTCTAAAACTCCTAACCGTTATTTAGTATTAGACAGATAATTAGAATCCCAATAAGTGTGAACTATGTAAGTCGTAGTAATAGTTGTGTAACACATTCTGAAACAATAGTTCGCAACTTTGATTCGTGAAAACTATTTCACAATTGTTGCGCCAAAAGCGCAAATAAAAAAAAGGGAAATGAAAAAAATAAAAATAACAATGTTAACCGCAACTGTTCTATTCGGACTTTTAACTGTGGTTGGGTGCAAAAAAGAAAAAAATGATCCCACAACACCTGGAACAACTACAACTGTAATCCCTCTGCAAACAACTGTACAGACGCCGATAACTTTGGGTTCAGCCAATAATTTTGCAGTAATCGCTGGGTCATCCGTTACGAACACTGGCGCAACAAACATTACAGGAGATCTTGGTTTAAGTCCGGGAACATCAATTGGTGGATTTCCTCCTGGAATTTTAAATGGTACCCTTCACATAAACGACGCCATTGCCAATCAGGCAAAACTTGATATTACAACGGCCTACAATGATGCTGCGGCAAGAGTTGCAAGTGATATGGTAACAATTTCGGGGAACATTGGTGGTTTGACTTTAACGCCCGGGCTATACAAATCGACATCATCGCTTGCCGTTTCTTCGGGCGATGTTACGTTCGATGCGTTGGGAGATCCAAGTGCTATTTTTGTAATACAAATCGCATCAACGCTTACAACAACACCAGGGCGAAAAGTACTATTAAGTGGTGGAGCACTTGCCTCTAATATTTACTGGCAAGTGAGCAGTTCAGCATCTTTTGGAACGACTACGTCTTTTAAAGGAACCGTAATTGCTTTGGAATCCATCACTTTCGATACCGGAGCTACACTGGAAGGAAGGGCACTTGCTAGAAATGGAGCTGTCACAATGGAAGGAAATACATTTGTACTTCCTTAAATAGCATCTTACGTTTTAATCCTATTAGGCCTTACTCAACATTATGAGTAAGGTCTTTTTGTTTGTCGATTCTACCGTGAACCAAAACACAAAAATGTGAGAACTATGTAACTATGGAGAGAGATTGTATAACATAAGATGAAGTATAAAACCTCATCTTGAAGTGTGAAAATGATTTCACACAACTAAATAAAACAAAATGAAAACACAATTACTTCACCTACTACTCATTGTCTTTCCGCTATGTGCGCCACAATTAGTATTTGGCCAAGCACCAAATTTAGGCACAGCTTCAAATTTTGTACTCTTTTCAACAAATGGAGCCGTTAGTAATTCAGGTATTTCACATATAACAGGTGAAGTTGGAACAAATAACGGATCAAGTACTTCATTCGGAAACGTTGATGGTTCTATGCACGACGGAGACGCCGCAAGTATCCAATGTGCAGCAGATTTATTAATCGCTTACAACGAACTAAATGCTGTTATTCCTGGATTCTTTCCTGCCCCTTTACTTGGAAATGGTCAAACACTAAACGCGGGTACCTATTCTATACCGGGTGCCTCAACGTTAAACCTAAATCTAAATTTGGATGCCCAAGGAGATCCAAACGCTGTGTTTATTTTTCAAATTCAAGGCCCCTTATCAACCAATGCTGATTCCAAAGTTAAACTGTTAAATGGAGCTTTGGCTTGCAATGTTTTCTGGAAAGTGGAAGGATTGGTAAGTATGGCTTCTGGCAGCACAATGAGAGGAACTATCATTGCGAATAATGCCGCAATTGAAATGAATACGGGTGACACTCTTGAAGGCAGAGCACTTTCTACAGCGGGAGCAATTACAGTTGATGGAATATTGGCATACACACCAATCGGTTGTGGAAGTCCTGTACTTGACGGCCCAATAGCTCCTACTCTTGGAGCGGCTGCCTGTTATGCAATATTCTCTACTGACGGTGCCGTAACAAATACGGGCACGACAACAATTACTGGAGATGTAGGGTCTAATAGTGGTTCTCCAACAGGCTTCGATCCTTTGCTTGTCACAGGTGAAATTCACTTGATTCCAGATGGCTCAACTGCACAATGTGCGAATGATTTACTCGTTGCCTATAATTATATAAATACGTTGCCCTATGATATCGAACTGTTGTATCCTGCACAATTTGGAAAAAATCTAGTTCTTACCCCTCACACCTATTTAATGGGAGGTGCGGCAACATTTACTGATTCTCTCTACTTGAATGCTCAAGGAAATCCTGATGCCGTTTTTGTTATTCAGATTAATGGAGCTCTTTCTACGAGTACTTATTCAAAAGTACTATTGATAAACGGAGCGCAATCGAAAAATGTGTATTGGAAAGTTGAAGGAGCTGTAAGCATTAACGATTATTCAGTATTCTGCGGAACTATTATCTGTAATAATGGTGCCTTGGGCGCAATAAATACTGGAGTAACTCTAAATGGACGAGCACTTACAACTGCAGGCGCACTAAATACTTTTTCAATTGACGCAATTGCTCCA  AATTTACCCTTGAACTGCGAGTCTGTAGGAGTCTCAACTATTGAGATTACAGATGAAGTAATGGCTATTTATCCGAATCCCTTCAATCAAATGACGACCATTTCGATTCATGATGCTTCTGAGAGTAATAGTTATGTTTTAGAAATCTACAATGCGATGGGAGAGCAAATGATCAATACAATCATTACTAATCCGTCAACGCCTCTTGATTTTACAGACTTTAATTCAGGAATGTTTTTCTACAAGGTATTCAGTAATCAACAGGTCATTCAAACAGGTAAATTGATTGCCCAATAAATTAATGTCTAGTCCTAAATAACCGTTACAGGTTTTAGACTAAGAAACTTTATTTATTAAATAGCGAAGAGAACGTTCTCTTCGCTGTTTTTTCTTTTCTGGAATCCTTTAATTCGCACACAAAATAAAATTGGCAGACATGAAAAAGAGAATTCCTATCTATCATTTTATCGCTTTTGGGCTTCTCGTAAAATCTTTATGATTAATCTGGCGAATAGCCCAAAGTGTGAATCATGTAAGTAGTAGGAATAGTAGTGTAACAGATAGCTAACTAGGTATTCTCACCTTTGTTGTGTATTACATAAAACACTAAAAATGAAAAAAGTAAATCTAT | ORF:1265..2887 Frame +2  **MKTQLLHLLLIVFPLCAPQLVFG**QAPNLGTASNFVLFSTNGAVSNSGISHITGEVGTNNGSSTSFGNVDGSMHDGDAASIQCAADLLIAYNELNAVIPGFFPAPLLGNGQTLNAGTYSIPGASTLNLNLNLDAQGDPNAVFIFQIQGPLSTNADSKVKLLNGALACNVFWKVEGLVSMASGSTMRGTIIANNAAIEMNTGDTLEGRALSTAGAITVDGILAYTPIGCGSPVLDGPIAPTLGAAACYAIFSTDGAVTNTGTTTITGDVGSNSGSPTGFDPLLVTGEIHLIPDGSTAQCANDLLVAYNYINTLPYDIELLYPAQFGKNLVLTPHTYLMGGAATFTDSLYLNAQGNPDAVFVIQINGALSTSTYSKVLLINGAQSKNVYWKVEGAVSINDYSVFCGTIICNNGALGAINTGVTLNGRALTTAGALNTFSIDAIAPNLPLNCESVGVSTIEITDEVMAIYPNPFNQMTTISIHDASESNSYVLE  IYNAMGEQMINTIITNPSTPLDFTDFNSGMFFYKVFSNQQVIQTGKLIAQ*  ORF:310..1065 Frame +1  **MKKIKITMLTATVLFGLLTVVGCK**KEKNDPTTPGTTTTVIPLQTTVQTPITLGSANNFAVIAGSSVTNTGATNITGDLGLSPGTSIGGFPPGILNGTLHINDAIANQAKLDITTAYNDAAARVASDMVTISGNIGGLTLTPGLYKSTSSLAVSSGDVTFDALGDPSAIFVIQIASTLTTTPGRKVLLSGGALASNIYWQVSSSASFGTTTSFKGTVIALESITFDTGATLEGRALARNGAVTMEGNTFVLP* | Sea ice organisms AFPs *Flavobacteriaceae bacterium* strain 3519-10 (e-value: 4e-75), *Stigmatella aurantiaca (*1e-179) |
