# Supplementary material for: Microbial Consortium Associated with the Antarctic Marine Ciliate Euplotes focardii: An Investigation from Genomic Sequences
Source: Microb Ecol. 2015 Feb 24;70(2):484–97. doi: 10.1007/s00248-015-0568-9 (PMC4494151; doi:10.1007/s00248-015-0568-9)
Supplement: Supplementary file 6 — (DOC 44 kb) [file 248_2015_568_MOESM6_ESM.doc]

Table S4: List of the contigs, and corresponding predicted amino acid sequences, resulting from the tBLASTn operation for the identifications of members of the transposase and integrase superfamilies.

| Family | contigs | predicted amino acid sequence | Blastp results |
| --- | --- | --- | --- |
| Transposase DDE | contig27723  contig32933 | >contig27723  MDAPFGSWGTQTFIAGLSADALLAPWVIKGAMDGEAFAAYVEQVLVPELEPGTVVILDNLATHKNAAAAKAMREAGCWFLFLPPYSPDLNPIEMAFSKLKAHLRRIGARTFTDMFHALTEICDLFSPEECWNYFKAAGYVSG | 93% identical to the DDE transposase from Rhodobacteraceae bacterium KLH11 (e-value: 7e-99) |
|  |  | >contig32933 MDNCTSHQAIRTRKFIGKLNVEVVYFPPYSPIFAPIEEVFSFLKYNIIKNNKKSALNLKETRRPHRDK | 36% identical to the DDE transposase from Methylobacterium extorquens (e-value: 3e-07) |
|  |  |  |  |
|  |  |  |  |
| Integrase rve | contig40952  contig66476  contig64503  contig17141  contig12461  contig16419  contig27222  contig12322  contig10561  contig16251 | >contig40952 (partial)  SIGAQCQLLSIPRSTFYHRPSGESAENLALMALIDRQFMETPFYGVRQMTWHLRNEGHAVNPKRVRRLMRLMGLMPIYRRPNTSKPAKGRQPYPYLLQGLTIDRPGQVWCSDITYLPMRRGFLYLVAIMDWHSRRVLSWRLSNTLEA | 76% identical to the rve integrase from Rhodobacter sp. SW2 (e-value: 8e-72) |
|  |  | >contig66476 (partial)  VEALNEAIHKFGLPEIMNTDQGSQFTSFAWTDRLRRTGVRISMDGKGRFLDNIFIERLWRTLKYECVYLH  AWETGSEAKAGIRKWMTFYNHQRPH | 98% identical to the rve integrase from Citreicella sp. 357 (e-value: 5e-63) |
|  |  | >contig64503 (partial)  EFCVEALNEAIRLHGPPEIMNTDQGSQFTAFVWTDRLRRAGIRISMDGKGRFLDNIFIERLWRSLKYECVYLRAWETGSEARTGLRTWFDFYNHRRPHA | 81% identical to the rve integrase from Citreicella sp. 357 (e-value: 3e-55) |
|  |  | >contig17141  MLHAEGAVIGRFKVRSLMREAGLISKQPGPHRYKHATVERIDIPNRLNREFNVSGPDEVWCGDITFIWAQGRWHYLAVVLDLYRRRVVGWALSTQPNANLVVAALDMAYMQRGQPSGVMFHSDQGSQYASRLFRQRLWRYRMEQSMSRRGNCWDNSPMERVFRSLKTEWVPSVGYSTMREAKQDISHYLITYYNWVRPHRYSAGLAPAVAEEKLNSLSGNS | 56% identical to the rve integrase from Pseudomonas amygdali (e-value: 5e-133) |
|  |  | >contig12461  MCRVLKVHRSGYYAWKLKPLSNRAIEDAALLVEIKRSYEDSYGIYGSPRIHYDLREAGIVCSENRVAKIMRNAKLKSIRGYRKPRYKSGRPSVASPNRLGQVFTVSQPDLAWVTDITYIRTYQGWLYLAVVIDLYSRSVVGWSMKPTMATEIVLDALTMAVWRRKPKQPVIIHSDQGSQFGSDDFMRWCKDNRLEPSMSRRGNCYDNAVAESFFSSLKKEHIKRKIYVSREEAKSEIFEYIEVFYNRKRRHSHLNQLSPMMFEQLQNGN | 96% identical to the rve integrase Methylotenera mobilis JLW8 (e-value: 0.0) |
|  |  | >contig16419  MATVSCTGTSGKYRFIEKLRAEVKVVKLLKFFDVSKSGYYDWRERQPSARAIEDVELKKEILEIFTENKGRYGSPRIFKALQKQGHNISKKRVERLYRALGLVARVMRVTTRSAFYKRFLTTGKNLRPNGEVPLAKDKVWVADVTYLKVNGVWRYLSVIMDLYSRRIISWSLDKNRTMEVTTRTLKNALKKRKPQGDLMLHTDRGVEYRGNIYQTALKRGNITHSLSRAGKCTDNAHMESFFHSMKVEAIRGNVFKSDKELRGTLGSYINKYYNVSRLHSGINYCSPIEYEALAA | 89% identical to the rve integrase Colwellia psychrerythraea 34H (e-value: 0.0) |
|  |  | >contig27222  MKQHKLKYPVEMMSKILDVSTSGYYNWLKSGPSDRWLENRKIIELIEDIFEESHQSYGSPRMAVELEKRGYKVSRPRAARMMKALGVEARRTRKFKNTTDSNHNYPVSPNLLNQNFSVKRRNQVWVSDITYIETTNGWVYLTVIIDLFDRKVIGWSLSEDMTAKNTVVKAWYAAVEN | 64% identical to the rve integrase Galbibacter marinus (e-value: 4e-78) |
|  |  | >contig12322  MKYQFIHLYRKEYPIVRMCDVLDVSASGFYDWVDRPESFRGLESRR  LSQKIATIHKKSRCIYGSPKIHKELIEDNEYCSVNRVARLMKKADIQSKLARKFVITTNSKNTMKAAPDLLQRQFTTKQPDIAWVSDTTFIATREGWLYLAVILDLFSRQVIGWSMSERNDAQLVQDALTMSIWKRGTGRNVIVHSDQGSTYASGAYQQQLASSQLICSMSRKGECLDNASC | 67% identical to the rve integrase Thioalkalivibrio thiocyanodenitrificans (e-value: 8e-104) |
|  |  | >contig10561  MATVSGGSPSERFGFIEKHRETLGVKYLCSWLEVSRSGFYAWRHRPIAKRIINDAQLLISIKHVFDKNHQTYGSPRVFHALKRSGIVTSEKRVARLMQEHGLRARALKTYSKPAKVKFFYKEIKNNRKDINKADAINQQWSGDITYLKVGSRWYYLAVVLDLFSRRVIGWAFGKNKSTKLTLKALKLAINKRKPTQPIIFHTDRGAEYRAHVVQQFLVKHNITASMNRPGCCTDNAEVESFFHSLKADLIRGRLFETTDKLHSKLKDYMNYFYNRQRLHSSLGYKTP  AEFELAVN | 88% identical to the rve integrase Colwellia psychrerythraea 34H (e-value: 0.0) |
|  |  | >contig16251  LSLKLQVVQEIERGELSTTGAVRKYGIQARSTVVSWLRKYGNFDWENQTPSNMPKTPAQKLLELEQRVRLLEKQKAHLEYQVERADKKAIIFDMIIDMAEKEYNIPIRKNSSPEQSISSKKNNKKAVSRQVYYRSLRSRYKRKQKAQHVVDLVKEIRIQMPRVGTRKLYHLLQDDLRVLGVGRDRLFSILKANNMQIIPKRSYHITTDSHHRFRKHKNSVAELSIVRPEQVWVSDITYIGNRTNPIYLALVTDAYSKKVVGYDVSNTLCTTGSIRALSMAIKSRLYNKEELIHHSDRGVQYCSDDYQKLLNRKKIKCSMTESYDPYANAVAERINGILKQEFLGYDRKLPINIMKQLVKDSVRIYNAIRPHYSCHMLTPVQMHNQRNIQMKTYKRKNSEENVFFAV | 58% identical to the rve integrase Solitalea canadensis DSM 3403 (e-value: 4e-134) |
| Tyrosine recombinase | contig00297 | >contig00297  MTDTAIVSLDNQDHIDLFIDAMWLESGLSKNTLSAYRSDLNRFAKFIATKSLIMVDQSDVQKFLGLMMAEGTKASSSARVLSTLRRFYRYQIRQNRLSKDPCVQVLSPKQGRPLPKAMSEQQVESLLAAPDLTTSLGIRDRAMLETLYATGLRVSELVELTLLEISLQVGVVRIVGKGNKERLVPLGEQAID  WIERYQQGARLDILKQRQSDAMFVTARGSSMTRHAFWHIVKKHALTAGIQQSMSPHTLRHAFATHLINHGADLRSVQMLLGHADLSTTQIYTHIARERLQSMHAKHHPRG | 62% identical to the tyrosine recombinase XerD from Methylobacter marinus (e-value: 3e-129) |
| Gene Tranfer Activity (GTA) | contig01344 | >contig01344 (partial)  EGARLSGGRPMSAEDGTPMSRVYGYARVTTTVIWATRLEEKKTTKRQGGKGSVTGGPKVTSYSYFASAAFGICEGKIAGIKRIWADGKEVDVRNIEYRFYNGDDDQLPDALIEAKQGVGNAPAFRGTAYLVIERMPLERYGNRLPQIQVEVIRPVGQLEKDIHAVSIIPGATEHGLNPSPVLTRINQRETQELNRHILHADTDWQASIDELQAVCPNLKHVSLVLAWYADDLRASHSRLRPGIVSRDSLQETKLWEVSGLSRQSTDAHLISQADGRSAFGGTPSDSSVIAAIADLKTRGLSVTLNPFILIDVPTDNVLPDPYSDGAQSSYPWRGRITPHPAVGRIGSVDGTVDAEAQITNFIGGSEVNDFYISDGLIKNNSGDFGYRRMILHYAKLAELAGGVDTIIIGSEMRALTTARGATNTFPFVENLADLASDAAQIVSPNTKLIYGADWSEYFGYHPQDGSGDVFFHLDELWASPDIAAIGIDNYMPLSDWRASDAEAGHTNPDGMRWGSDTNALAQAITSGEGFDWYYASHADRQTRIRTPITDGAFSKPWTYRYKDLQSWWANQHYERIGGVEKVSPTNWIPQSKPIFMMELGCPAIDCGANQPNTFIDKNSSEAKLPHFSNGSRDDDIQRSFLSAHHNYWASGAVANPLSSLTSKQMVDPERLYVWAWDARPLPAFPLNVGVWGDGENWLRGHWMNGRLGTAVAADLVQSIFADHGLPAPKTETLAGCVTGMILSTPASVRATIEPMLKLFGGVASDADGLCFSGVDAYGAHIIERDNLVLVGNDPRIERTLSQVNELPNEVQIGFRDPLQDYQARTSYSRRRENTVDRQIVLDVPAILDADEARKFADQILSNAWSGRETIKFGLPLTNCELNTGDVIAFGDAPDQHFIIEQITLSEFMAVEARSISKPRRVSVLPTLPGVQESAAFFGTFGGPPEITILELPLINGASENEQLRVASLSSPERTQNIFASATSQGFDLRGVIEQNATIGTLATSLLPAVAGRFDYANKIDVELQNGELESLDTLSLFTGRNLAAVEVATGQWEVFQFANAEEISSDTWRLSMLLRGQGGTEDLMSLGADIGAKFILLDDAVMPIGLRDSEIGSLLNFRIGEAGKDFSDRYYTALSAEGGIRAFKPLSPVHIRAVNVEDDAILLKWVRRGRINADSWLGLDIPLGEAFERYKIDVLDESQTTLGSFEASQPDFLFTSAMLNDLFGGQPATLSVRVAQLSERVGEGIPAFA | 81% identical to the GTA protein from *Ahrensia kielensis* (e-value: 3e-55) |
|  |  |  |  |
