# Supplementary material for: Microbial Consortium Associated with the Antarctic Marine Ciliate Euplotes focardii: An Investigation from Genomic Sequences
Source: Microb Ecol. 2015 Feb 24;70(2):484–97. doi: 10.1007/s00248-015-0568-9 (PMC4494151; doi:10.1007/s00248-015-0568-9)
Supplement: Supplementary file 7 — (PDF 422 kb) [file 248_2015_568_MOESM7_ESM.pdf]

Table S5: gene annotation of the contigs that showed a high similarity to sequences from representatives of the genus Francisella.

<table>
<tr><th>Contigs</th><th>Blastx</th><th>Max score</th><th>Score</th><th>Query Cover</th><th>e-value</th><th>Identity</th><th>Blastn</th><th>Score</th><th>Query Cover</th><th>e-value</th><th>Identity</th></tr>
<tr><td>>contig50994 length=360 numreads=13
CCAGTAGGTAACGAGCTTttAGGAAGAGTTGTTGACGCTCTTGGTAACCCAATTG
ATGGT
AAAGGTGAAGTTGCAACAGATCTTACATCTCCTATTGAAAAAATtGCACCTGGTG
TGATT
TGGAGAAAATCAGTTGATCAAGCTTTACAAACAGGTATTAAGTCAATCGATTCA
ATGGTT
CCAATTGGTCGTGGACAGCGTGaacTTATtATTGGTGATAGACAGATtGgTAAAAC
AGCA
ATCGCTGTAGACACAATCATTAACCAAAAAGATTCTGGTGTGAAATGTGTTTATG
TTGCT
ATAGGTCAAAAAGCTTCAACAATTGCAAACATTGTAAGACAGTTAGAAGAGCAT
GATGCT</td><td>ATP synthase F0F1 subunit alpha [Francisella tularensis subsp. tularensis SCHU S4]</td><td>229</td><td>229</td><td>100%</td><td>6,00E-70</td><td>95%</td><td>Francisella cantonensis strain 08HL01032 ATP synthase F1 alpha subunit (AtpA) gene, partial cds</td><td>292</td><td>94%</td><td>1,00E-75</td><td>82%</td></tr>
<tr><td>>contig45579 length=399 numreads=28
CTGTAGTTACAGGCCATAAAGAACCTGTACGCACCCAATTGATAAGTGCATCAG
CACTTG
CTGTTATAAAACCTTTATTCTCGTTACCTATTCCCACTCTAAAGCTCCTTTTTTCCA
AGC
ATATATTAgaCCTAAAAACAGCACCACCAAAAATATAATCATCGCAAAAAACGCA
TGATT
AGATATAGCTGGCACTGTTGCACCAGCTCTAAGGTTTATGCCCCAGGGAATAAT
AAATGC
CAGCTCCAAGTCAAATAtcAAAAATAACACTGCTATTAAATAAAATCGTACATCA
AGCTT
CTCTCTTGCATCACCAAATGCAGGgAaGCCACACTCAAACGTCTCTCCTTTTGTCT
TACT
GGGATTGTTAGCCCcAACTATTACAGATAAAACTTTACC</td><td>NADH dehydrogenase I subunit A [Francisella tularensis subsp. tularensis SCHU S4]</td><td>172</td><td>172</td><td>68%</td><td>7,00E-52</td><td>93%</td><td>Francisella cf. novicida 3523, complete genome 401</td><td>401</td><td>98%</td><td>2,00E-108</td><td>85%</td></tr>
<tr><td>>contig109648 length=200 numreads=30
CTGTCCTCGAATTTCTCCTGAGTTAGAACTTCAAATAGTCAAGGGTGGTATTTCA
AGGTC
GACTCCAACAAATCTAGCGACTTGTCTTCATAGTCTCCCACCTATCCTACACATAA
ATAT
TCAAAGTCCAGTGCAAAGCTGTAGTAAAGGTTCACGGGgTCTTTCCGTCTAACcG
CGGGT
ACACAGCATCTTCACTGCGA</td><td></td><td></td><td></td><td></td><td></td><td></td><td>Francisella cf. novicida 3523, ribosomal rRNA</td><td>917</td><td>88%</td><td>9,00E-80</td><td>94%</td></tr>
<tr><td>>contig118995 length=188 numreads=8
GTCAATCcATTTACCGTATAAGATACCATTGTTATAGCTTGCTAGACATGCGATAT
AAAT
TTTAGGCTCGTAGTTGTTAGTCATTTTATCTTCCTTTtGTTTTACTGTTACAACAAC
TTG
AAGGACGCCAATTACAAAACATATTAAATCAAGGTGGAACTCACAGGCAAAACC
TTGATT
TAATATGG</td><td>antirestriction protein [Francisella philomiragia subsp. philomiragia ATCC 25017] >ref|WP_012281159.1| antirestriction protein [Francisella philomiragia] >gb|ABZ88118.1| putative antirestriction protein [Francisella philomiragia subsp. philomiragia ATCC 25017]</td><td>62.0</td><td>62.0</td><td>33%</td><td>4,00E-10</td><td>93%</td><td>Francisella cf. novicida 3523, complete genome</td><td>261</td><td>74%</td><td>2,00E-66</td><td>92%</td></tr>
<tr><td>>contig84610 length=241 numreads=8
AaaGCCATTTTATGAGAAGAAGAAATAAAAAGTTTTTTAGAAAAAAAAGAGCAA
TGGTTG
AGATCAATGTAGTACCATACATTGATGTAATGCTTGTATTACTAGTTATATTTATG
ATTA
CCACGCCAATTTTAACACAGGGTGTAAAAGTTGATTTGCCGAAAGCAACATCAG
AAAAAA
TCCCATCAAATGATAGTAAACCAATAGTTGTTACAGTCAATAAAGATGGTCAATA
TTTTA
T</td><td>TolR protein [Francisella novicida U112] >ref|YP_005825267.1| hypothetical protein [Francisella cf. novicida Fx1] >ref|WP_003035506.1| biopolymer transporter TolR [Francisella novicida] >gb|ABK89259.1| group A colicin translocation; tolR protein [Francisella novicida U112] >gb|EDN37182.1| hypothetical protein FTDG_01591 [Francisella novicida GA99-3548] >gb|AEB27307.1| Tol biopolymer transport system, TolR protein [Francisella cf. novicida Fx1]</td><td>119</td><td>119</td><td>59%</td><td>7,00E-32</td><td>95%</td><td>Francisella cf. novicida 3523, complete genome</td><td>213</td><td>96%</td><td>6,00E-52</td><td>83%</td></tr>
<tr><td>>contig90166 length=230 numreads=11
AAGTACAGTTGCATAAGCCTTTTTAAACATTGCAGCATTCACAACTTCAGCTTGA
ATTGT
AGCAATTTCTTCATTACTTGGCCAAATATCTGCAAGCATTACATCATTGCCTTCAG
CATC
AACACCTATAGCATCTTCTACTGGATCAAAATCAACTGTCCCAGCTAATGCATAA
GCAAC
CACATGAATAGGAGATGCAAGATAGTTAGCTTTTACATGAGGGTTAATAC</td><td>aconitate hydratase [Francisella noatunensis subsp. orientalis LADL--07-285A] >gb|AHB98943.1| aconitate hydratase [Francisella noatunensis subsp. orientalis LADL--07-285A]</td><td>138</td><td>138</td><td>67%</td><td>2,00E-35</td><td>86%</td><td>Francisella cf. novicida 3523, complete genome</td><td>198</td><td>68%</td><td>2,00E-47</td><td>82%</td></tr>
</table>

>contig157498 length=151 numreads=9<br>GCATTAAACTCTAATATATCAAAAGATATACTCCTTAAACAACTACACCTCTAAACTTTT<br>ACTAACAATCTCAAAGACATTCTTGCTAGGATTTGAAGCTTTGATTTTCTCAAGAGCATT<br>TTTCATCATAGCTTGTCTATCAGAGTCATAG	aminopeptidase [Francisella novicida] >gb|EDN35376.1| aminopeptidase N [Francisella novicida GA99-3549]	70.5	70.5	55%	3,00E-12	97%	Francisella cf. novicida 3523, complete genome	158	54%	2,00E-35	94%
>contig69126 length=281 numreads=13<br>CTGCCGCTGAAGCAAATGAAGAAATGATGGAAAAGTATCTTGAAGGCGAAGAGCTTACAG<br>AAGATGAAATTCATGAAGGTCTGCGTCAGCGTGTGATCAATAaCGAAATCGTTCTTGCTT<br>TCTGTGGTTCTGCGTTTAAGAATAAAGGTGTTCAGGCTGTACTTGATGGTGTTATCCGTT<br>ATTTACCTGCTCCAAACCAAGTTCCTGCAATTAACTGTGAAACTGAAGATGGTGAGCCAG<br>cTtCAAGAAAGTCATCTGATGATGAGCCGTTCTCAGCTTTA	elongation factor G [Francisella tularensis subsp. holarctica FTNF002-00] >ref|YP_007008412.1| elongation factor G [Francisella tularensis subsp. holarctica F92] >ref|WP_010030786.1| elongation factor G [Francisella tularensis] >sp|A7N9S4.1|EFG_FRATF RecName: Full=Elongation factor G; Short=EF-G >gb|ABU60727.1| translation elongation factor G [Francisella tularensis subsp. holarctica FTNF002-00] >gb|AFX69949.1| elongation factor G [Francisella tularensis subsp. holarctica F92]	143	143	63%	2,00E-37	88%	Francisella cf. novicida 3523, complete genome	209	66%	1,00E-50	83%
>contig135655 length=171 numreads=6<br>TAGAGTACCAGCATATTCGCCTTTTTCAGTAGCGGAGCATACATTGGCGCTTTTATTATG<br>CTTAAACCGTAAAATACACAAAGCTTACAACAGAGTTCGTGAAAGTAATTTTAATATCGA<br>AGGTTTAGAAGGATTTGATATTCATCAAAAGACTATCGGAATTGTTGGTTT	D-lactate dehydrogenase [Francisella cf. novicida 3523] >ref|WP_014549102.1| D-lactate dehydrogenase [Francisella novicida] >gb|AEE27132.1| D-lactate dehydrogenase [Francisella cf. novicida 3523]	109	109	65%	7,00E-27	96%	Francisella cf. novicida 3523, complete genome	222	73%	1,00E-54	90%
>contig106755 length=203 numreads=8<br>GCTTTAGTTAAAGAAGCTTTAAATGCATCAGCATCATCAGAAGCCATTTTTTGAACTTGG<br>TTCATAAGGTCTTGGTATTGCTTCATAGCTTTTTCAAGATCAGCCTGaGCTTGAGCTTTA<br>TCAGTGTTTTCAGCTTTATCTGCAGTAGCAGCATCTTTTTGATCaCcTTTGCATCGTCA<br>TTAGCAACATCATCTTTCTCTTC	outer hypothetical protein [Francisella philomiragia subsp. philomiragia ATCC 25017] >ref|WP_012280140.1| membrane protein [Francisella philomiragia] >gb|ABZ86928.1| conserved outer membrane protein of unknown function [Francisella philomiragia subsp. philomiragia ATCC 25017]	100	100	66%	6,00E-24	81%	Francisella cf. novicida 3523, complete genome	163	65%	8,00E-37	82%
>contig182118 length=129 numreads=6<br>TCGAGACCTTAATtCTCCTGTACCTTGAGCACCATTAAACTTAGACATATCGTTGATCTT<br>ACTCTTCTATTTTACCACCAGCTTTTTCGATAGCTTCTTTCGCACCTTTTGTGCAAGCTA<br>TACCCTGAA	50S ribosomal protein L15 [Francisella tularensis subsp. mediasiatica FSC147] >ref|WP_012429815.1| 50S ribosomal protein L15 [Francisella tularensis] >sp|B2SDW6.1|RL15_FRATM RecName: Full=50S ribosomal protein L15 >gb|ACD31330.1| 50S ribosomal protein L15 [Francisella tularensis subsp. mediasiatica FSC147]	40.0	40.0	33%	0.011	90%	Francisella cf. novicida 3523, complete genome	115	52%	1,00E-22	89%
>contig66300 length=290 numreads=13<br>ATAAGTTCAGCAGCTAGTCTATCACCCATAGTGTTTTCTTTACGCTTACGCGCAGCATCT<br>ATGATCCATCTCATACCCAAAGTTTGACGACGCTCAGGTCTAACCTCTACAGGTACTTGA<br>TAAGTAGCACCACCAACACGACGAGATTTAACCTCAACATGCGGACTTATGCTTTCTAAA<br>GCAGTTTCAAAAACTTCAACTTCATTTAATGAAGCATCTTTAGCTTTAATTCTATCAAAC<br>GCACCATAAACAATTTTTTCAGCTACTGATTTTTTACCATCTAGCATAAT	30S ribosomal protein S7 [Francisella sp. TX077308] >ref|WP_013922016.1| 30S ribosomal protein S7 [Francisella sp. TX077308] >gb|AEI35157.1| SSU ribosomal protein S7p (S5e) [Francisella sp. TX077308]	183	183	84%	1,00E-56	92%	Francisella cf. novicida 3523, complete genome	316	82%	7,00E-83	87%
>contig95182 length=221 numreads=10<br>TAAATACAGCTACTAAAGAAGCCTTACTAAGTCAGTTAGCAGATGACAGCCCTAACGGTT<br>TAATACTAGAAATtgATGAGTTGATGGACTTTATACAGTCAGTTATTAGAGCTGAAAAAA<br>TAGAAGACCATAAACTATATGTAGAGGCTTACAATAATGGAAGTTTCAAAAGCAAAACAA<br>TATCTAGGGGTacTCAATATATAGATAATGTAACAATATCT	hypothetical protein Fphi_0982 [Francisella philomiragia subsp. philomiragia ATCC 25017] >ref|WP_012280381.1| hypothetical protein [Francisella philomiragia] >gb|ABZ87206.1| conserved hypothetical protein [Francisella philomiragia subsp. philomiragia ATCC 25017]	147	147	79%	1,00E-39	96%	Francisella cf. novicida 3523, complete genome	311	79%	2,00E-81	92%

>contig13758 length=1492 numreads=222<br>TCTAAATAAAGAAAAATGATAACTGTAACAGACTCAGCTAAAAAACAaGCCATA<br>CGTCTC<br>ATGGAAGACGAGGGTCAGGACGGATACTTCATTCGCGTTGGCGTTGAGGGGGG<br>AGGATGT<br>TCTGGATTGATGTACAAATtGGACTTCGATAACGAGCATAAAGAAACAGACAAA<br>TCGTTT<br>GAAGACAACGGAATTGAAGTTGTTGTAGACAAAAAAAGCTTTTTATACCTCGTT<br>GGAACG<br>ACTCTAGATTTTTCTGGAGGACTGAATGGCAAAGGCTTCATATTTGTAAACCCGA<br>ATGCG<br>GATAGAACCTGCGGATGTGGTGAATCATTTTCAATATAATCGGAATTATGTCAAA<br>TTATA<br>CCGAAAACGAATTAGCAAAAGACCTTGAAGACCAAGAATATGAATTTGGTTTTA<br>CAACAG<br>ATATTGAATCTGACAAGATTCCTAAAGGACTTAGTGAAGAAGTAATTCGATTTAT<br>TTCTG<br>CTAAAAAAGAAGAGCCGCAATGGCTGCTTGATTTTAGGCTGGACTCTTATCGTA<br>AGTGGC<br>AAGACATGATCGAACCAGAATGGGCGCACGTTCATTATACGAAACCAGACTTTC<br>AAGATA<br>TAAGCTATTATTCTGCTCCAAAGCAGACTAAAAAATACGAGAGTTGGGATGATG<br>TTGATC<br>CTGAGATGAAGGAGACCATGAAAAAGTTGGGAATCTCGATGGAAGAGCAAAAA<br>CGCCTTA<br>CTGGAACGAACGTCGCAGTTGACTTCGTTATGGATTCCGTTTCTGTTGCAACTTC	Iron-regulated ABC transporter membrane component SufB [Fluviicola taffensis DSM 16823] >ref|WP_013686844.1| cysteine desulfurase activator complex subunit SufB [Fluviicola taffensis] >gb|AEA44074.1| Iron-regulated ABC transporter membrane component SufB [Fluviicola taffensis DSM 16823]	686	686	68%	0.0	86%	Francisella cf. novicida 3523, complete genome	283	34%	4,00E-72	76%
>contig57016 length=328 numreads=14<br>TTTAACTTCGTCAGCTAAATCTTCCATACcAACAacATCTGCTCCAGCTTCTTTAGC<br>TGC<br>TTCTGCTGCAGGACCTTTAGCAAATACTGCAACTCTTACAGATTTACCAGTACCAT<br>TaGg<br>AAGTACAGACGCACCTCTAACTACTTGATCAGATTTACGTGGATCTACACCCAAA<br>GCAAC<br>TGAAACATCTACCGCCtCAACAAACTTAACAGAAGTAAGCTCTTTTAAGATACCA<br>AATGC<br>TTCATTAGCAGAATACTTTTTTTCAGCTTCAACTTTAGCAGCGATATTTTTCATTCT<br>TTT<br>AGATAATTTAGACATTATTCAACTCCCT	50S ribosomal protein L1 [Francisella philomiragia subsp. philomiragia ATCC 25017] >ref|WP_012280432.1| 50S ribosomal protein L1 [Francisella philomiragia] >sp|B0TX07.1|RL1_FRAP2 RecName: Full=50S ribosomal protein L1 >gb|ABZ87265.1| 50S ribosomal protein L1 [Francisella philomiragia subsp. philomiragia ATCC 25017]	138	138	82%	1,00E-37	84%	Francisella cf. novicida 3523, complete genome	270	85%	6,00E-69	82%
>contig105103 length=206 numreads=8<br>ATTTAGAGGCAAGAGGTTTAGCTAAAACTTCTGATCGTAGAGTGTGGGCTTTCT<br>GTGGTG<br>ATGGTGAAATGGATGAACCAGAGTCAATTGGTTCTATCACAAGAGCTGGTCGAG<br>AAGGTT<br>TAGATAACCTTATATTTGTAGTGAACTGTAATTTACAAAGACTAGATGGTCTTGT<br>AAACG<br>GTAATGGTAATATTGTTGAAGAATTA	pyruvate dehydrogenase [Francisella philomiragia] >gb|EET20950.1| pyruvate dehydrogenase subunit E1 [Francisella philomiragia subsp. philomiragia ATCC 25015]	147	147	72%	7,00E-39	100%	Francisella cf. novicida 3523, complete genome	248	71%	2,00E-62	89%
>contig93901 length=223 numreads=7<br>ATCTCTTGCAAAGATTTTAACTTCACCAGTGTCGCTTGTATCTATTTGCGCACCAG<br>TTTT<br>TTCTGCAATACCTtttACAGTAGAACCACCACGACCAACAACATCTTTAATCTTAGC<br>TGG<br>TTTGATGTtcATAACATGAATTTGAGGAGCAACATTAGAAACAGTTTCTTTATGTT<br>CTTT<br>AATTACTTCATTCATAATACCAAGTATATGTAATCTACCACTA	polynucleotide phosphorylase [Francisella tularensis subsp. tularensis SCHU S4]	137	137	75%	2,00E-35	88%	Francisella cf. novicida 3523, complete genome	195	68%	3,00E-46	84%
>contig163622 length=146 numreads=6<br>GTTTCTTGTATGTCTTGGGCATTTTTAAAAGTTAAAATACCTGAAaaaGATATATA<br>CATA<br>CCCATATCTATAGCTTGTTTTGCCATaTCGTAATCTTCAGTAAAGCAATGCAGTAT<br>TCCG<br>CCACATTTTTCTACATTTTCAGATTT	deoxyribonuclease YcfH [Francisella sp. TX077308] >ref|WP_013922675.1| TatD family hydrolase [Francisella sp. TX077308] >gb|AEI35837.1| Putative deoxyribonuclease YcfH [Francisella sp. TX077308]	101	101	98%	9,00E-25	96%	Francisella cf. novicida 3523, complete genome	176	100%	4,00E-41	88%
>contig93979 length=223 numreads=11<br>TAAGATTCAAAAATCCTATCGACGGTATGGTAAGCTGGGATGATGCCGTAAAGG<br>GTAGAA<br>TTACAATAGCAAACCAAGAGCTTGATGATATGATTATTCAAAGAGCAGACGGTT<br>CGCCAA<br>CATATAACTTTTGTGTAGTTGTTGATGATATGGATATGAAAATATCTCATGTTATC<br>CGTG<br>GTGATGATCATGTAAATAATACTCCTAAGCAAATAAATATTTA	glutamyl-tRNA synthetase [Francisella sp. TX077308] >ref|WP_013922001.1| glutaminyl-tRNA synthetase [Francisella sp. TX077308] >gb|AEI35141.1| Glutamyl-tRNA synthetase [Francisella sp. TX077308]	145	145	87%	1,00E-39	92%	Francisella cf. novicida 3523, complete genome	171	84%	3,00E-39	83%

>contig171911 length=139 numreads=6<br>TGGTGCTAAAGGCGTAATAGTGAATATTACAGCTGGTATGGACATGACTATTGG<br>TGAATT<br>TGAAGAAGTTGGTGAGGTTATTAGATCATTTATTTCTGATGATGCTATTGTTATT<br>GCCGG<br>TACTGTGATTGATATGGAT	cell division protein FtsZ [Francisella novicida] >gb|EDX18862.1| tubulin/FtsZ family protein [Francisella tularensis subsp. novicida FTE]	84.7	84.7	64%	1,00E-18	93%	Francisella cf. novicida 3523, complete genome	158	62%	3,00E-35	88%
>contig182107 length=129 numreads=6<br>AAAAAGATGTGATGGAGTTTTtGCTTATAAACCATCCGCTTGATTGTCCAATATG<br>TGATC<br>AAGGTGGTGAGTGTGAGCTGCAAGATATAGCGATGGGTTAcGGTAAAACGACA<br>TCTGACT<br>ATAAAGAGT	NADH dehydrogenase subunit G [Francisella noatunensis subsp. orientalis str. Toba 04] >ref|YP_008893902.1| NADH dehydrogenase subunit G [Francisella noatunensis subsp. orientalis LADL--07-285A] >ref|WP_014714597.1| NADH dehydrogenase subunit G [Francisella noatunensis] >gb|AFJ43052.1| NADH dehydrogenase subunit G [Francisella noatunensis subsp. orientalis str. Toba 04] >gb|AHB98989.1| NADH dehydrogenase subunit G [Francisella noatunensis subsp. orientalis LADL--07-285A]	93.6	93.6	86%	1,00E-20	91%	Francisella cf. novicida 3523, complete genome	154	81%	2,00E-34	89%
>contig162313 length=147 numreads=9<br>AGAaCTATGCTTCCACTTTCTATGTCAGCAGATCATAGAGTAATTGATGGAGCGT<br>TAGCT<br>GCTAAGTTCTTGACTAGATATAATCAGATTCTTTCTGATTTACGCGAAATTATAAT<br>GTAA<br>ATTCAATATTAGCGGAGTTTAAAAAAA	dihydrolipoamide acetyltransferase component of pyruvate dehydrogenase complex [Francisella sp. TX077308] >ref|WP_013922551.1| dihydrolipoamide acetyltransferase [Francisella sp. TX077308] >gb|AEI35711.1| Dihydrolipoamide acetyltransferase component of pyruvate dehydrogenase complex [Francisella sp. TX077308]	79.0	79.0	59%	5,00E-16	97%	Francisella cf. novicida 3523, complete genome	154	61%	3,00E-34	89%
>contig126626 length=180 numreads=9<br>AAGGCGATGTtttATTAGAAGCAGGTACTTTACTTGATGAAAATCTTGTTGAGTTA<br>CTTG<br>ATGATAATGCTATTGATATGATCAAAcTtAGATCGCCAATTAGCTGTAATACTCGT<br>CGTG<br>GACTATGTGCTACTTGTTATGGTCGTGACTTGGCTCGTGAGAGAAAAGTAAACG<br>TTGGTG	DNA-directed RNA polymerase subunit beta' [Francisella sp. TX077308] >ref|WP_013922414.1| DNA-directed RNA polymerase subunit beta' [Francisella sp. TX077308] >gb|AEI35571.1| DNA-directed RNA polymerase beta' subunit [Francisella sp. TX077308]	84.0	84.0	85%	1,00E-16	85%	Francisella cf. novicida 3523, complete genome	147	75%	6,00E-32	83%
>contig84111 length=241 numreads=12<br>tATCAGAAACTTTTAGCACCAACAGATCTTGAAAATGACTCTtGTTATGTTGTTTC<br>AAAA<br>GCTGTAGATATTGCAAAGCTTTATGATGCAACACTTGATACAGCATTTGTAaTTC<br>CTAAT<br>GACAGTATTTCTCTTATGACATACGAGACTGATAAGGTAGAAGAATCACTTAAA<br>AAGTTT<br>GCAGATGCGAATGGtaTTAATGGTGATAAGTCCGTACTTATTGGTAGTaTAGCAA<br>cAaGT<br>G	universal stress protein [Francisella philomiragia subsp. philomiragia ATCC 25017] >ref|WP_012280178.1| universal stress protein [Francisella philomiragia] >gb|ABZ86969.1| universal stress protein [Francisella philomiragia subsp. philomiragia ATCC 25017]	127	127	78%	1,00E-33	74%	Francisella cf. novicida 3523, complete genome	147	67%	8,00E-32	79%
>contig177855 length=132 numreads=6<br>AttttttAATGATATTTAATACATTttCcGCCATTAATGGTTCAAAATAGAGCTTATCA<br>GA<br>AGTATCATAATCAGTTGAAACTGTCTCAGGATTACAGTTAATCATAATAGTTTCA<br>ATACT<br>CTCTTCAGCTAA	hypothetical protein [Francisella cf. novicida Fx1] >ref|WP_014549144.1| carbamoyl phosphate synthase large subunit [Francisella novicida] >gb|AEB26974.1| Carbamoyl-phosphate synthase large chain [Francisella cf. novicida Fx1]	89.0	128	62%	1,00E-18	95%	Francisella cf. novicida 3523, complete genome	139	59%	9,00E-30	87%
>contig173680 length=137 numreads=6<br>TATTTTGCACAGTGCCAACCATTTGTCTTGAGATGATTTGAGCATTACTTTTTAGT<br>AGTG<br>CTTGTAGTCTAGCTAAACGCTCTTTTTTAACTTCCATAGGAGTATCATCTGGTAAA<br>TCTG<br>CAGCTGGAGTACCAGGG	hypothetical protein [Francisella cf. novicida Fx1] >ref|WP_014549887.1| (dimethylallyl)adenosine tRNA methylthiotransferase [Francisella novicida] >gb|AEE87490.1| tRNA-i(6)A37 methylthiotransferase [Francisella cf. novicida Fx1]	84.0	84.0	90%	1,00E-17	85%	Francisella cf. novicida 3523, complete genome	134	79%	3,00E-28	86%
>contig152911 length=155 numreads=7<br>TTAATTCATATGCTATCAGATTCAGCAGGTGATTTTAGTGAGCTAAACATAGACT<br>aTCCA<br>TTTCCATTTTTGATAGCAGGGATTACAGTTTTGTTATTTTTATTAATGGAACATAT<br>TGGT<br>GGTACGCTATCTAAAAATAATAAAGGTAATCTATC	ZIP family zinc transporter [Francisella sp. TX077308] >ref|WP_013923135.1| zinc transporter [Francisella sp. TX077308] >gb|AEI36302.1| Zinc transporter, ZIP family [Francisella sp. TX077308]	99.0	99.0	86%	9,00E-24	92%	Francisella cf. novicida 3523, complete genome	122	88%	7,00E-25	81%
>contig168055 length=142 numreads=6<br>TAATAAGAATACATGTAAAaGAAGTTTAGTAAGGGTATTTACTTATTATGGTAAC<br>AACGG<br>TTTTAATTGTAATAATCTGTGTAGCATTATTTTTCGAGTTCACAAATGGTTTTCAT<br>GATG<br>CAGCAAATGTTGTTGCAACACC	inorganic phosphate transporter (PiT) family protein, partial [Francisella tularensis]	38.5	38.5	26%	0.094	100%	Francisella cf. novicida 3523, complete genome	97.1	31%	5,00E-17	95%

>contig129188 length=177 numreads=8<br>TACTAATATAGTAGCTTATTCaGGAACTCATGATAATAACACGCTGAAAGGCTGG<br>TATGA<br>TGATTTAAGTAAGTGgCAACGGAAACTTATCAAGAgaTATTTCAAAGCTAATGAT<br>AAAAA<br>TATCATCTCAAAAATATTAAAATACATGTGTTCATGTAATGCTGATATTGTAATAG<br>T	4-alpha-glucanotransferase [Francisella novicida] >gb|EDN37315.1| hypothetical protein FTDG_00097 [Francisella novicida GA99-3548]	95.1	95.1	88%	3,00E-21	73%	Francisella cf. novicida 3523, complete genome	80.5	58%	5,00E-12	79%
>contig92365 length=226 numreads=8<br>CAGATTCAATTGAAACAGTGATGTCTGCCCACTGGTATGATGGTTtAGTTTCAAT<br>ACCTG<br>GTTGTGATAAAAATATGCCTGGCTGTATGATGGCTTTAGGACGATTAAATAGAC<br>CTGGAT<br>TTGTTATATATGGTGGTACAATTCAAGCTGGCATAATGCGTGGTAAACCAATAG<br>ATATCG<br>TTACTGCTTTCCAAAGTTATGGAGCATGCTTATCTGGTAGTATCAC	dihydroxy-acid dehydratase [Francisella noatunensis subsp. orientalis str. Toba 04] >ref|WP_014715277.1| dihydroxy-acid dehydratase [Francisella noatunensis] >gb|AFJ43747.1| dihydroxy-acid dehydratase [Francisella noatunensis subsp. orientalis str. Toba 04]	158	158	64%	5,00E-44	97%	Francisella cf. novicida Fx1, complete genome	235	65%	2,00E-58	85%
>contig114906 length=193 numreads=10<br>TCACATACTAGGTAGACAGCCATATAATACTAAAAATACTGCTAGAGATAGTTTT<br>ATAAC<br>TGCTTTAGTTACTGGTGGTGAAGGTTATCATAATTATCACCATGCATTTGCTGGT<br>GACTA<br>TAGAAATGGTATTAGATGGTTTGATTTTGATCCTTCTAAATGGCTTATCGCAGGT<br>TTAGC<br>TAAAATAGGCTGG	fatty acid desaturase [Francisella sp. TX077308] >ref|WP_013922417.1| fatty acid desaturase [Francisella sp. TX077308] >gb|AEI35574.1| Fatty acid desaturase; Delta-9 fatty acid desaturase [Francisella sp. TX077308]	126	126	79%	7,00E-33	88%	Francisella cf. novicida Fx1, complete genome	191	79%	3,00E-45	85%
>contig56997 length=328 numreads=15<br>TGTAAACGCATCTATTGATGGTGACGATATTGTTTATCATAACTATTTTGACATAA<br>GTAT<br>AGCAGTTGGTACAGACCGAGGCTTAGTTGTTCCTGTGTTGAAAGATGTTGATGA<br>TATGAG<br>TTTGGCAGATTTAGAAAATGCTGTAGTTGATAAGGCTATTAAAGCCAGGGATGG<br>TAAACT<br>AACTCTTGAAGATATGCAAGGTGGTACATTTACTATTACTAATGGTGGTACTTAT<br>GGTTC<br>TATGCTTTCTACTCCAATCATCAATTCACCACAAaGTGCTATATTAGGCATGCATA<br>ATAT<br>CGTTGAAAGACCAATGGTTGTAAATGGT	dihydrolipoamide succinyltransferase component (E2) of 2-oxoglutarate dehydrogenase complex [Francisella sp. TX077308] >ref|WP_013922347.1| dihydrolipoamide succinyltransferase [Francisella sp. TX077308] >gb|AEI35504.1| Dihydrolipoamide succinyltransferase component (E2) of 2-oxoglutarate dehydrogenase complex [Francisella sp. TX077308]	201	201	75%	2,00E-59	88%	Francisella cf. novicida Fx1, complete genome	191	70%	5,00E-45	78%
>contig111296 length=197 numreads=8<br>AAGTGTTTTTGTTTGATCTTTTGAACTTAGTTAAGCTATAAATATCTACTAAGTTA<br>TTAT<br>TCTTAGATTTTTTAGTATTTACCTTAacTACAATTCTATTTGAATCAACTTCTGCAAT<br>AG<br>TACCAGGGTTTCTAGcAACGATACAGTTACCTGAATCTCTAGCAACAATCTTCTCC<br>ATAC<br>CAGTACCAACCATTGGC	DNA-directed RNA polymerase subunit beta [Francisella sp. TX077308] >ref|WP_013922413.1| DNA-directed RNA polymerase subunit beta [Francisella sp. TX077308] >gb|AEI35570.1| DNA-directed RNA polymerase beta subunit [Francisella sp. TX077308]	94.7	94.7	70%	3,00E-20	85%	Francisella cf. novicida Fx1, complete genome	147	67%	7,00E-32	81%
>contig164872 length=145 numreads=6<br>CAACGATACCAATACCATTAAACTCTTTCAATGACTCTTCGTTAGCTAATCTTTCG<br>ACTA<br>ACTCTAGAGATATTTGTAACCTTTCAGTTTCTTCAGCATCAATATTCATACCAACA<br>TTAT<br>ATTGTTTTGCTAACTGAGTCAATTT	proline dehydrogenase [Francisella sp. TX077308] >ref|WP_013923002.1| pyrroline-5-carboxylate dehydrogenase [Francisella sp. TX077308] >gb|AEI36168.1| Proline dehydrogenase (Proline oxidase) / Delta-1-pyrroline-5-carboxylate dehydrogenase [Francisella sp. TX077308]	87.0	87.0	62%	1,00E-17	90%	Francisella cf. novicida Fx1, complete genome	124	58%	3,00E-25	83%
>contig168285 length=140 numreads=7<br>TCtCTTTACAAGTACCAGGTTCACCCTCATCTGAGTTACAAACCACATACTTTTGA<br>CCAG<br>GCATACTACGGGGCATAAAGCTCCATTTCAAACCTGTTGGGAAACCAGCACCAC<br>CACGAC<br>CTCTAAGACCAGACTTTTTT	NADH dehydrogenase [Fangia hongkongensis]	95.9	95.9	84%	4,00E-22	93%	Francisella cf. novicida Fx1, complete genome	126	82%	5,00E-26	84%
>contig100890 length=212 numreads=12<br>GCTATAGTAACTTTGATAGCCTCGACCATTGTCTAAAGAATAAACCTTATAaCcTT<br>GCTC<br>TTCTGTGGCTAATCTATCCGTAACAGGATTAACCATATAAAAGCTAGCAGTAGAT<br>GTtCC<br>ACCTAAAATTTGCTTAGCTTGTGTAGCATCTTGCATACCTGGTATCTCAATAACCA<br>CACG<br>GTTTGCACCTGCTTGAGCAACTGATGCTTCAG	Protein-export membrane protein SecD [Francisella sp. TX077308] >ref|WP_013923354.1| preprotein translocase subunit SecD [Francisella sp. TX077308] >gb|AEI36523.1| Protein-export membrane protein SecD [Francisella sp. TX077308]	139	139	86%	1,00E-36	94%	Francisella cf. novicida Fx1, complete genome	228	87%	2,00E-56	86%
>contig135083 length=171 numreads=6<br>GAAGGTCGACCGTTATTTGATGATGAAAACCAACAGATTACAATGCTTTCAAGC<br>AATCAA<br>GTAGCTCTTAATTTTGTGGATAACAATGGTAGTAAAACTGAAAATTATCCACAGA<br>ACCCT<br>AATGGTGCAATAGATGGTCTAACAGCTGTTACAGCCCTAAATGGTCGTGTA	Phosphoribosylformylglycinamidine synthase, synthetase subunit / Phosphoribosylformylglycinamidine synthase, glutamine amidotransferase subunit [Francisella cf. novicida 3523]	87.4	87.4	77%	6,00E-18	68%	Francisella cf. novicida Fx1, complete genome	82.4	29%	2,00E-12	89%

>contig58976 length=318 numreads=11<br>GGACTAATCGTAGCACCCCCAAAGACTGGTAAAACAATCATGATGCAAAATATC<br>GCAACT<br>TCAATTGCAAAAAACCATCCTGAATgtAACCTAATCATGCTTtTAATTGATGAAAG<br>ACCA<br>GAGGAAGTAACAGAAaTGCAACGCTCTGTGCGTGGTGAAGTTGTAGCAAGTAC<br>ATTTGAT<br>GAACCAGCTGCTCGCCATGTACAGCTAGCAGAGATTGTAATCGAAAAAGCTAAA<br>AGATTA<br>GTTGAGCATAAACAAGATGTAGTAATCTTACTTGACTCAATCACAAGACTTGCTC<br>GTGCA<br>TACAACACAGTATCTCCT	transcription termination factor Rho [Francisella tularensis subsp. holarctica F92] >ref|WP_015083803.1| transcription termination factor Rho [Francisella tularensis] >gb|AFX70309.1| transcription termination factor Rho [Francisella tularensis subsp. holarctica F92]	218	218	87%	1,00E-68	100%	Francisella noatunensis subsp. orientalis LADL--07-285A, complete genome	359	85%	1,00E-95	87%	
>contig90038 length=230 numreads=11<br>AGCcTACATTTACTTGTCTATCAAAACGACCAGGTCTTAAAAGTGCATTATCTAGC<br>ACAT<br>CTGGTCTATTAGTAGCAGCAATTACAATTACACCTTCATTATCTGCAAAACCATCc<br>aTTT<br>CTACAAGTAGTTGGTTAAGAGTTTGCTCACGCTCATCATTACCACCACCCATACCT<br>GAGC<br>CACGATGACGACCAACTGCATCTATCTCATCAATAAATACCAAGCATGGA	ATP-dependent metalloprotease [Francisella philomiragia] >gb|EET21773.1| ATP-dependent metalloprotease [Francisella philomiragia subsp. philomiragia ATCC 25015]	125	125	75%	3,00E-31	97%	Francisella noatunensis subsp. orientalis LADL--07-285A, complete genome	254	72%	5,00E-64	88%	
>contig151752 length=156 numreads=7<br>CAGTGTCTAAATCAGATGCTGTTATTTTTAACTTATTATCTTGTATGTCAAATAAA<br>ATAC<br>ATGACAGTAATGGCATAGTACTTTTGCTATTtgCTATAGATAACATTGATTGCAAA<br>GGCT<br>TAAGCAAGTCATCTCTATTTAGTACAAAATTCATTT	DNA polymerase III subunit beta [Francisella tularensis] >gb|EET20098.1| DNA polymerase III [Francisella tularensis subsp. tularensis MA00-2987]	94.4	94.4	82%	9,00E-22	92%	Francisella noatunensis subsp. orientalis LADL--07-285A, complete genome	132	78%	1,00E-27	83%	
>contig52685 length=350 numreads=17<br>AGAGTCTGGTAAAGTTAGATTTGTGCCGGATAATTGGAAAAACACTTACTACTC<br>ATGGAT<br>GCGTGATTTACAAGATTGGTGCGTGTCAAGACAGCTTTGGTGGGGTCATAGAAT<br>ACCTGC<br>TTGGTTTGATAATGATGGTAATGTATTTGTTGGCGAgtCAGAAGAACAAATCCGT<br>ACAAA<br>GCATAAtCTAGCTGAGAGCATAGAGCTTAATCAAGACAATGATGTATTtGATACA<br>TGGTT<br>CTCATCTGCATTGTGGCCATTTAGTActCTAGGTTGGCCTGAGCAAACTCCTGAGC<br>TAGC<br>TAAATACTATCCTACAAGCGTGCTTGTAACAGGTTTTGATATTATCTTCT	valyl-tRNA synthetase [Francisella tularensis subsp. tularensis WY96-3418]	215	215	89%	1,00E-62	81%	Francisella noatunensis subsp. orientalis LADL--07-285A, complete genome	241	84%	5,00E-60	80%	
>contig132923 length=173<br>numreads=6GTGCAAATGAAGCTGTTCTTAAAATGCTTGCTGAAATAGAGAGTG<br>TTGAAAATATTGATA<br>AATTTATAGCAaAAGCAAAAGATAAGAATGATCCATTTAGACTAATGGGCTTTG<br>GTCATA<br>GAGTTTACAAAAACACTGACCCAAGAGCTACAGCTATGAAAGAAAATTGCGAA	GltA [Francisella sp. TX119]	112	112	87%	3,00E-29	88%	Francisella noatunensis subsp. orientalis LADL--07-285A, complete genome	176	87%	6,00E-41	85%	
>contig78870 length=254 numreads=10<br>ATCGTTGCTAAAATAATATCAGGATTTGCAGTATGAGTAAGATAATAGATGTAG<br>AAACAA<br>GCATAATTAAAACACCTTTAAAAAGAACCTTTACAACAGCGGTAAGAAGTACTA<br>ACCATA<br>TAGACTCTCTAGTCTTAAAGATAACTACTGATGATGgTTTAGTTGGTTTTGGAGTT<br>GCTC<br>caGCTACTACGGCGATTACTGGTGATACCCTtgCAGGGATGAAGTATATTGCTGA<br>AGAAC<br>TTTTTACACCTGTT	Muconate cycloisomerase [Francisella cf. novicida 3523] >ref|WP_014547998.1| mandelate racemase [Francisella novicida] >gb|AEB28546.1| Muconate cycloisomerase [Francisella cf. novicida 3523]	119	119	79%	3,00E-30	76%	Francisella noatunensis subsp. orientalis LADL--07-285A, complete genome	134	82%	6,00E-28	77%	
>contig66992 length=288 numreads=12<br>CTCTTAGAGCTAAAAGTTTAGGATGTCTGTATAGAGCATTTGAATCAAGATTGAT<br>TTTAC<br>CATCAACACATACGATCTCACCATTTTCTCTTACAGCAAGAGGGTTAATCTCAAaT<br>AGTG<br>CAAAGTCACATTCAACAAATGCTTTGTAaGCACCTATCATAGTCTTTGTGAAGTC<br>GTTGA<br>TTTgTTtGCCTTCTAAGCCTAGTTGAAAAGCAACATCACGAGCTTGGAATGGTTG<br>AAGGC<br>CTACTAATGGATCTACTTCTACTTTAAGAATTTTTTCTGGAGTGTTAT	succinyl-CoA synthetase subunit beta [Francisella philomiragia subsp. philomiragia ATCC 25017]	193	193	84%	1,00E-57	96%	Francisella noatunensis subsp. orientalis LADL--07-285A, complete genome	307	84%	4,00E-80	86%	

>contig77912 length=256 numreads=10<br>ATGATGCATGTCCTACAAGGTGGTGCAGCTGCAAAACCATTTAGAACTCATCATA<br>ATGCC<br>TTAGATATGCCGTTGTACTTGCGTATAGCTCCAGAGCTTTATCTAAAAAGATTAG<br>TTGTT<br>GGTGGTTTTGAGCGTGTTTACGAAATAAACCGTAATTTCCGTAATGAAGGTGTAT<br>CTTCA<br>AGACATAATCCTGAATTTACTATGCTAGAGTTTTACATGGCGTATGCTGATTATA<br>ATGAT<br>CTTATGGATCTAACAG	lysyl-tRNA synthetase [Francisella cf. novicida 3523] >ref|WP_014547489.1| lysyl-tRNA synthetase [Francisella novicida] >gb|AEB28010.1| Lysyl-tRNA synthetase (class II) [Francisella cf. novicida 3523]	182	182	90%	2,00E-52	99%	Francisella noatunensis subsp. orientalis LADL--07-285A, complete genome	302	90%	2,00E-78	88%
>contig153082 length=155 numreads=8<br>AAAAAGAGCAAGgTATAGATCTTCATAATGATAAGCTAGCTCTACAAAGAGTAA<br>GAGAAG<br>CAGCTGAAAAAGCTAAAGTAGAATTGTCTTCTGCTCAGCAAACAGATGTAAACT<br>TACCTT<br>ATGTAACAGCTGATGCTACAGGACCTAAACATTTA	chaperone protein DnaK [Francisella guangzhouensis]	106	106	79%	5,00E-25	96%	Francisella noatunensis subsp. orientalis LADL--07-285A, complete genome	209	79%	6,00E-51	91%
>contig155456 length=153 numreads=8<br>GCTAGATCTCATGCTAACTATGCTTTTTACTTAGGTGCTACAAATGACAATGTCG<br>AAGAG<br>CTAAAACGCCTGAAACCAAATGATGCTTGTGCTATTAAGATATTCATGGGTGCTT<br>CAACT<br>GGTAATATGCTAGTCAATAACAAAGAAACTCTT	dihydroorotase [Francisella sp. TX077308] >ref|WP_013921817.1| dihydroorotase [Francisella sp. TX077308] >gb|AEI34954.1| Dihydroorotase [Francisella sp. TX077308]	106	106	81%	9,00E-26	98%	Francisella noatunensis subsp. orientalis LADL--07-285A, complete genome	182	71%	1,00E-42	91%

**Contigs**	**Blastx**	**Max score**	**Score**	**Query Cover**	**e-value**	**Identity**	**Blastn**	**Score**	**Query Cover**	**e-value**	**Identity**
>contig98282 length=216 numreads=9<br>CCCATAGCGATAGTTCTAACTACACCATCACCGATTTGTTGTTGCACTTCTAGTAC<br>AAGA<br>GATGCCTCTTCTACTTTTAATGCTTCATATACTTTTGGTGTATTATCGCGTGAAAA<br>CTCC<br>ACATCGATAACAGGACCAATTACTTGAATAATTTTACCAGTACTCATTTATCTCTC<br>CTAA<br>ACTGCTGCTGCACCTGAACAAATTTCAGCAAGCTCT	F0F1 ATP synthase subunit beta [Francisella philomiragia subsp. philomiragia ATCC 25017]	102	102	63%	5,00E-24	91%	Francisella noatunensis subsp. orientalis LADL--07-285A, complete genome	261	82%	2,00E-66	88%
>contig100997 length=212 numreads=8<br>ACTGATAGTTTAGCTGGGCTAGTTCTACTTGTAGCTTACCTTCATGAGTTTTAGCC<br>CTTA<br>ATGAAAATATTTCAAGAATAAGCCTAGTTCTATCcaTGATTTTACACTCAAGAAAT<br>TTTT<br>CAATATTTCTCTCTTGAGATGGGCTTAATGGgTGGTTAAAAACAACTACATCAGC<br>ATCCA<br>ACTCATCGCGTTTGTTTTTTATCATTTCCATT	GTP-binding protein HflX [Francisella sp. TX077308] >ref|WP_013923337.1| protease [Francisella sp. TX077308] >gb|AEI36505.1| GTP-binding protein HflX [Francisella sp. TX077308]	142	142	82%	2,00E-38	97%	Francisella noatunensis subsp. orientalis LADL--07-285A, complete genome	231	83%	2,00E-57	86%
>contig155433 length=153 numreads=6<br>TttGAGCTTGAGATACTAGCCATTTGTTTACAACTAAATCTTCATGTTTCCAACGG<br>TTAT<br>AAAACTCATTGATAACATTATCACGAATCTGCTTATCATTTGACTTTAAAAGTGCT<br>GAAA<br>ATGCAGTTTGCTGGTCTGTCATATTATCAGCAT	aminopeptidase N [Francisella philomiragia subsp. philomiragia ATCC 25017] >ref|WP_012280259.1| aminopeptidase [Francisella philomiragia] >gb|ABZ87056.1| aminopeptidase N [Francisella philomiragia subsp. philomiragia ATCC 25017]	102	102	80%	1,00E-23	94%	Francisella noatunensis subsp. orientalis LADL--07-285A, complete genome	204	81%	3,00E-49	91%
>contig65820 length=291 numreads=14<br>CGATTCTACCTTCCTGATAACTTCGGTATCATGGTTTATACTTTGCGTAGAATGAG<br>AAAT<br>ACTATCGATAATCTTGTAGTACAAAAAGATATTATCGAAGATAGAGTAAAGGGT<br>ACAAGT<br>GCATACCTATCAAGCTTCTACCTACACTTCTTGATtGCAAACACTgAGTTTATGCG<br>TGAA<br>GATTGCTACAAAATCgTACAGCAAGTAGCATTTGAGCTTAAAGCTGGCGAATCAT<br>TCTCA<br>CAAAAGCTACAAGCTATTATGAAGTctGAGCATGGTATCGACCTAAGCGTT	adenylosuccinate lyase [Francisella novicida] >gb|EDN36974.1| adenylosuccinate lyase [Francisella novicida GA99-3549]	174	174	92%	4,00E-50	81%	Francisella noatunensis subsp. orientalis LADL--07-285A, complete genome	198	72%	2,00E-47	82%
>contig119776 length=186 numreads=6<br>CAATCCCTGTGCCTTCAATATCATCATTAAAAGTACAAAGCCTTTCTTTATATTTCT<br>CAA<br>GAATACGAGTAGCATTATCACGACCAATATCTTCCCAGTGCAAAAACACATTTGG<br>GAATC<br>TATTTTTAGCTTGAACTACAAACTCTTCTATAAACTCATCATaaGTTTCACCTGAGA<br>CTC<br>TTGGTA	malate dehydrogenase [Francisella philomiragia subsp. philomiragia ATCC 25017] >ref|WP_012279885.1| malate dehydrogenase [Francisella philomiragia] >gb|ABZ86612.1| Malate dehydrogenase (oxaloacetate-decarboxylating) (NADP(+)) [Francisella philomiragia subsp. philomiragia ATCC 25017]	126	126	88%	2,00E-32	89%	Francisella noatunensis subsp. orientalis LADL--07-285A, complete genome	196	88%	5,00E-47	86%

>contig104939 length=206 numreads=10<br>AaGCGATTGAAGTATAAAATCATTCCAGGGTTAGTTGCTGGAATTGTTGAACGA<br>GGAATA<br>TTTATTTCATCAAAATCATTATAAGTTTGTGACCAAAAAACACTACcAATAGCATC<br>ATTA<br>AAaGAGTGTATATCATTGTTgAACTTTTCTTTTAAGTATTTTTTAAACTCAATTTCA<br>CAT<br>TGATCACAATAACACATATCACTTGT	beta-galactosidase [Francisella cf. novicida 3523] >ref|WP_014547803.1| beta-galactosidase [Francisella novicida] >gb|AEB28345.1| Beta-galactosidase [Francisella cf. novicida 3523]	133	133	90%	1,00E-34	86%	Francisella noatunensis subsp. orientalis LADL--07-285A, complete genome	183	88%	5,00E-43	83%
>contig109684 length=199 numreads=8<br>TGTTGCTTAATtAGGTTATTTTCTAAAAGATATGCTTCATAATCACTTGGTGTAAT<br>AGTT<br>ATcTCTATTTTCTCAACTTGTGATGCCATTAGTAAAGTTTTATTATCTTTCGCACCT<br>TTT<br>GAGAAATAGCTATTTACCCGGTTTTTGAGATTTTTTGCTTTACCAACATAGATTAT<br>CTCA<br>CCATTAGCATCAATCATTC	excinuclease ABC subunit C [Francisella sp. TX077308] >ref|WP_013923256.1| excinuclease ABC subunit C [Francisella sp. TX077308] >gb|AEI36424.1| Excinuclease ABC subunit C [Francisella sp. TX077308]	119	119	88%	2,00E-29	85%	Francisella noatunensis subsp. orientalis str. Toba 04, complete genome	169	82%	1,00E-38	83%
>contig182245 length=129 numreads=6<br>CTCTTTGAAAAGTCCAGCAACCATAGTTTCCATCGTAGACATAATGTATTCTTCAT<br>CAAT<br>AAATGAAGCCTCGATATCTATTTGTGTAAATTCTGGCTGTCTATCTGCTCTTAAAT<br>CTTC<br>ATCACGGAA	Aspartyl-tRNA(Asn) synthetase [Francisella sp. TX077308] >ref|WP_013921913.1| aspartyl-tRNA synthetase [Francisella sp. TX077308] >gb|AEI35052.1| Aspartyl-tRNA synthetase / Aspartyl-tRNA(Asn) synthetase unambiguous [Francisella sp. TX077308]	82.8	82.8	81%	5,00E-17	91%	Francisella noatunensis subsp. orientalis LADL--07-285A, complete genome	159	75%	5,00E-36	91%
>contig154187 length=154 numreads=8<br>CTTGTAAACTATAGCTGGAGCGTGGGCAGTCTTTTGTACTACTTGCTCAAGAgTtA<br>agAA<br>GCCATTGTGATGATGCTCTAGTACAGATATAAGAGCCTCTTCAACTAATGGTAAG<br>CCAGC<br>AGGTGCTTTAAAGTAAGTACCTTGCTTTTCTTCC	dihydroorotase [Francisella sp. TX077308] >ref|WP_013921817.1| dihydroorotase [Francisella sp. TX077308] >gb|AEI34954.1| Dihydroorotase [Francisella sp. TX077308]	103	103	89%	7,00E-25	98%	Francisella noatunensis subsp. orientalis LADL--07-285A, complete genome	156	87%	7,00E-35	85%
>contig148550 length=159 numreads=6<br>AAATCCAAATGCCAAAACTACTTGTGGTTGTGGCTCATCTTTTTCTGTATAATAAT<br>CTTA<br>CATATTTTTTTCAAAGAACATTTTAATTAGAGAGTACTATGAAAATTGCAAATTTT<br>GAAG<br>TAGGTAaTGGTAAACCATTTTTTTTAATGTCTGGACCAT	2-dehydro-3-deoxyphosphooctonate aldolase [Francisella tularensis subsp. tularensis SCHU S4	45.4	45.4	28%	6,00E-04	100%	Francisella noatunensis subsp. orientalis LADL--07-285A, complete genome	150	100%	3,00E-33	85%
>contig85088 length=240 numreads=8<br>AAATTTGATGAGTTtttCTTTGCAGAAAAGGGTATAGCTATTAAAAATCCAATCTC<br>TCAA<br>GATTTATCAGTGATGAGACAGtcACTTATACCAGGGCTTTTGAATGCTTTTAAAGC<br>AAAT<br>GTAACTCGCCAACAAACGAGGGTTAGATTGTTTGAGAAGGGTGCTTGTTTTAAA<br>CTTGAG<br>AATGGTACTAGAAAACAGTTTGATAGAATTGCAGGTCTAGCATTTGGTGAGCTA<br>AATAGC	phenylalanyl-tRNA synthetase subunit beta [Francisella cf. novicida 3523] >ref|WP_014548208.1| phenylalanyl-tRNA synthetase subunit beta [Francisella novicida] >gb|AEE26207.1| Phenylalanyl-tRNA synthetase beta chain [Francisella cf. novicida 3523]	124	124	72%	7,00E-31	82%	Francisella noatunensis subsp. orientalis LADL--07-285A, complete genome	150	64%	6,00E-33	82%
>contig90173 length=230 numreads=9<br>TTCACTAATTTTATTTTATTAATCATGTCACAGACTGTAGCTTCCTCTTCAACTTGC<br>TCA<br>TCTATAAACCATTGTAAAAAGCTTTTAGTTGCATGCTCTTTCTCATCTAAAGCAAT<br>ATCC<br>ATTAGGTGATAAAGTTTTgATGTTATATCTTCCTCAtgTCTTAATGTTTCTTCGAAA<br>ACT<br>TCAAGTAGCGAGTTAAAATGATTTTGAGGAGCAGCAGTTGCTTGAACTTC	ferritin [Francisella sp. TX077308] >ref|WP_013923318.1| ferritin [Francisella sp. TX077308] >gb|AEI36486.1| Ferritin [Francisella sp. TX077308]	129	129	95%	7,00E-36	78%	Francisella noatunensis subsp. orientalis LADL--07-285A, complete genome	147	89%	6,00E-32	79%
>contig185798 length=125 numreads=6<br>TAGCTTTCAAAATAGCCTCAATTGCAGCTTCATTTGATGGAAGGTCTGGGGCATA<br>ACCAC<br>CCTCATCACCAACACCAGCAACACTATAACCATCGTTAACTAACACTTTTTTAAGT<br>GTAT<br>GGAAT	phosphopyruvate hydratase [Francisella novicida U112] >ref|WP_003035924.1| enolase [Francisella novicida]	68.2	68.2	90%	4,00E-12	95%	Francisella noatunensis subsp. orientalis LADL--07-285A, complete genome	137	89%	2,00E-29	87%
>contig149725 length=158 numreads=7<br>TAGCTAATGATAAAGGTGTACTTGGGCGTAATGGTCATACTGAAGCAACAGTTG<br>ATTTGA<br>TGAAATTATCAGGTTTtAATAGTGCTGGTGTTTTATGTGAGCTGATGAATAAAAA<br>TGGTA<br>CTATGATGAATGGTGACGAACTTGAACGATTTGCTAAA	GTP cyclohydrolase II [Francisella philomiragia subsp. philomiragia ATCC 25017] >ref|WP_012280147.1| 3,4-dihydroxy-2-butanone 4-phosphate synthase [Francisella philomiragia] >gb|ABZ86936.1| GTP cyclohydrolase II [Francisella philomiragia subsp. philomiragia ATCC 25017]	99.4	99.4	83%	2,00E-23	89%	Francisella noatunensis subsp. orientalis LADL--07-285A, complete genome	137	68%	3,00E-29	86%

>contig72436 length=271 numreads=8<br>CACACTTACATATTTCAACTACAAAGTtttCAACTAACTGTTGCCCATGTTCTGTAT<br>GAG<br>TAGTTTCAGGATGGAACTGCACACCATAAAAAGGTTTAGTTTTATGAGATACAG<br>CTGCAA<br>TTGGAGCATTTATAGATGAAGCAATTATCTCAAAATGTTCACCGGTATCTGTAAC<br>TTTAT<br>CGCTATGGCTCATCCAAACAAGTTGTTCAGAACCTAGCTTTGCAAATAACTTATTT<br>TCAT<br>CTTGTGCTATATTTATTATAGCTTTACCAAA	GMP synthase [Francisella novicida] >gb|EDN36210.1| GMP synthetase [Francisella novicida GA99-3549]	125	125	92%	8,00E-32	76%	Francisella noatunensis subsp. orientalis LADL--07-285A, complete genome	134	61%	6,00E-28	80%
>contig117242 length=190 numreads=12<br>TTTCTTAAAATCATTAAGTTTTTTCTCATCATCACAGAAGACCCAACGAGCTTGAA<br>CCAC<br>ATTCACACCTTCGTACGAGCATTTTACATTGTACTCACTAGCTAAGCGTTGTGCTA<br>CTAC<br>ATCAAACTGAAGTACACCTACCGCACCAAGCACGAGATCATTTGAAATCATTGGT<br>TTAAA<br>CACTTGCGTA	peptide chain release factor 3 [Francisella philomiragia] >gb|EET20777.1| peptide chain release factor [Francisella philomiragia subsp. philomiragia ATCC 25015]	130	130	87%	6,00E-34	95%	Francisella noatunensis subsp. orientalis LADL--07-285A, complete genome	130	88%	6,00E-27	79%
>contig81714 length=247 numreads=11<br>TCCCATTCAAACACATTAGCTCTACCTCTATTCTCTTTAGGATCATTTTCATAACTC<br>TTA<br>AGAGCATTTGAAATACTTtCAAAACTAATACCTAATTTAAATGATAAAGCTATAG<br>CAACT<br>AATGCATTTTCAATATTATGCTTAGCAAAaCCTTTAACAGTTAGAGGTGCATCAA<br>CCACT<br>TTTAAAAGAGATTTTTTAACACCcTTTTCAATCCATATAAACTCTCCATTCTCAACA<br>ACA<br>CTCGCAT	Mur ligase [Francisella novicida] >gb|EDN37892.1| conserved hypothetical protein [Francisella novicida GA99-3548]	129	129	93%	2,00E-33	72%	Francisella noatunensis subsp. orientalis LADL--07-285A, complete genome	126	66%	9,00E-26	80%
>contig83316 length=244 numreads=11<br>TAGAGCTATCTCTATCAAAAAACTTAGCTGGAAGATAAATCATATCATCATAAGT<br>TATTG<br>CCTTTTGATTCTCACACTCTTTATTAAAAGCTTCGAAAACCTTATCCAGTAATTTTT<br>TAT<br>CTTTACTATTTATAGCAAGAATTTTTTTATTTGAAAGGCTTAGGTCAGATTTTAAA<br>AGAG<br>AGATATATTCTTTAAAAGTTTCAACTCTTTCAGTATCAATATCTTTGATTATCTTCA<br>GCT<br>CTTT	ATP-dependent DNA helicase UvrD/PcrA/Rep family, Francisella type [Francisella cf. novicida 3523] >ref|WP_014548398.1| DNA helicase [Francisella novicida] >gb|AEE26402.1| ATP-dependent DNA helicase UvrD/PcrA/Rep family, Francisella type [Francisella cf. novicida 3523]	100	100	84%	1,00E-22	77%	Francisella noatunensis subsp. orientalis LADL--07-285A, complete genome	108	76%	4,00E-20	76%
>contig84437 length=241<br>numreads=8CCATTAATGAACTGTACTTATAAAGGAACTCAACGGAATAATGATT<br>TCGCTATATTTAAT<br>GACACCTTTCGTGATGCTGTAAGAGGAAATAATGCTCCAGGAAATGGGTTTATT<br>AATGGT<br>TCAACTCATAATAGTGAATTAATGGGTAAAGTTGTTGAAGGTTTATATGGCTCAG<br>TTCAC<br>ACACTAACCGCTAACGCAAATGAATCAGTAAACTATGTTGATGCTCATGATAACT<br>ATACT<br>C	pullulanase [Francisella philomiragia] >gb|EET21947.1| pullulanase [Francisella philomiragia subsp. philomiragia ATCC 25015]	125	125	92%	3,00E-31	69%	Francisella noatunensis subsp. orientalis LADL--07-285A, complete genome	91.6	87%	3,00E-15	74%
>contig111572 length=197 numreads=12<br>TttCGGTACCAAATACCTAATAAAGCAAGAATAACAGGTAAGTTTTTTTCTAACG<br>GAGCA<br>TGAGTGAAATGTTCATCCATTTCAAACGCACCTTGTAAAAAATTGCGGTAATTAT<br>CAAAC<br>CCTATCGCTAATGCTAAAGGTAAACCTATCGCTGACCAGACAGAATAGCGACCA<br>CCAACC<br>CAATCCCaCATCGGAAA	glucose-6-phosphate isomerase [Glaciecola psychrophila 170] >ref|WP_007636341.1| glucose-6-phosphate isomerase [Glaciecola psychrophila] >dbj|GAC36712.1| glucose-6-phosphate isomerase [Glaciecola psychrophila 170] >gb|AGH42824.1| glucose-6-phosphate isomerase [Glaciecola psychrophila 170]	127	127	80%	7,00E-33	80%	Francisella noatunensis subsp. orientalis LADL--07-285A, complete genome	60.2	19%	8,00E-06	89%
>contig140773 length=166 numreads=8<br>CCACcACCAGCAACTATACCTTCTTCTACAGCAGCACGAGTTGCATGAAGTGCAT<br>CATCT<br>ACACGATCTTTTTtcTCTTTCATCTCAGCTTCTGTAATAGCACCAACCTTAATTACT<br>GCT<br>ACACCACCAGAAAGCTTTGCTAACCTTTCTTGAAGTTTTTCACGGT	GroEL, partial [Francisella sp. LA112445]	83.6	83.6	62%	8,00E-18	95%	Francisella philomiragia strain CCUG 19701 GroEL (groEL) gene, partial cds	220	73%	3,00E-54	93%
>contig78846 length=254 numreads=7<br>AAAAGATGATAGTGAAGAGCCTAAAGAaGAAGAGTTTAAAGAAAAAGAAGTTA<br>GAAAAAA<br>TACTAAAAAAGAGAAAAATTCTTCACAAGAATTATTTGGTTTTGATGAAGCTATG<br>CTTAT<br>CACTGCTAAAGAAGGTGAAGAATATTCATTTGGTTtACCTTTAAAAGATAAATAT<br>ATTTT<br>TGATAGTTTTGTTGTAGGAGATGCTAATAAAATAGCTAGAGCCGCAGCAATGCA<br>AGTATC<br>GATAAATCCAGGAA	replication initiator [Francisella tularensis subsp. holarctica] >emb|CAJ98758.1| replication initiator [Francisella tularensis subsp. holarctica]	119	119	74%	8,00E-32	84%	Francisella philomiragia subsp. noatunensis strain 2005/50/F292-6C DnaA (dnaA) gene, partial cds	196	61%	7,00E-47	88%

>contig154340 length=154 numreads=8<br>CCACATCTCCATCGGAGACATCATCAACAGTCACTTTAAAACCATGACTATCTGC<br>CCAAC<br>GCATATACATACGCATCAGCATTTCAGCCCAATCTTGTGCTTCTGTACCTCCAGAA<br>CCTG<br>ACTGTaTTTCTAGAAACGCATTATTTGCATCCAT	peptide chain release factor 2 [Francisella tularensis subsp. tularensis NE061598] >ref|WP_014551162.1| peptide chain release factor 2 [Francisella tularensis] >gb|ADA77879.1| peptide chain release factor 2 [Francisella tularensis subsp. tularensis NE061598]	87.4	87.4	62%	2,00E-19	98%	rancisella philomiragia subsp. noatunensis strain 2005/50/F292-6C PrfB (prfB) gene, partial cds	171	79%	3,00E-39	87%
>contig27632 length=704 numreads=67<br>AGATAATATATGGTAGGGGAGCGTTCTGTAAGCCGATGAAGGTGTGTTGAGAA<br>GCATGCT<br>GGAGGTATCAGAAGTGCGAATGCTGACATGAGTAACGTAAAATAAGTGAGATT<br>CTTATTG<br>GCCGAATACCCAAGGATTCCTACGCAATGTTAATCAACGTAGGGTAAGCCGGCA<br>CCTAAG<br>GCGTAGCTGAAGAGTGAAGTCGATGGAAAACAGGTTAATATTCCTGTGCTGCTT<br>ATATGA<br>ATGATGGAGGGACGGAGAAGGCTAGGTAGGCCCGGCGAATGGTTGTCCGGGT<br>GAAAGTAA<br>GTAGGTAGATATGCTAGGTAAATCCGGTGTATTGTTGATCTGAGATACGAGACG<br>AAGTCA<br>AACTTGTTTGACGAAGCTATTGATGCCATGCTTCCAGgAAAATCTTCTAAGTATAT<br>TGTA<br>TAGGTAACCGTACTGTAAACCGACACTGGTGGGTAGGTAGAGAATACTAAGGCT<br>ATGAGA<br>CAACTCTGGTGAAGGAACTAGGCAAAATAACACCGTAACTTTGGAAGAAGGTGT<br>GCCCAT<br>GTTATGTGAAGGGACTTGCTCCTGGAGCAGAGATGGGTTGCAATTATCAGGTGG<br>CTGCGA<br>CTGTTTATCAAAAACACAGCACTCTGCGAAATCGTAAGATGAAGTATAGGGTGT<br>GACGCC<br>TGCCCGGTGCTGGAAGGTTAATTGAAGTGGTTAGCGCAAGCGAA							Francisella philomiragia subsp. philomiragia ATCC 25017 23S ribosomal RNA gene, partial sequence	1057	93%	0.0	94%
>contig52227 length=353<br>numreads=13CTTCATTTACCAAAAAGTTTGCAATTTCCATTTTCATTTTTGAAAA<br>AGgAATAGAAACCA<br>CTTCCTTCTGTGCAGAAAGACCGTTTCTCACTCTTGTAAACATATCCGCTATAGGA<br>TCTT<br>GCATACTCATAACTTAAATTCCTTATTACCAACTAGCTTTTTTCAAACCAGGAACA<br>TCAC<br>CTGCCATTGCATACTCTCTAAGTTTATTACGGCATAAGCCAAACTTTCTGTATACA<br>GCGT<br>GTGGTCTACCTGTAACTTTACATCTTCTTTGCACACGAGATGCTGAAGAGTTTACT<br>GGTA<br>GTTTTTGCAGCTTGATATTAGCTTCCCATACTTGTTCTTCAGTAGAATTTACA	30S ribosomal protein S14 [Francisella philomiragia subsp. philomiragia ATCC 25017]	143	143	52%	4,00E-41	97%	Francisella philomiragia subsp. philomiragia ATCC 25017, complete genome	473	90%	4,00E-130	91%
>contig117077 length=190 numreads=9<br>GCCAGGATATACATCCATATCTAAGCCATCTATAaGTAATTTCTCATCAAATGACT<br>TAAC<br>AAGATCTTTAACTTTGATGACATTGTTACCAAGTCTTTCACCAGGTGGGATATAT<br>AAGTC<br>TTGAGTTTCATTTCGTTTTTGGAACTCTTGAGAGCTTAACTCATCAAACTTAGCTA<br>ATCT<br>TGATTTAGAT	hypothetical protein [Francisella cf. novicida Fx1] >ref|WP_014550345.1| heme ABC transporter ATP-binding protein [Francisella novicida] >gb|AEE88185.1| ABC transporter, ATP-binding family protein [Francisella cf. novicida Fx1]	125	125	84%	2,00E-32	95%	Francisella philomiragia subsp. philomiragia ATCC 25017, complete genome	239	83%	9,00E-60	90%
>contig123706 length=183 numreads=6<br>TAAGATCTTCATCAAACTCAATAAGAGCTCTAGTTACACCAAGGCGAATTGCACC<br>AGCTT<br>GACCAGTAGTACCACCACCTTTAACTGTTACTTTGAAGTCAAAGTTTTCAGCATTT<br>TCAG<br>TAAGAACTAGAGGTTGCTTAACAATCATGCAATCAGTTTCACGACATAAATACTG<br>CTCTA<br>ATG	30S ribosomal protein S9 [Francisella philomiragia] >gb|EET21162.1| 30S ribosomal protein S9 [Francisella philomiragia subsp. philomiragia ATCC 25015]	84.3	84.3	85%	7,00E-19	93%	Francisella philomiragia subsp. philomiragia ATCC 25017, complete genome	209	86%	7,00E-51	87%
>contig142976 length=164 numreads=6<br>AGTGTAAACAACAAGCAAGGTTTGACTTTTGCTGCCGCATtAAAaTCATTCTTAag<br>ACAA<br>GATCCAGATATAATCATGGTTGGTGAGATCAGGGaTATCGAAACTGGATCTATT<br>GGTATT<br>AAAGCATCTCAAACAGGTCACTTAGTTATGTCAACGCTACATAC	type II secretion system protein/type IV-A pilus assembly ATPase PilB, partial [Francisella tularensis]	110	110	79%	2,00E-27	98%	Francisella philomiragia subsp. philomiragia ATCC 25017, complete genome	172	79%	9,00E-40	86%

>contig116307 length=191 numreads=8<br>AAATCTGTACACCAAGAGGCTTTTCCGAGTCCTCCATATAGAGCATttCCATAGTT<br>TCCC<br>AATGTCTATCTGCTACACCTTTGTTATTTACCATTTCAGTGTATATCAATCCAGCA<br>CCAT<br>GCTCTTtgCAAAGAGTCCTAAaTGCACTATCACAAAAGCCTGCCATAGGAGCTAG<br>CACTA<br>CATTATTTTCT	tRNA-dihydrouridine synthase [Francisella tularensis subsp. mediasiatica FSC147] >ref|WP_012429621.1| hypothetical protein [Francisella tularensis] >gb|ACD31045.1| tRNA-dihydrouridine synthase [Francisella tularensis subsp. mediasiatica FSC147]	124	124	84%	2,00E-32	87%	Francisella philomiragia subsp. philomiragia ATCC 25017, complete genome	159	85%	7,00E-36	82%
>contig46381 length=392 numreads=19<br>ATTTAGCTtATAAGTTTTGTCAACAAGTTGGTGATGAAGGTAAGGTTATTTTAAG<br>TggTA<br>TTAACTCATCAATGCTAGAAGTTGGTAAAGAAAAACTTACTAATAGAGGTTGTGT<br>TGGTA<br>ATATCGAaTATGTACAAGCAAATGCGGAAAGCTTGCCTTTTCCTGACAACTATTT<br>TGATT<br>GCATAACAATCTCTTTTGGCTTAAGAAATGTTACAGATAAGGCAAAAGCACTAG<br>CTTCGA<br>TATGTAGAGTATTAAAACCAGGTGGTAGGTTACTAGTGCTAGAGTTCTCTAAGC<br>CTATAG<br>TACCAATGCTTTCTAAAATATACGATGAATACTCATTTAAAGCTTTACCATTTATG<br>GGTA<br>AAATCATCACTCAAGATGCTGAAAGTTACAAA	Ubiquinone/menaquinone biosynthesis methyltransferase UbiE [Francisella sp. TX077308] >ref|WP_013922247.1| ubiquinone/menaquinone biosynthesis methyltransferase [Francisella sp. TX077308] >gb|AEI35399.1| Ubiquinone/menaquinone biosynthesis methyltransferase UbiE [Francisella sp. TX077308]	256	256	90%	4,00E-83	93%	Francisella philomiragia subsp. philomiragia ATCC 25017, complete genome	326	90%	1,00E-85	82%
>contig84512 length=241 numreads=10<br>ATACACCAAACTCAGTGATAGATCTGATTTTACCATGAACTTTATCACCAACTTTG<br>AAAT<br>TATCTTCAAAAGAATCCCAAGGGTTAGCTTTACATTGCTTAATACCTAGAGAAAT<br>TCTGT<br>GTTTCTGAGCATCTAACTCAAGTACGATAACTTCAACTTCTTGGCCAATTGATACA<br>GCTT<br>TGTGAGGGTTAACATTCTTGTTAGTCCAATCCATTTCAGATGTGTGAACAAGACC<br>TTCTA<br>T	30S ribosomal protein S1 [Francisella sp. TX077308] >ref|WP_013921941.1| 30S ribosomal protein S1 [Francisella sp. TX077308] >gb|AEI35080.1| SSU ribosomal protein S1p [Francisella sp. TX077308]	150	298	92%	4,00E-41	89%	Francisella philomiragia subsp. philomiragia ATCC 25017, complete genome	279	91%	6,00E-72	88%
>contig112895 length=195 numreads=10<br>GCTATAGCTAATGTCATGTTGTCTAGCTgCAGCATTAATACgAaCAATCCATAAAG<br>ATCT<br>AAATGTTCTTTTCTTATTTTTGCGATCTCTGTAAGCATATTGACCTGCTTTAATTAC<br>CGC<br>TTGTTTAGCTACTCTATATACTCTTGAACGAGCACCATAATAACCTTTTGCTTGTTT<br>TAA<br>GACTTTCTTATGTCT	50S ribosomal protein L20 [Francisella noatunensis subsp. orientalis str. Toba 04]	126	126	83%	3,00E-35	98%	Francisella philomiragia subsp. philomiragia ATCC 25017, complete genome	261	82%	2,00E-66	91%
>contig128384 length=178 numreads=6<br>ATAGCGGGATTGCTAGGAAATAAAGTTTGTTTAGATATTGATGATAGAAAAGCC<br>TATCCT<br>ATTCTTTGGGGTATGCTAATAGGGGATAGTGGAACAGGGAAAACCCCTAGTATC<br>AATGAA<br>CCTATGAAACCAATTAAAGAAATAGATAAACAGCTTTTAGATGATTATTTAAAAG<br>ACT	hypothetical protein [Francisella novicida] >gb|EDN38529.1| predicted protein [Francisella novicida GA99-3548]	119	119	88%	2,00E-30	93%	Francisella philomiragia subsp. philomiragia ATCC 25017, complete genome	257	89%	2,00E-65	93%
>contig170526 length=140 numreads=6<br>ATTGAAAAACTTCTAGAAATTGGAGTTGATAGAGTAGTTGTtGGTAGTTTAGCTG<br>TTAAG<br>GATACAGCATTAACGAAGAAGTTTTTTGAgAAaTATGGCGCTGAAAAGATAGTTT<br>TAGCT<br>TTAGATGTTTTTATCAAAGA	phosphoribosylformimino-5-aminoimidazole carboxamide ribotide isomerase [Francisella sp. TX077308]	85.9	85.9	76%	6,00E-19	98%	Francisella philomiragia subsp. philomiragia ATCC 25017, complete genome	191	76%	2,00E-45	91%
>contig146987 length=160 numreads=7<br>TTAACTATACAGCTTTTAAGTGATGAAGATCACAAAGTTGCTGAACTATTTGGTG<br>TATGG<br>ggTGAAAAGAAATTTAtGGgTAAAGTATATGATGGTATCCATAGAATTAGCTTTTT<br>TaTA<br>GACAAAGAAGGTAAGATTGATCATGTCTTTAATAAGTTTA	thioredoxin-dependent thiol peroxidase [Francisella philomiragia] >gb|EET20615.1| bacterioferritin comigratory protein [Francisella philomiragia subsp. philomiragia ATCC 25015]	106	106	86%	4,00E-27	93%	Francisella philomiragia subsp. philomiragia ATCC 25017, complete genome	211	84%	2,00E-51	91%
>contig73718 length=267 numreads=12<br>TTAGCGAAACGATTTTTTGTTTAGTTATCATACTGATTCCTATAGTACAGACTTAC<br>CATA<br>ATCTAGCTTTtCAACACCCATAAACTCAGCAATAGTTTGCCCTATATCTGCAAATG<br>TATC<br>TCTTGGGCCAACAAATTCTGATTCAATATTTTTACCCCACAATAAGAACGGTACG<br>CATTC<br>ACGTGTGTGGTCAGTACCAGGTGCAGTTGGATCACAACCATGGTCTGCTGCTAA<br>AATCAC<br>GATAGTATCCTCATCTAAATATTTATC	phosphopentomutase [Francisella philomiragia] >gb|EET20772.1| phosphopentomutase [Francisella philomiragia subsp. philomiragia ATCC 25015]	130	130	100%	2,00E-34	86%	Francisella philomiragia subsp. philomiragia ATCC 25017, complete genome	165	72%	2,00E-37	81%

>contig191739 length=118 numreads=6<br>CTCTTGCTCTACCTGTACCTTTCTGTCTCCAAGGTTTTGCACCACCACCAGACACT<br>TCTG<br>ATCTAGTTTTTTGCGCTTTAGTTCCTTGACGAGCACCtgCCATATAGGCAACTACA<br>AC	50S ribosomal protein L4 [Francisella noatunensis subsp. orientalis str. Toba 04]	80.9	80.9	75%	2,00E-17	100%	Francisella philomiragia subsp. philomiragia ATCC 25017, complete genome	169	75%	8,00E-39	92%
>contig97494 length=217 numreads=8<br>CCGACAATAGCTTCGTTAAAATGCACTTTAAATTTCGTTTAAAAGATGGAACCAT<br>AGCAG<br>AGGATACTGAAAATTATAACAGACCTTTTGTATTTCAAATGGGTCAAGGATGCTT<br>TACCG<br>ATAAGGTTGAAAATGAACTTATAGGTTCTGAAATTGGTGACAGTAAACGAGTCG<br>TGTTAA<br>TGCCAGATGAAGCTTTTGGTGAAAAACATCCTTCTAG	peptidyl-prolyl cis-trans isomerase [Francisella philomiragia] >gb|EET20256.1| peptidyl-prolyl cis-trans isomerase [Francisella philomiragia subsp. philomiragia ATCC 25015]	139	139	87%	1,00E-39	88%	Francisella philomiragia subsp. philomiragia ATCC 25017, complete genome	193	83%	8,00E-46	84%
>contig87198 length=236 numreads=8<br>AAAAGAATTTGTATAGCAGGAATGAATGGTTATACCGGCCAGATTTTAAAGAAA<br>CTGATA<br>AATAATCATCCAAATTTTGAGCTAGTTGGAAGCTTAGGTCTATCATCAAATAAAA<br>AAGAT<br>CAATATAGATATTTTTTGGATGACTTAAATAGTGAAAATTTCGATGTGGATTTTTT<br>ATTA<br>TTAGCTACACCTGCAGATATTTCTATAGAAATCGTAGATAAGATCAATCAAAAGA<br>A	N-acetyl-gamma-glutamyl-phosphate reductase [Francisella philomiragia subsp. philomiragia ATCC 25017] >ref|WP_012280023.1| N-acetyl-gamma-glutamyl-phosphate reductase [Francisella philomiragia] >gb|ABZ86774.1| N-acetyl-gamma-glutamyl-phosphate reductase [Francisella philomiragia subsp. philomiragia ATCC 25017]	115	115	79%	8,00E-29	68%	Francisella philomiragia subsp. philomiragia ATCC 25017, complete genome	161	70%	3,00E-36	81%
>contig136666 length=169 numreads=7<br>TTttGATTATAGTATCATCTATAGCTCCACGATCTATAGTTGTACCAGCaCCAACCT<br>CAA<br>CATCATCATGTATCACAACACGGCCAAGCTGTGGAATTTTTGTCCAACTACCATC<br>ATCAT<br>CTCtGgCATTACCAAAACCaTCACAACCAATTACTGAGTTTTGATGAAT	UDP-3-O-[3-hydroxymyristoyl] glucosamine N-acyltransferase [Francisella sp. TX077308] >ref|WP_013922561.1| UDP-3-O-(3-hydroxymyristoyl) glucosamine N-acyltransferase [Francisella sp. TX077308] >gb|AEI35721.1| UDP-3-O-[3-hydroxymyristoyl] glucosamine N-acyltransferase [Francisella sp. TX077308]	110	110	80%	2,00E-27	91%	Francisella philomiragia subsp. philomiragia	132	79%	1,00E-27	81%
>contig122027 length=185 numreads=9<br>CTATGTGGTTATTAGAAAATATTGATATGAAGAAATCTAAGTACATCCTGCCTAG<br>CTTGT<br>TTACCAGTGCAAGCCTACTTTTTGCTTTCCTAGCAATTATCTCAGCTTTTCAATCAc<br>ACT<br>TTGTATCTTGTGCTGTTTATATGCTTTTAGCTGGATTTGCTGATGCTTTTGATGGC<br>AAGG<br>TTGCT	CDP-alcohol phosphatidyltransferase [Francisella philomiragia subsp. philomiragia ATCC 25017] >ref|WP_012279958.1| CDP-alcohol phosphatidyltransferase [Francisella philomiragia] >gb|ABZ86691.1| CDP-alcohol phosphatidyltransferase [Francisella philomiragia subsp. philomiragia ATCC 25017]	75.5	75.5	79%	9,00E-15	90%	Francisella philomiragia subsp. philomiragia ATCC 25017, complete genome	128	75%	2,00E-26	80%
>contig157653 length=150 numreads=6<br>CTTTACCTGGTGCAAGTGTAATACCACTAccttGTAGTAAAGTAATAGCTTCATTA<br>GTCA<br>CAGGCATGTTAGATGCTTCAACAACATATTTAACACCGTTTGCTATTAATTGCTCT<br>GCTT<br>CTTTTTCACAAATTTCATTTTGTGTTGCAG	NADP-specific glutamate dehydrogenase [Francisella sp. TX077308] >ref|WP_013922474.1| glutamate dehydrogenase [Francisella sp. TX077308] >gb|AEI35633.1| NADP-specific glutamate dehydrogenase [Francisella sp. TX077308]	79.0	79.0	81%	1,00E-15	82%	Francisella philomiragia subsp. philomiragia ATCC 25017, complete genome	82.4	29%	1,00E-12	94%
>contig95932 length=220 numreads=7<br>CATCTTTTTCTTTAACAAGTATCTTCTTAAATGTAGTTCCAGGAGTTTCAGGTATC<br>ATCT<br>TGGCAATCATATCACCAAAGCCATAACCTTGCCCCATCACACGCACTACATCTCG<br>TACTA<br>CACCCTTAGCTGCCATAGTACCGTAAGTGATAATCTGTCCAACACTTTGCTTACC<br>GTACT<br>TCTGCTCTACATATTTGATAACTCTATCACGACCCTCTAT	DNA-directed DNA polymerase [Francisella philomiragia subsp. philomiragia ATCC 25017] >ref|WP_012279847.1| DNA polymerase III subunit alpha [Francisella philomiragia] >gb|ABZ86566.1| DNA-directed DNA polymerase [Francisella philomiragia subsp. philomiragia ATCC 25017]	132	132	77%	2,00E-33	93%	Francisella philomiragia subsp. philomiragia ATCC 25017, complete genome	211	73%	2,00E-51	87%
>contig146290 length=159 numreads=6<br>GCTAAAACTATTAATCTTATGAGTAATGCAATGAtCTAtgCTTATAATGACCGAAA<br>CTCA<br>GATCTTGGAGATCCAGATTTTGTGAAAATGGATATTAATaGgTTCTTGTCTAAAA<br>AGTAT<br>GCAAAACAAGTAGCAGAAAAAATAACGACAGATAAACAT	gamma-glutamyltransferase [Francisella novicida] >gb|EDX19567.1| gamma-glutamyltransferase [Francisella tularensis subsp. novicida FTE]	104	104	79%	2,00E-24	89%	Francisella philomiragia subsp. philomiragia ATCC 25017, complete genome	156	77%	9,00E-35	84%
>contig140924 length=166 numreads=7<br>AAAGAAGTTGATGGCGTAATGCACGAACCATTtGAGCAAGCTATTATTGATGTT<br>GATGAC<br>ACTTTCCAAGGTGTTGTGATGGAGAGAATGGGTCTGCGTCAAGGTGAGCTTAAA<br>AATATG<br>GAACCTGATGGTAAAGGTCGTGTAAAACTTGAGTTTATAATTCCAT	GTP-binding protein TypA [Fangia hongkongensis and Francisella philomiragia subsp. philomiragia ATCC 25017]	97.4	97.4	85%	7,00E-22	82%	Francisella philomiragia subsp. philomiragia ATCC 25017, complete genome	141	86%	2,00E-30	82%

Contigs	Blastx	Max score	Score	Query Cover	e-value	Identity	Blastn	Score	Query Cover	e-value	Identity
>contig43527 length=417 numreads=24<br>TATTACAATAaaTGGTGAGAAAAAAGACATTGAGTCATTTAACTCTGTGTATATG<br>ATGGC<br>TAATTCTGGTGCAaGGGgTTCTTATAACCAAATGCGTCAGTTAGCAGGTATGCGT<br>GGATT<br>GATGGCTAAACCAGATGGTACGATGATCGAGACTGCTATTACTGCAAACTTTAG<br>AgAAGG<br>CTTGTCAGTACTTCAATACTTTACATCTACGCATGGTGCTCGTAAAGGTCTTGCCG<br>ATAC<br>AGCTCTTAAGACTGCTAACGCAGGTTACTTAACGCGTAGACTAGTAGATGTAGC<br>TCAAGA<br>TTTAGTTATCATCGAAGAAGATTGTGGTACAGAAGACGGACTTATGTTCTCAGCT<br>ATTGT<br>TGAAGACGGTGAAGTTAAAGTACCTCTAGCTGAAAGAGCGTTAGGTAGAACTTT<br>AGC	DNA-directed RNA polymerase subunit beta' [Francisella cf. novicida 3523] >ref|WP_014548914.1| DNA-directed RNA polymerase subunit beta' [Francisella novicida] >gb|AEE26935.1| DNA-directed RNA polymerase beta' subunit [Francisella cf. novicida 3523]	275	275	91%	8,00E-83	96%	Francisella philomiragia subsp. philomiragia ATCC 25017, complete genome	446	90%	1,00E-121	86%
**Contigs**	**Blastx**	**Max score**	**Score**	**Query Cover**	**e-value**	**Identity**	**Blastn**	**Score**	**Query Cover**	**e-value**	**Identity**
>contig79157 length=253 numreads=13<br>CTTCAGGATCATGAGTTAAAACTATTTGTCGACCTAGTTTCTCAAAGGCAtATTTT<br>ACAG<br>AGGCAAAATTACTACCACAACAATCTATTATTGCTATCATAATaCCCcTTTTGAGC<br>TTGG<br>AATCTCACCTATACCATCTTTAGCAACTGCCTGTTTAAGAACTTTACCTAAACCCT<br>TAAA<br>GCAAGCTTCTATCTTATGATGCGTATTTTCACCAGTAACTTTGATATGTAGTGTTG<br>CCTT<br>TAGACCTTCAGCA	imidazole glycerol-phosphate dehydratase/histidinol phosphatase [Francisella philomiragia subsp. philomiragia ATCC 25017] >ref|WP_012279625.1| imidazoleglycerol-phosphate dehydratase [Francisella philomiragia] >gb|ABZ86286.1| Histidinol-phosphatase [Francisella philomiragia subsp. philomiragia ATCC 25017]	96.7	96.7	49%	8,00E-22	92%	Francisella philomiragia subsp. philomiragia ATCC 25017, complete genome	274	81%	4,00E-70	86%
>contig180890 length=130 numreads=6<br>TGTCAGCATAGATGAAATTATTCTACTATGATGATAATTCACATCTTTTTCTCTAT<br>AAGC<br>TTCTATTTCTTGCTCAGTCGGAAGATCTTTAATTTCTGGTAACGAGCTCTTGTAAG<br>GAGG<br>ATTAACTTTC	hypothetical protein Fphi_1895 [Francisella philomiragia subsp. philomiragia ATCC 25017] >ref|WP_012281160.1| hypothetical protein [Francisella philomiragia] >gb|ABZ88119.1| conserved hypothetical protein [Francisella philomiragia subsp. philomiragia ATCC 25017]	89.0	89.0	77%	5,00E-19	98%	Francisella philomiragia subsp. philomiragia ATCC 25017, complete genome	226	78%	5,00E-56	98%
>contig61537 length=308 numreads=11<br>TTCAGCTAAAGATTTTGAAGCATGCACACAAGCATAATCAAACTCTATACCTTGA<br>CCAAT<br>ACGATTTGGCCCTCCGCCAAGTATTATTACTTTTTTGTTATCAGTAATATCAGATT<br>CACA<br>ATCTCTTATGATCTCTTCATGAGAAAAATGCTCATAAGTTGAGTACATATATGAA<br>GATGA<br>AGAATCAAACTCAGCTGCACAAGTATCAaCCTTCTTATATGTTGGATAAATCTCTT<br>TACC<br>ATAACGATAGTTACGTACATATTTTTCTGTTCTACCAACTAGCTTTGCAATTCTTG<br>CATC<br>AGAAAAAC	carbamoyl phosphate synthase large subunit [Francisella philomiragia] >gb|EET20567.1| carbamoyl-phosphate synthase large chain [Francisella philomiragia subsp. philomiragia ATCC 25015]	191	231	84%	7,00E-54	82%	Francisella philomiragia subsp. philomiragia ATCC 25017, complete genome	198	82%	3,00E-47	79%
>contig117499 length=190 numreads=7<br>CCACTCTATCTGTCATCAAGATTACTTCGATACCTTTTTTCTTGAAGACTTCAAGCT<br>GAG<br>GGTTGTTTAAAGCTGCTTTATAGCTATCTGCTGTGATGTAGTAGATAGTATCCTG<br>CTCTT<br>CTTTCATACGAGAGATATAGTCTGCTAAAGAAACTGTTTGCTTAGCATCAGTACT<br>CTCTG<br>TAGTTGTAAA	heat shock protein 90 [Francisella noatunensis subsp. orientalis str. Toba 04]	118	118	82%	4,00E-29	89%	Francisella philomiragia subsp. philomiragia ATCC 25017, complete genome	195	78%	2,00E-46	86%
>contig70644 length=276 numreads=13<br>CAGTATGATCTCCTTGGATGTTAGTGTTATCTGTTATGTATGCACCTAATCCTGAC<br>GCTT<br>GAATTATAGAGTTTTTACCCCAATTAATATATTCACAAACGATACTAGCATTCCAA<br>GCAC<br>CACCAGCAGCAGTAATTGCACCAGTTACATAGTATGGCATTACAGCTGGGAATA<br>AGAATT<br>TTTTCCATTTTGTGAAACCTTTTAGCTGCATATTACTCGCCGCAAGCTTTAGCTCA<br>TCTG<br>GAATTGCTGAAGCACcGGCAATCACATTGAATAATA	ABC transporter permease [Francisella sp. TX077308] >ref|WP_013921923.1| ABC transporter permease [Francisella sp. TX077308] >gb|AEI35062.1| ABC transporter, permease protein [Francisella sp. TX077308]	166	166	91%	4,00E-46	87%	Francisella philomiragia subsp. philomiragia ATCC 25017, complete genome	180	66%	8,00E-42	83%
>contig132469 length=174 numreads=6<br>AAAGCTCTAGCAATGTTAATAGAGTGTCAGACTTCTGGAAGcAAaCTGGTGAGTT<br>GATGG<br>ATGAGATCAATGTAATGAACTATGATTACCAAGGTGGTTGGGGTACAGGTTTCC<br>CTGCAT<br>ATTTCCAGGCATCTACAACTTTCCCTGAGCAAACACCATACTCAAATAGAGTAT	glycosyl hydrolase family chitinase [Francisella philomiragia subsp. philomiragia ATCC 25017]	97.8	97.8	75%	7,00E-22	90%	Francisella philomiragia subsp. philomiragia ATCC 25017, complete genome	152	81%	1,00E-33	84%

>contig100652 length=212 numreads=10<br>TTTTAGCTATATCTGCCATAGTTATGCCTAAAATACCTGCTTTTTCTCTATCTATGTCAA<br>TATCAACAACTGGGTTGTCAAACTTAAGATCACTTTGaGCGAAAACAAACATACCACTGC<br>tCATCATTTTATCTATCAGCTTATTTGTGGTTTCGTTGATAGCTTCATAGTCATTAACAC<br>TTTGAATAACAAGAGACATTGGTGCACCACGA	multidrug transporter AcrB [Francisella philomiragia] >gb|EET20764.1| RND efflux transporter [Francisella philomiragia subsp. philomiragia ATCC 25015]	134	134	86%	4,00E-34	88%	Francisella philomiragia subsp. philomiragia ATCC 25017, complete genome	147	76%	7,00E-32	81%
>contig67462 length=286 numreads=14<br>CTTTGTAATTACAGAAGACTTTACTCATTTAAACAAATCAAAACCAGTTGTACTAGCAGC<br>AGGGgtAATCTGGGCATTAGTCGCTATAGTCGGCAAGACAACTCATGCAGGACATATAGT<br>CGCTGCTAATTTTGATCATATCATGGTTGAGTATGGTGAGCTTTTATTATTCTTAATGGT<br>CGCGATGGCATACATCAATCTTATGGAAGATCgTAATGTATTTGAAAAACTCAAAAGCAT<br>ACTAGTCCGAGCAGGGTTTAGTTATCTTGGAGTTTTTTGGCTTACA	sodium:proton antiporter [Francisella philomiragia] >gb|EET20661.1| Na+:H+ antiporter [Francisella philomiragia subsp. philomiragia ATCC 25015]	156	156	88%	5,00E-43	79%	Francisella philomiragia subsp. philomiragia ATCC 25017, complete genome	145	88%	3,00E-31	76%
>contig125719 length=181 numreads=8<br>CGATAACTTGTATATGAGGGAAGTTCTCTTTCACCCATTCAACCATATCTAGCACACCTT<br>GAGAATGACCATGAGCAGTAtcAACTACAACCACATCAACACCTTCTCTTGCAAGAGCTG<br>TAACTCTTTCTTTAGTATTTGCAGCAGTACCAACAGCAGCACCGACTCTCAAGCGACCAA<br>G	Inosine-5'-monophosphate dehydrogenase [Francisella cf. novicida 3523]	113	113	84%	6,00E-28	90%	Francisella philomiragia subsp. philomiragia ATCC 25017, complete genome	141	71%	3,00E-30	84%
>contig148374 length=157 numreads=7<br>AGCGTATGGTGTTGACATAAAGATAACCCTGgTATTTGGCATGATAAACAATACCAAAGC<br>AATATATTGgATTTATCTACAGCATTGGGTGAGCTAGATATGTGGACAAGATTGCATTAT<br>GTATATCCATACCCTCATGTTGATAAAATTGTACCAC	ribosomal protein S12p Asp88 methylthiotransferase [Francisella sp. TX077308] >ref|WP_013922163.1| ribosomal protein S12 methylthiotransferase [Francisella sp. TX077308] >gb|AEI35313.1| Ribosomal protein S12p Asp88 methylthiotransferase [Francisella sp. TX077308]	90.9	90.9	61%	6,00E-20	93%	Francisella philomiragia subsp. philomiragia ATCC 25017, complete genome	130	73%	5,00E-27	82%
>contig167076 length=143 numreads=7<br>TTTGCGCCTTTTCACCCAGTCTGATGTAGCTGTTGGGCAAAACTATTATGAACAATTTAT<br>TCCTGCTTTTAAAAATAATGAAGTACGCAATATGCTTGGTTCATTTGCTGCTCGTGAAGG<br>TATTCATCAAAGAGCTTACGCCC	ribonucleotide reductase of class Ia (aerobic) subunit beta [Francisella sp. TX077308] >ref|WP_013923022.1| ribonucleoside-diphosphate reductase subunit beta [Francisella sp. TX077308] >gb|AEI36188.1| Ribonucleotide reductase of class Ia (aerobic), beta subunit [Francisella sp. TX077308]	99.8	99.8	77%	2,00E-23	98%	Francisella philomiragia subsp. philomiragia ATCC 25017, complete genome	126	72%	6,00E-26	84%
>contig140594 length=165 numreads=8<br>TTCAGGTGGCTATCATAAGGcAGGTTTTGAaGGTGGTCAAATGCCTTTGCAAAGAAGATT<br>ACCAAAATTTGGTTTTACTTCACCAACTAAAAGATTTGCAGCTGAAGTTAGGCTACATGA<br>ATTAAATAATGTAGATGCTGATGTTGTAACAATTGATGTGCTTAA	50S ribosomal protein L15 [Francisella novicida U112]	99.4	99.4	87%	1,00E-24	81%	Francisella philomiragia subsp. philomiragia ATCC 25017, complete genome	124	68%	2,00E-25	84%
>contig174035 length=137 numreads=6<br>ATGAATTAAAAACTACTAAAGAAGATTATGAGCTAGAAAaTATAGagCTtgTATATGGTG<br>GTCAACAAGATATTGAACAAGCTACTAAAATTGGTTTAGCAATTGCTTGTGGTCAAAATT<br>ATGCTAAAGATTTACAA	hypothetical protein [Francisella cf. novicida Fx1] >ref|WP_014549578.1| cytochrome C oxidase subunit II [Francisella novicida] >gb|AEB27615.1| Cytosol aminopeptidase PepA [Francisella cf. novicida Fx1]	72.8	72.8	83%	2,00E-13	76%	Francisella philomiragia subsp. philomiragia ATCC 25017, complete genome	89.8	45%	7,00E-15	88%
>contig166790 length=143 numreads=6<br>AGTTGTTGATAAGATCAAGGGTATTAAAGTAGAACATGAGGTCCTTGTTGGTAATCCTGC<br>AGCAGAGGTTGTAGCGTATGCAGAAGAAAGCGCATGTGATGTAATTGTGCTAGGTTCTCA<br>TGCAACTCATGGTATAAATTTAC	universal stress protein [Francisella tularensis subsp. holarctica PHIT-FT049]	51.6	51.6	59%	3,00E-06	92%	Francisella philomiragia subsp. philomiragia ATCC 25017, complete genome	86.1	66%	1,00E-13	79%
>contig124775 length=180 numreads=6<br>tAATCAAAGGAGAGATATAAGTGAGATGATAAAATCAGGGGTTTAAAGGTTTGGGTTACG<br>AGTTAAGGATATTATGTTTTATTGATTTGTCCTCTAaGATATTAACTAGCCTTATAAAAC<br>TTAGGTTCAAATTCTACACCGTATTTTACATTCATCCACATTCTACTGATAAaaCTTTGC	putrescine/spermidine ABC transporter substrate-binding protein [Francisella philomiragia] >gb|EET21885.1| putrescine-binding periplasmic protein [Francisella philomiragia subsp. philomiragia ATCC 25015]	49.3	49.3	32%	5,00E-05	80%	Francisella philomiragia subsp. philomiragia ATCC 25017, complete genome	75.0	30%	3,00E-10	86%

>contig172525 length=138 numreads=11<br>TTACTCTATAGTTACTAACTATAGCCTATATGGTtCTTCAAAATCAATTCTTAACCT<br>TTT<br>TATAAAAATACCTAGGATTTATCGTATCTGCTAAAAGCTTATTTTCAAAGTCTATT<br>CTGA<br>GCAATATCCCGATCGTAT	cell division protein FtsW [Francisella novicida] >gb|EDX19776.1| cell division protein FtsW [Francisella tularensis subsp. novicida FTE]	55.8	55.8	54%	1,00E-07	83%	Francisella philomiragia subsp. philomiragia ATCC 25017, complete genome	71.3	35%	2,00E-09	89%
>contig188530 length=121 numreads=7<br>AAAGGAAATACGAAAAATAGAAATAAACTTTAACTGTTGTTTCTCAAGTGATTTA<br>ACTTA<br>GTGTTTGTAGATAATATATTTTAGTGTAAATAAATaCGGGTCTGTAGCTCAGCTG<br>GTTAG<br>A							Francisella sp. GP-2009 16S rRNA gene (partial), tRNA-Ile gene, tRNA-Ala gene and 23S rRNA gene (partial)	167	92%	2,00E-38	97%
>contig69877 length=279 numreads=32<br>TGTGGATCGGAGTGAAAGGCTAATCAAGCACGGAGATAGCTGGTTCTCCCCGA<br>AAACTAT<br>TTAGGTAGTGCCTCGTGTATAACTGATTGGGGTAAAGCACTGTTTCGACTAGGG<br>GGGTTT<br>TACGACCTTACCGACTCGATGCAAACTCAGAATACGATCAAGTTCAATCACGGG<br>AGACAC<br>ACTGCGGGTGCTAAGGTCCGTAGTGGAGAGGGAAACAGCCCAGACCGCCAACT<br>AAGGTCC<br>CCAAGTCATaGcTAAGTGGGAAACGAaGTGGGAAGGCCC							Francisella sp. GP-2009 16S rRNA gene (partial), tRNA-Ile gene, tRNA-Ala gene and 23S rRNA gene (partial)	167	97%	2,00E-38	92%
>contig65400 length=293 numreads=26<br>GCTATCTCCGTGCTTGATTAGCCTTTCACTCCGATCCAcAACTCATCCcGTAATTTt<br>GCA<br>ACATtAGTGGGTTCGGTCCTCCAGTTAGTATTACCTAACCTTCAACCTGGTCATGG<br>ATAG<br>ATCGCGCCGGTTTCGGGTCTACTCCTAGCAACTATACgCCCTATTAAGACTCGCTT<br>TCGC<br>TACGGATCCCTTATTCAGTTATCCTTGCTACTAAAAGTAACTCgCTGACCCATTAT<br>ACAA<br>AAGGTACGCAGTCACATAACTAAATTATGCTCCTACTGCTTGTATGCAAGTGG							Francisella sp. TX077308, ribosomal rRNA	1449	83%	6,00E-133	98%
>contig42998 length=422 numreads=20<br>AAAAGTTTGGCAAGACcATTTAGATAAACATCTAACTTTAGAACAAGAAAAAGA<br>TTTTCC<br>AAATATCCCACTTATTGAATTAAGTAAATTTGCTGTAgaCCCTGAGAAGATATTTG<br>ACAC<br>TACAGTTTATCTTTCAAAAGAtACTCATGATACAATATCATCAGCTACTAGCACTT<br>CTGG<br>CTGGGGTTGGCGATATCCTGGTAGGCTTGGAGATAGCCCTATAATCGGAGCTGg<br>CTCGTA<br>TGCAGACTCTCGTTATGGTGCCTGTGCTTGTACTCATACTGGTGAGATGGCTaTtC<br>GCTG<br>TGGTACCGCTCGCTCTGTGgTTttATATATGAAAATGGGAATGTCTGTAAAAGAA<br>GCTGT<br>ACTTGAAGCTGCTAAAGATTTACGCCATCTAAAAACTGGTTACTTAGATGAGCTA<br>ACTAT<br>TC	N(4)-(beta-N-acetylglucosaminyl)-L-asparaginase [Francisella sp. TX077308] >ref|WP_013923506.1| asparaginase [Francisella sp. TX077308] >gb|AEI36677.1| N(4)-(Beta-N-acetylglucosaminyl)-L-asparaginase precursor [Francisella sp. TX077308]	274	274	92%	1,00E-89	93%	Francisella sp. TX077308, complete genome	1410	98%	4,00E-129	96%
>contig90118 length=229 numreads=9<br>CATCACACCACCCAtAGTCTCAATACCTACAGATAGAGGAGTTACATCAAGAAGC<br>AATAC<br>ATCTTTAACATCTCCTGCTAATACACCACCTTGAATAGCTGCACCAACTGCCACAG<br>CTTC<br>ATCAGGGTttACATCTTtACGCGGCTCTTTACCAAAGAACTCTTTTACTTTTTCTTG<br>TAC<br>TAGAGGCATACGAGTTTGTCcACCTACTAAAAGCACTTCTGTAATATCA	molecular chaperone DnaK [Francisella sp. TX077308] >ref|WP_013922807.1| molecular chaperone DnaK [Francisella sp. TX077308] >gb|AEI35971.1| Chaperone protein DnaK [Francisella sp. TX077308]	117	117	90%	8,00E-29	99%	Francisella sp. TX077308, complete genome	315	91%	2,00E-82	91%
>contig55868 length=334 numreads=22<br>TCCTtcAAGATTAGATATCCCAGGTCTTAGACCTGTAAGGAACGTTGGAGACTAC<br>GACGT<br>TGATAGGCTGGGTGTGGAAGTACAGCAATGTATGAAGCTTACCAGTACTAATGA<br>TCCGAG<br>AGACTTAAGTCTATTTCTATGCTGAATTGTTAAATATCTTTATTGATATGCAATTA<br>AACA<br>GTAAAATACAAAACCAAAaCATTAGAGATTAAAGTTTTGGCGATGATAGCTTGT<br>AGGTAC<br>CACCTGATCCCATTCCGAACTCAGAAGTGAAACTATAACACGCCgaTGATAGTTT<br>AGCAT<br>TCGCTAAGTGAAAGTAGGTAGTCGCCATTTTATT	Francisella sp. TX0773083-dehydroquinato synthase and 23S ribosomal RNA gene (by blastn)	43.1	43.1	27%	0.001	57%	Francisella sp. TX077308, complete genome	996	88%	2,00E-78	84%

>contig95299 length=221 numreads=8<br>CCTGCTAAGATAACAAGAGGAATACCTTTTTCTTTATACTTCATAGCAGCATCAA<br>ATACA<br>TACTGTTGTGAACCATCAAAATGATACTTAGTATACCCGCCTTCTACACCGTCAA<br>GAAGT<br>AAGTTACGGATACGAATATTTGCAAATGTACCACGCATCATCACTTCATGGTTAC<br>CACGA<br>CGAGAACCGTAAGAGTTAAAGTCTTTTTCTCTACACCGTG	Aconitate hydratase [Francisella sp. TX077308] >ref|WP_013922367.1| aconitate hydratase [Francisella sp. TX077308] >gb|AEI35524.1| Aconitate hydratase [Francisella sp. TX077308]	155	155	91%	9,00E-42	97%	Francisella sp. TX077308, complete genome	287	86%	4,00E-74	91%
>contig171333 length=139 numreads=24<br>ATTCAGACAAGGTTTCTCGTGCCCCGCCcTACTTGTTCGTATgtCTAGTTCCACTAA<br>GAT<br>TATTTCGTATACGGGACTATCACCCTCTATCGTCAAGCTTCCCAACTTGTTCTACT<br>ATAA<br>TTCTAGCTATATCATACCA							Francisella sp. TX077308, ribosomal rRNA	623	75%	2,00E-50	94%
>contig159845 length=149 numreads=6<br>AAATATCTAAGAGTGAAGCAAAAATTACTCAAGCTAAAAAAGAGATGATTGAAG<br>CAAACT<br>TAAGATTGGTTGTTTCTGAAGCTAAAAAATATACAAACAGAGGTTTACACTTCTT<br>AGATA<br>TTATCCAAGAGGGTAATATTGGTCTAATG	RNA polymerase sigma-70 factor [Francisella noatunensis subsp. orientalis str. Toba 04]	100	100	77%	4,00E-23	98%	Francisella cf. novicida 3523, complete genome	182	74%	1,00E-42	89%
>contig161267 length=148 numreads=7<br>TTAAATTAGATAATATAATGGAAGTGCCGCGTATTGAGAAGATTACTCTTAATAT<br>GGGTG<br>TTGGTGATGCTGCAAAAGACAAAAAAGTTAtgACTTTTGCTGTAAATGATTTGAC<br>TGCAA<br>TtGCTGGTCAAAAGCCAGTTGTTACTAA	50S ribosomal protein L5 [Francisella sp. TX077308] >ref|WP_013922028.1| 50S ribosomal protein L5 [Francisella sp. TX077308] >gb|AEI35172.1| LSU ribosomal protein L5p (L11e) [Francisella sp. TX077308]	90.9	90.9	85%	3,00E-21	94%	Francisella sp. TX077308, complete genome	198	84%	1,00E-47	92%
>contig195973 length=111 numreads=6<br>CATGGGTAATAGCAaCTACTGTACCTTTGTACTCTGCTAAGAATTTCTCTAACCAT<br>GCTA<br>CAGATTCAGCATCTAAGTGGTTAGTAGGCTCATCTAGTAGTAGTATATCAG	ABC transporter ATP-binding family protein [Francisella sp. TX077308] >ref|WP_013923710.1| heme ABC transporter ATP-binding protein [Francisella sp. TX077308] >gb|AEI36885.1| ABC transporter, ATP-binding family protein [Francisella sp. TX077308]	76.3	113	75%	8,00E-15	100%	Francisella sp. TX077308, complete genome	163	78%	3,00E-37	93%
>contig85008 length=240 numreads=11<br>GGAAAGCTTCTTCTTTGTCCATACCATTATCAGTGAAGTAACGATAAAGCACTTC<br>GATAA<br>TTCCATGTACACCACCAAGTAAAACACCTCTTTCACCAAAGATATCAGAGCGATA<br>TTCCA<br>TTTCCATAGTTGTTGGGAATACAAATGGTGCGCCACAAGCTATAGCCCAGCCTA<br>GAGCTT<br>GTTCAGTTGCTTTACCGTTAATATCTTGATAAACACCAATACTACTATTGATACCA<br>GCAC	Ketol-acid reductoisomerase [Francisella sp. TX077308] >ref|WP_013923608.1| ketol-acid reductoisomerase [Francisella sp. TX077308] >gb|AEI36780.1| Ketol-acid reductoisomerase [Francisella sp. TX077308]	164	164	90%	3,00E-46	96%	Francisella sp. TX077308, complete genome	364	90%	2,00E-97	94%
>contig64191 length=298 numreads=15<br>TACGAAGATCTGTATCGATATTTACTTTACGCACACCGTACTTAATAGCTTCTACG<br>ATTT<br>CACTTACAGGTACGCCGTAAGTTTCACCCATAGAaCcACCGTAATTATTGATAACT<br>TCTA<br>ACCAGTCTTGAGGTACAGATGAAGAACCATGCATTACAaGGTGAGTGTTAGGGA<br>TTCTAG<br>CGTGAATCTCTTTTACTCTGCTTATAGATAAAACATCACCTGTAGGTGGCTTAGT<br>GAACT<br>TATAAGCACCGTGAGAAGTACCTATAGCAATTGCTAAAGCATCAACCTTAGTTTT<br>CTT	fructose-1,6-bisphosphate aldolase [Francisella philomiragia subsp. philomiragia ATCC 25017]	197	197	86%	1,00E-59	95%	Francisella sp. TX077308, complete genome	348	87%	2,00E-92	88%
>contig86933 length=236 numreads=14<br>TCATCATTACCAGGAATAATATAGTCAATACCTTCAGGGTTTGAGTTAGTATCTA<br>CAacA<br>GACACTACTTTAATACCTAATTTTTGAGCTtCTTGAATAGcGATGTGCTCTTTATTA<br>CTA<br>TCAATAACAACGATTGCATCAGGAATACCACCCATTTCTTTAATACCACCAAGAA<br>CTTTT<br>TCAAGCTTCTCGATAGTTCTCATGTTTTGTAGCATTTCTTTTTAGTTAAAGATTC	RecName: Full=30S ribosomal protein S2Francisella philomiragia subsp. philomiragia ATCC 25017	153	153	87%	3,00E-44	95%	Francisella sp. TX077308, complete genome	292	84%	9,00E-76	90%

<table>
<tr><td>>contig40455 length=449   numreads=28
ATGCAAGAAGTCTTCCATCTGTAAGAGCTTATCTTTCTCGCCTTCCATCATCTTAG
AAAC
TGGAATACCTGTAGCTTTTGAAACTACATCTGCAATCTCATTTTCTGTAACTGACG
TTCT
AACTAGCTTATTTTCACTTAAATCAGAAGCTGTCTCTTCTATCTGCTTAATCTGAG
CTTC
AAGCTCTGGAATTTTACCgTATTGCAGTTCTGACATCTTATTTAAGTCGCCTGCTC
TTTG
ATACTTTTCAAGCTCAAACTTAGCTTTCTCAAGTTTTTCTTTCAGCTTGCTTGTACC
TTG
CATTTTTAGCTTCTCTGCTTTCCAAATCTCCtCTAAGCCCTTGTACTCAGAATCCAG
ACC
TTTTATTTCAATCTCAAgtATCTTCAAACGCTTCTTCGTAGCATCATCTTTTTCTTTT
TT
AAGTTGCTCGCGCTGCATTTTTAGCTGGA</td><td>protein disaggregation chaperone [Francisella novicida] >gb|EDN38479.1| hypothetical protein FTDG_01296 [Francisella novicida GA99-3548]</td><td>214</td><td>214</td><td>93%</td><td>7,00E-62</td><td>91%</td><td>Francisella sp. TX077308, complete genome</td><td>287</td><td>92%</td><td>8,00E-74</td><td>78%</td></tr>
<tr><td>>contig55745 length=334   numreads=11
CCTATAACACCCTTTTCAaCTACTGTCTGAATAGCATCACAGTGATCTGTCACATA
TAAC
CAGTCACGGATATTTGAACCATCTCCATATACAGGAATAAGTTTTTGATTAATAC
AACTT
TTGATAACTACAGGTATTAGCTTCTCTGGATGCTGATATGGACCATAGTTGTTTG
AACAG
TTAGATATAGTTACAGGTAGCTTATAAGTaTGTAAATAGGCTCTTACTATATGGT
CAGAA
CCTGCTTTACTAGCTGAGTATGGTGAATTTGGTTCATAAGCCTTATCTTCTGTAAA
TGCT
GGATCATCTTTATCAAGAGTACCATAAACCTCAT</td><td>dTDP-glucose 4,6-dehydratase [Francisella sp. TX077308] >ref|WP_013922615.1| dTDP-glucose 4,6-dehydratase [Francisella sp. TX077308] >gb|AEI35776.1| dTDP-glucose 4,6-dehydratase [Francisella sp. TX077308]</td><td>217</td><td>217</td><td>93%</td><td>2,00E-67</td><td>94%</td><td>Francisella sp. TX077308, complete genome</td><td>276</td><td>93%</td><td>1,00E-70</td><td>82%</td></tr>
<tr><td>>contig97732 length=217   numreads=7
ATTGATTAGGTTTTTAATTGTTGGTTTtGGAGGCACGGTTAAGCCATTTTTGATAT
CTGC
ATGACGATTACCGAGCATTTGTAAATCAGCAGTCAGTACTAATgcACTACAACCA
GCATG
CTTTGCACTTGCTATGAGATTTGCCATAAATTTTCTATCTTTCATCATATACAGCTG
GAA
CCAAAATGGCTTGGTTGTGTGTTTTGCAACTTCTTCA</td><td>L-lactate dehydrogenase, partial [Francisella tularensis]</td><td>151</td><td>151</td><td>87%</td><td>1,00E-42</td><td>97%</td><td>Francisella sp. TX077308, complete genome</td><td>248</td><td>85%</td><td>2,00E-62</td><td>88%</td></tr>
<tr><td>>contig93736 length=222   numreads=9
CACGCTTacTAaaGTtttAGAGAATATCCTTGCTTTAGCACATCCGCTTATTCCATTT
AT
TACAGAGAGTATTTATCAGCAGTTAAAAGCTCATCTTGATAaTGCTCAAACTTCA
ATTAT
GGAtGTTAGCTACCCTGAAACTACTCAAGATTTAGAAGCTCCAGAGGCTGAGAA
AGCTAT
CAGATGGTTACAGAACATCGTAACAACTCTTAGAAATATGCG</td><td>valyl-tRNA synthetase [Francisella sp. TX077308] >ref|WP_013921990.1| valyl-tRNA synthetase [Francisella sp. TX077308] >gb|AEI35130.1| Valyl-tRNA synthetase [Francisella sp. TX077308]</td><td>132</td><td>132</td><td>89%</td><td>7,00E-34</td><td>88%</td><td>Francisella sp. TX077308, complete genome</td><td>237</td><td>88%</td><td>4,00E-59</td><td>86%</td></tr>
<tr><td>>contig133396 length=173   numreads=7
TTATCGAGAGAAATACGACTATACCTACTAAGAAATCACAAGTATTCTCTACAGC
TGAAG
ATAATCAGCCTGCAGTTACAATACATGTAATGCAAGGTGAGCGTGAAATGGCGT
CAGCAA
ATAAATCTTTAGGTAGATTTGACTTAGCTGATATTCCACCAGCACCTCGTGGT</td><td>molecular chaperone DnaK [Francisella tularensis subsp. mediasiatica FSC147]</td><td>122</td><td>122</td><td>87%</td><td>1,00E-30</td><td>97%</td><td>Francisella sp. TX077308, complete genome</td><td>226</td><td>86%</td><td>6,00E-56</td><td>90%</td></tr>
<tr><td>>contig111355 length=197   numreads=8
TTAATAGTAGCTAAGCCTGCTGCACAAGATACTGGGTGTCCACTATATGTAGCTC
CAAGC
ACGAATGCTGTATCTTGGAATTTAGCCATGaTTTTATCAGATAACATACAGCAAC
CTAGA
GGTAAATATGATGATGTAATACCTTTTGCCATTGTTACGATATCAGGCACGATAT
CATAA
TTCATAAAACCAAACCA</td><td>adenosylmethionine-8-amino-7-oxononanoate aminotransferase [Francisella sp. TX077308]</td><td>140</td><td>140</td><td>82%</td><td>7,00E-38</td><td>97%</td><td>Francisella sp. TX077308, complete genome</td><td>226</td><td>83%</td><td>8,00E-56</td><td>87%</td></tr>
<tr><td>>contig141672 length=165   numreads=8
TTCGTTTTGATTTTGGTGATATGGGTTGAATGAGCTGTAAACcATTCTTACGCTTT
TAGC
AGGTACGATAGTAGAAATAGCATGCTTATAAACCATTTGGTTTACAGTATTTCTG
AGTAC
GATACAAAATTGATCAAACGCTTCTACTTGACCTTGTAGTTTAAT</td><td>RNA-binding protein Hfq [Francisella sp. TX077308] >ref|WP_013923338.1| RNA-binding protein Hfq [Francisella sp. TX077308] >gb|AEI36506.1| RNA-binding protein Hfq [Francisella sp. TX077308]</td><td>114</td><td>114</td><td>89%</td><td>5,00E-31</td><td>96%</td><td>Francisella sp. TX077308, complete genome</td><td>213</td><td>86%</td><td>4,00E-52</td><td>91%</td></tr>
<tr><td>>contig129053 length=176   numreads=7
CTGCTGTTGGGGATATCATATATGTAAAACCATCACCAACTTGAAATGCTAATAT
CATAA
GCTGTCTTTTCATCTCTACCATATCTGCAACAGGAATCAAAATAGGCATAGTAGC
TAAAG
CTTGACCTGACCCACCAGGTATAAAGAAGTTTATAACGCTTTggaCTAGTGTCATT</td><td>Arginine/ornithine antiporter ArcD [Francisella sp. TX077308] >ref|WP_013922004.1| C4-dicarboxylate ABC transporter [Francisella sp. TX077308] >gb|AEI35144.1| Arginine/ornithine antiporter ArcD [Francisella sp. TX077308]</td><td>119</td><td>119</td><td>85%</td><td>4,00E-30</td><td>100%</td><td>Francisella sp. TX077308 complete genome</td><td>207</td><td>92%</td><td>2,00E-50</td><td>88%</td></tr>
</table>

>contig102038 length=210 numreads=11<br>CTGTAATTTTGCTAGACGAAGTTGAgAAAGCTCACGCAGATGTATTTAACATACT<br>TCTAC<br>AAGTATTAGATGATGGTCGTCTTACAGATGGACAGGGACGTACAGTTGATTTTA<br>AAAACA<br>CTGTAATAGTTATGACTTCAAACCTTGGTTCGCATAAGATTCAAGAAATGCAGGG<br>TGATG<br>ACTATGAAACTGTAAAATCTGCTGTGATGG	protein disaggregation chaperone [Francisella novicida] >gb|EDN38479.1| hypothetical protein FTDG_01296 [Francisella novicida GA99-3548]	140	140	87%	2,00E-36	96%	Francisella sp. TX077308, complete genome	207	87%	3,00E-50	85%
>contig119829 length=186 numreads=8<br>TTAAATTCATTATCAACTACATAAGGATTTTCccGACAGTAAATTCTAAACAGAGC<br>AGCA<br>TTACCTTCAACATGCATCGATTCATCACGAATAGACCATTCAACAACTTTACCCAT<br>ACCT<br>TTCATTTTGCCGAAACGTTGGAAGTTAAGAAGCATGGcAAATGATGCAAATAAT<br>GCAACA<br>CCTTCA	bifunctional gluaredoxin/ribonucleoside-diphosphate reductase subunit beta [Francisella tularensis subsp. holarctica LVS]	130	130	72%	3,00E-34	97%	Francisella sp. TX077308, complete genome	206	72%	1,00E-49	87%
>contig139909 length=167 numreads=7<br>ACCAACAACATTCCcTAAAGCATCTAGATGCGTTtCTAAaCcTGCTTCTTGCATCCA<br>ACT<br>ATCgATAACAGGAATAATTTGCTTGTGCTCTTTGCTACAAAACAGgcGAGTAACTC<br>CCCT<br>TTCTGATGAGCTAAAAGTCGCAGCTGTATCAAAGTACTTTTTTAACT	N-carbamoyl-L-amino acid hydrolase [Francisella sp. TX077308]	114	114	84%	7,00E-29	95%	Francisella sp. TX077308, complete genome	196	85%	5,00E-47	88%
>contig164444 length=145 numreads=7<br>ATTATTAGTCATAATCATTATGATCATTTAGATTCAAAAGTTATAAAaGATTTTCC<br>AAAT<br>AAAGAAGATATCAAAGTAATAGTACCTCTTGGGCTTAGTAGCTTTTTTATCAAAA<br>GAGGG<br>TTTAGTAATGTAACTGAAATGAACT	outer membrane protein romA [Francisella sp. TX077308]	92.4	92.4	85%	3,00E-21	92%	Francisella sp. TX077308, complete genome	193	86%	5,00E-46	90%
>contig182212 length=129 numreads=7<br>TTATTCAAGTCAACAGAAGATTTAGTTGGTGTTGTTGGAGAATTAAATGTATCTC<br>CTAAT<br>GCTGTTAATGTGATTCCTGATCATGTGGATATGACGATTGAAATACGTTCTCCAC<br>ATAGT<br>CACCTAAGA	N-carbamoyl-L-amino acid hydrolase [Francisella sp. TX077308]	88.6	88.6	82%	2,00E-19	98%	Francisella sp. TX077308, complete genome	187	75%	2,00E-44	95%
>contig92296 length=226 numreads=10<br>CAAAAGCTGGAGATTTAGATTATTATTTTATTGCTGGAGAAAAGATAGCAGAAG<br>TAACAG<br>AAACATTCAGCCGAATAACAGGTAAGACAATTCTTCCACCATATTGGAGTTTGTA<br>TTATT<br>CTGGTTCAACAATGACTTATACTGATTTACCTGATGCTCAGCATCAAATGTCAAA<br>TTTCA<br>TAAATGATTGTCAGCAGTATAATATCCAATGTGGATCATTTCAGCT	Alpha-glucosidase [Francisella cf. novicida 3523] >ref|WP_014548203.1| alpha-glucosidase [Francisella novicida] >gb|AEE26202.1| Alpha-glucosidase [Francisella cf. novicida 3523]	141	141	90%	4,00E-37	88%	Francisella sp. TX077308, complete genome	185	91%	1,00E-43	81%
>contig103984 length=207 numreads=10<br>TTTAATCGTTCTTCAAAGTCACCTCTAAaTTTTGCACCTGCTAGcaGTGCACCCATA<br>TCT<br>AAGGATAGTACTTTTTTTCCCTTTatCCcTtCAGGAACTTCATCATTGATTATCCTTT<br>GA<br>GCTAAGCCCTCTACTATAGCTGTTTTACCTACCCCTGGCTCACCGATTAGTACAG<br>GATTA<br>TTCTTAGTACGTCGCTGTAATACTTGT	protein disaggregation chaperone [Francisella philomiragia] >gb|EET20631.1| chaperone clpB [Francisella philomiragia subsp. philomiragia ATCC 25015]	137	137	86%	1,00E-35	97%	Francisella sp. TX077308, complete genome	178	87%	2,00E-41	82%
>contig90764 length=229 numreads=9<br>TAACAACACTTACGCCAGCCCTAGCTTTACCAAATATTGTATTTTCACcTTCAACA<br>AGTA<br>TCACAACTGAACCACTATACTGACCACCTATAGCGATACCTTGCAGTATTCTAAA<br>AGCAA<br>CTAAAATCAGTGGTGCAAATATACCAATGGTTGCATAAGATGGTAAAAGTGCCA<br>TACCTA<br>CGGTAGGAACCCCCATAAAGAATGCGGCTAAAGCTAGAGCAGTTGTTCT	major facilitator transporter [Francisella philomiragia] >gb|EET20522.1| metabolite:H+ symporter [Francisella philomiragia subsp. philomiragia ATCC 25015]	133	133	88%	3,00E-35	88%	Francisella sp. TX077308, complete genom	172	83%	1,00E-39	81%
**Contigs**	**Blastx**	**Max score**	**Score**	**Query Cover**	**e-value**	**Identity**	**Blastn**	**Score**	**Query Cover**	**e-value**	**Identity**
>contig152012 length=156 numreads=7<br>GCTATTTCTGTTGGTGTTTATAATGGCGAGCCAGTACTAGATCTTGACTATGATG<br>AAGAC<br>TCAAATGCTGAGACAGATATGAATGTAGTAATGAATTCTGAAGGTGGTATGATT<br>GAGATT<br>CAAGGTACAGCAGAAGGTAAAGACTTTTCAGAAGAA	ribonuclease PH [Francisella philomiragia] >gb|EET20213.1| ribonuclease PH [Francisella philomiragia subsp. philomiragia ATCC 25015]	102	102	88%	6,00E-25	94%	Francisella sp. TX077308, complete genome	167	85%	3,00E-38	87%
>contig198083 length=107 numreads=6<br>CTGTAACTGGTGTTGAAATGTTCCGTAAGCTTCTTGATCGTGGAGAAGCTGGtGA<br>TAACG<br>TTGGTGTaCTAGTTCGTGGTCTTAAGAGAGATGATGTTGAGCGTGGT	translation elongation factor Tu [Francisella sp. TX077308]	71.2	71.2	80%	2,00E-13	94%	Francisella sp. TX077308, complete genome	150	78%	2,00E-33	92%

>contig163781 length=146 numreads=6<br>AATTAATTAATCCTGACGTAACGCTAGCTTATGTAACCCCTAAAGAAGGAACAAA<br>TATTT<br>GGTTTGATATGCTTATGATTCCAAAAGGTGCCAAAGATGTTGATAGTGCCTATGA<br>GCTTA<br>TGAACTATATCATAGATCCTTATGTA	putrescine ABC transporter putrescine-binding protein PotF [Francisella sp. TX077308]	92.0	92.0	75%	1,00E-20	91%	Francisella sp. TX077308, complete genome	150	75%	4,00E-33	86%
>contig145874 length=161 numreads=6<br>ATAGTTAATCCTACAAAACCAATAAtcAAACAAAGTATAGATGGAAAAATAAACA<br>TTCCA<br>ACCACGTTGCCATTAAAGACAACTTGAGAACCAAAGATTGCTAcaccTGCAGCTA<br>AAGCC<br>CCACCAATATTATGAGACATATTCCACAAACCTAAGAAGAA	sugar phosphate antiporter [Francisella noatunensis subsp. orientalis str. Toba 04]	90.9	90.9	78%	8,00E-20	78%	Francisella sp. TX077308, complete genome	143	75%	7,00E-31	83%
>contig81334 length=248 numreads=8<br>GAGAAAAAGGGAACGCATAATACCCTAaTGGAAAAAATTCTTTCTCAAGGATAT<br>ATAAGA<br>GTACGCATAAATGGCGAGTTTTATAACCTTGATGAAGAGCACCCAGAATTAGAT<br>CGCTAT<br>AAGAAACATAATATAGATGTAGTTGTCGATCGCTTTAAACCTAAAAAAGATAAT<br>GACCAG<br>CGTATAGCTGAATCTATAGAAACGGCTTTAGACTTGGGTAATGGTATTATTAAAG<br>TTGCA<br>AATATGAA	excinuclease ABC subunit A [Francisella cf. novicida 3523] >ref|WP_014547926.1| excinuclease ABC subunit A [Francisella novicida] >gb|AEB28472.1| Excinuclease ABC subunit A [Francisella cf. novicida 3523]	155	155	89%	1,00E-41	87%	Francisella sp. TX077308, complete genome	141	65%	3,00E-30	81%
>contig130219 length=176 numreads=7<br>CTCTTTGCTTTGTGCAAATTTTATAAACCTAACTTTCTCATGCTCAAAATCAAATTC<br>ATC<br>ATCAGTAAAACCAACTTCTTTTTTTAGTTCAGCAGGTAAAAGTGAAAGAAGTAGA<br>TCTCT<br>TGCTAATTCCATAGCTCGTAAGCCAACATCTTTTTGCTTATATTCATAAACCATAT	Cyanophycin synthase [Francisella cf. novicida 3523] >ref|WP_014548065.1| cyanophycin synthetase [Francisella novicida] >gb|AEB28614.1| Cyanophycin synthase [Francisella cf. novicida 3523]	73.9	73.9	85%	2,00E-13	86%	Francisella sp. TX077308, complete genome	119	84%	1,00E-23	79%
>contig126294 length=180 numreads=10<br>CTATGATAAGTGCAaTGGCAGTTTTAAATGGCTTTGTTGGTATAAATacATTCATA<br>AGCT<br>TTGGGCCTACTATATTTGATGAGTTATCTGGATGCActCAGTGTGGTGCTAGCTAT<br>TATG<br>GGACTGCAATGACATTAGTCAACCTTATTGCTACTGTTATAGGATTACTTTTAGTT<br>GATG	Arabinose-proton symporter [Francisella cf. novicida 3523] >ref|WP_014548791.1| MFS transporter [Francisella novicida] >gb|AEE26810.1| Arabinose-proton symporter [Francisella cf. novicida 3523]	94.4	94.4	85%	3,00E-21	76%	Francisella sp. TX077308, complete genome	108	74%	2,00E-20	79%
>contig165644 length=144 numreads=6<br>AAAAGTACCTTCACGCCTTTGGGTAGTAAGAGCCTCATCTATATCTGCAATAAAA<br>TTATA<br>GATATTCCAAGGAGTAAAATAGCATCCAGCTCGTGCAAATCCTAAAACAAAAAC<br>TATCAA<br>CACAAATGCAACAAAAGCAACTAC	Rhamnogalacturonide transporter RhiT [Francisella cf. novicida 3523] >ref|WP_014548204.1| Rhamnogalacturonide transporter RhiT [Francisella novicida] >gb|AEE26203.1| Rhamnogalacturonide transporter RhiT [Francisella cf. novicida 3523]	70.1	70.1	52%	2,00E-12	97%	Francisella sp. TX077308, complete genome	106	62%	7,00E-20	84%
>contig55685 length=334 numreads=15<br>AAACCTGAGCCTCGTATTGCTTGAAGTAAGAAGGTTTCAGCTTGGTCATCTTGGT<br>GATGT<br>CCTAACAGTAAAATAGGAGCAGGGTAGTTAGCCATTTGTTGTTCAAAAAAAGCT<br>ATTCGT<br>TGCTTGCTTGCCCAAGCTTCAAAACTTTCTCCTTTAGGTACTTTTCCAAGTTTATGT<br>GTA<br>ATAAATGCGATATTATGTTCATCACAAATATTGTTACAATACTGTTGCCAACTATT<br>AGCA<br>TCAGAGTGAATAGCATGGTTTATATATATAGCTATgATtGGAATTTTAATTTCTTT<br>GAAA<br>ATATCAACTAATACGCTAGAGTCAATGCCACCAC	tRNA(Ile)-lysidine synthetase [Francisella cf. novicida 3523] >ref|WP_014548610.1| tRNA(Ile)-lysidine synthetase [Francisella novicida] >gb|AEE26621.1| tRNA(Ile)-lysidine synthetase [Francisella cf. novicida 3523]	134	134	91%	5,00E-35	68%	Francisella sp. TX077308, complete genome	93.5	41%	1,00E-15	78%
>contig149397 length=158 numreads=10<br>CAAAGATTCTTCTTAAATCACCGTCAGTAAATATACCTAGTAGCTCATTGTTTTCA<br>GTAA<br>TAAGAGTATTACCTATACCTTTACTACTAATTTCGAGTATTGCCTTACGAATAGTG<br>TCTG<br>TTGGGTTTATAGTGGGTATTTCATTTTCTCTACGCATA	arabinose phosphate isomerase [Francisella tularensis subsp. tularensis SCHU S4]	87.8	87.8	81%	2,00E-19	77%	Francisella sp. TX077308, complete genome	62.1	20%	2,00E-06	95%
>contig74482 length=266 numreads=14<br>cccctcgttcgctCCTAaCTCTATGTCGAGTAGGTAACCAGACTGCTCTGGTTGGGTTC<br>C<br>CCCATTCGGAAATCTCCGGATCATAGGTCATTTACCACCTAACCgAAGCTTATCGC<br>AGAT<br>TAACACGTCCTTCATCGCCTCTGAATGCCAAGGCATCCGCCACTTGCACTTATTTT<br>CTTA<br>AGTCTATCTCTATACTAAATTGTTAAATATCTCTATCTTCATGCAAATAAATATTG<br>GTGG<br>AGCCTAGCGGGATCGAACCGCTGACC							Francisella tularensis subsp. holarctica PHIT-FT049, complete genome ribosomal rRNA	1111	90%	4,00E-99	92%

>contig75395 length=263 numreads=9<br>GTCTtCAGTCAACATCTAGAGGTAGAATTACTGCTCGTCAAATCGAAGCAGGAA<br>GAAGAG<br>CTATTAACCGTCATGTTAAGCGTGGTGGTAAGGTTTGGATCAGAATTTTTCCAGA<br>CAAGC<br>CTATTACTAAGAAACCTCTTGAaGTTCGTATGGGTAAAGGTAaaGGTtCGGTTGA<br>GTATT<br>GGGTTGCTCAAATTCAACCAGGTCGTGTACTATATGAAATAACTGGTGTTAAAG<br>AAGAGC<br>TAGCACGTGAAGCTTTCCAGAGA	50S ribosomal protein L16 [Halomonas] >gb|EGP19020.1| 50S ribosomal protein L16 [Halomonas sp. TD01] >gb|EHA14395.1| 50S ribosomal protein L16 [Halomonas sp. HAL1] >gb|EHJ94064.1| 50S ribosomal protein L16 [Halomonas boliviensis LC1] >gb|EHK60828.1| 50S ribosomal protein L16 [Halomonas sp. GFAJ-1] >emb|CDG52359.1| 50S ribosomal protein L16 [Halomonas sp. A3H3]	159	159	85%	1,00E-47	88%	Francisella tularensis subsp. holarctica F92, complete genome	313	85%	8,00E-82	89%
>contig46885 length=388 numreads=16<br>TTCTACTATAGACAATTACATAGCAATTCTAAAATGATAAAATAACtATAAGATTT<br>CACT<br>AAACTTTAACTAAAATGATCAAAGTTACTTCATTTAACGCAAATGGTATCCGTGC<br>AGCTA<br>CTCGTAaGGGTTTTTGGGATTGGTTCAAAACTCAAGATATCGACTTTTTATGTATC<br>CAAG<br>AAACAAAGGCTCAATTTCATCAACTTGAGGGAGATAGTCAGTACTTCCCCGAAG<br>GTTATC<br>ATTATGACTTTAAAGATGCTGTTAAAAAAGGCTATAGTGGTACTGCTATTTATGC<br>AAAGA<br>AAAAACCTCTAAAAGTTATCAAAGAGCTTGGGTTTGACTGGGCTGATAATGAAG<br>GTCGTT<br>ATATTCAATTTGATTATGAGAAttttAG	hypothetical protein [Francisella cf. novicida Fx1] >ref|WP_014549715.1| hypothetical protein [Francisella novicida] >gb|AEB27795.1| Exodeoxyribonuclease III [Francisella cf. novicida Fx1]	196	196	76%	6,00E-60	88%	Francisella tularensis subsp. holarctica PHIT-FT049, complete genome	292	72%	1,00E-75	85%
>contig100814 length=212 numreads=10<br>TATAACTTTTCTATAaGGTGCTTCTAAGAAACCATAATCATTTACACGAGCATAAC<br>TTGC<br>CAATGAGTTAATTAGACCAATATTTGGACCCTCTGGAGTTTCAATCGGACATAAT<br>CTACC<br>ATAGTGAGTAGCATGTACATCACGCACTTCAAAGCCTGCTctATCTCTAGATAAAC<br>CACC<br>TGGGCCTAAAGCTGAGATTCTACGCTTATGCG	DNA-directed RNA polymerase subunit beta [Francisella tularensis subsp. holarctica F92] >ref|WP_015083882.1| DNA-directed RNA polymerase subunit beta [Francisella tularensis] >gb|AFX71360.1| DNA-directed RNA polymerase subunit beta [Francisella tularensis subsp. holarctica F92]	156	156	80%	5,00E-42	99%	Francisella tularensis subsp. holarctica PHIT-FT049, complete genome	278	79%	2,00E-71	91%
>contig89845 length=230 numreads=9<br>ATCttCTCTACCAAATGCAAAACCAATTGGCTCCATACCCATAGACTCTAAAGATA<br>CCGT<br>ACCTGCTAGCACAATCAAATCAGACCATGAAACAGCATCACCGTATTTTTGTTTA<br>ATTGG<br>CCATAAAAGTTGACGGGCTTTATCAAGGTTTAcATTATCTGGCCAGCTGTTTAAT<br>GGAGA<br>GAACCTTTGTTGGCCACGGTTAGCTCCTCCACGACCATCAGATACTCTAT	catalase/peroxidase HPI, partial [Francisella tularensis]	154	154	91%	7,00E-45	96%	Francisella tularensis subsp. holarctica F92, complete genome	248	91%	2,00E-62	86%
>contig137266 length=169 numreads=6<br>TACAAAACCTGCTGTATTACCTAATGGTAAATCAGTACCAGAAGTTTTGTtATCA<br>GGCAA<br>CCATAAAAATATAGCAAAATGGAGGCGTAAACAAAAGTTAATAAGAACTTATGA<br>GCGTCG<br>TAAAGATTTAATGAAGTGCCTATGCCTATCACCAGAAGATAAGCAATTA	hypothetical protein [Francisella cf. novicida Fx1]	107	107	83%	6,00E-27	84%	Francisella tularensis subsp. holarctica PHIT-FT049, complete genome	215	82%	1,00E-52	90%
>contig116524 length=190 numreads=6<br>AATGTAAATaaccTAGAGCAGGTTAGAGCTGTGATGGAAGCTGCTGATAAAGtAA<br>acTCA<br>CCAGTTATCCTACAAGGTTCTGCAGGTGCAAGAAAGTACGCAGGTGCTCCTTTCA<br>TCAGA<br>CATCTTGTATTAGCTGCTATTGAAGAGTATCCACATCTGCCTGTATGTATGCACCA<br>AGAT<br>CATGGTACAT	fructose-1,6-bisphosphate aldolase [Francisella noatunensis subsp. orientalis str. Toba 04]	133	133	80%	1,00E-35	98%	Francisella tularensis subsp. holarctica PHIT-FT049, complete genome	191	80%	3,00E-45	85%
>contig83550 length=243 numreads=10<br>ATCAATGATAATAAGGTTACAATCTCTTCAAATAATAGTAAATTTAACTTGATGTC<br>ACTG<br>AATGCTGACAATTTTCCTCTAGCAGATGGTAACATTCAAGAAGAGTCAGGTTTTG<br>ATCTA<br>TCTCAACAAGATTTTCATCATATTATTTCAAAAATTGATTTTTCAATGGCAAATGA<br>TGAT<br>ACAAGATATTTCCTTAACGGAATGTTTTGGGAAGTGAATGGTAATCTTTTAAGAG<br>CGGTA<br>TCT	DNA polymerase III subunit beta [Francisella tularensis] >gb|EET20098.1| DNA polymerase III [Francisella tularensis subsp. tularensis MA00-2987]	145	145	89%	5,00E-40	83%	Francisella tularensis subsp. holarctica PHIT-FT049, complete genome	183	87%	6,00E-43	80%
>contig153145 length=155 numreads=8<br>TTTCTtGAGTAGAAACATCAATAAGCTTATGATCAAAGGCTTTTAATCTTATTCTG<br>ATTC<br>TTTGATTATTTATAGCCATTTTATTTTTATCcTTCTAATTATGAGTTACGAGATTTG<br>ATG<br>ATATCTTCAGCGATATTGTTTGGAACTTCAGCATAAaCcGGCGCCGATGCAATCG<br>TACTCATGACCGAATGGAATCAGTACC	30S ribosomal protein S10 [Francisella tularensis subsp. holarctica LVS]	55.1	55.1	32%	6,00E-08	96%	Francisella tularensis subsp. holarctica PHIT-FT049, complete genome	187	65%	4,00E-44	89%

<table>
<tr><th></th><th></th><th></th><th></th><th></th><th></th><th></th><th></th><th></th><th></th><th></th><th></th></tr>
<tr><td>>contig67256 length=287   numreads=13
CAGATGGAAaaGGCATTTTGTATTCTGCATTTTTTTCTTCTTTATAGATAACctCAA
TAA
ACTCATCACCTAAAACCACTTCAGCACCTAATGCTTCAAGCCCCTCAAGATGTAT
ATCAA
AAGGTCTTTTTTCACTAAAACTACAGCCTCCTGGAAAACCAATTTTTACACGACTT
TTTt
GCTTTAGCATCGCTCCTAGAAGCATTAGTGAACAACGAGTTTTTGATGTTTGATT
ACCAC
ATAACTCTAACGTATCCTTTGTAATTTTACTTGTATCTATAGCTACA</td><td>UDP-N-acetylglucosamine 1-carboxyvinyltransferase [Francisella tularensis] >gb|EBA52105.1| UDP-N-acetylglucosamine 1-carboxyvinyltransferase [Francisella tularensis subsp. holarctica 257]</td><td>166</td><td>166</td><td>86%</td><td>2,00E-48</td><td>81%</td><td>Francisella tularensis subsp. holarctica F92, complete genome</td><td>182</td><td>68%</td><td>2,00E-42</td><td>81%</td></tr>
<tr><td>>contig116293 length=191   numreads=6
AATCTCTGTTAGAGATTTCTTACCTAAATTAGGTATTTTCATCAACTGTGACTCAG
AATA
CTGTACCAAATCACCAAGATACTTGATATtttCAGCTCTTAAACAGTTAGAAGATC
TAAC
AGTAAGCTCTAAATCATCAATTGGTTTTAGTAACATTGGGTCAATATTAGAATCC
GCCAA
AGGATCCCCCG</td><td>DNA-directed RNA polymerase subunit alpha [Francisella noatunensis]</td><td>122</td><td>122</td><td>82%</td><td>9,00E-32</td><td>94%</td><td>Francisella tularensis subsp. holarctica PHIT-FT049, complete genome</td><td>176</td><td>71%</td><td>8,00E-41</td><td>86%</td></tr>
<tr><td>>contig81323 length=247   numreads=9
TATAGATGCAGAAGCTAGTGATATAACTATCGAGATACAAGAGGGCGGTAAGT
CATTTAT
TCGTATAAGAGATAATGGTAAAGGTATTTCAAAAGATGATTTAGCACTAGCACT
AGCACC
TCATGCTaCAAGTAAGGTTTATAATCTTGATGAGCTAgAATCTGTAGCTAGTATG
GGTTT
TCGTGGCGAGGCTTTAGCAAGTATTGGTTCTGTTGCAAAAGTTAAGATAATCTCA
AAACA
cATAAGT</td><td>DNA mismatch repair protein [Francisella tularensis subsp. tularensis SCHU S4]</td><td>135</td><td>135</td><td>82%</td><td>4,00E-35</td><td>84%</td><td>Francisella tularensis subsp. holarctica F92, complete genome</td><td>182</td><td>71%</td><td>2,00E-42</td><td>82%</td></tr>
<tr><td>>contig170585 length=140   numreads=7
AAGATACGAATCTATAGCAAATGCTGATTTAAaCGAAGGTGATGCTGATTATGC
AATCAA
AGTTGATTTTGATAAAGATGCAAAAACTATAACTGTTAGCGATAATGGtaTCGGT
ATGAC
TGAAGAAGAGGTTATCGAAA</td><td>chaperone protein HtpG [Francisella sp. TX077308] >ref|WP_013922041.1| heat shock protein 90 [Francisella sp. TX077308] >gb|AEI35188.1| Chaperone protein HtpG [Francisella sp. TX077308]</td><td>83.6</td><td>83.6</td><td>77%</td><td>4,00E-17</td><td>87%</td><td>Francisella tularensis subsp. holarctica PHIT-FT049, complete genome</td><td>167</td><td>69%</td><td>3,00E-38</td><td>91%</td></tr>
<tr><td>>contig140423 length=166   numreads=6
TAAGTTTAaGTTCACTTAACCATTGAACAAAAATAGCGGCCATATTTGGATTAAC
AGATG
ATCTTtttGCATATAAATCACCAGAATCTGTGGTTATCTCAGTCATAGAAAGACCA
ATAA
GCCCAGTACAAGAAACATCCCCTATATCCTTACAAAGATATCCTTT</td><td>hypothetical protein [Francisella philomiragia] >gb|EET21254.1| conserved hypothetical protein [Francisella philomiragia subsp. philomiragia ATCC 25015]</td><td>110</td><td>110</td><td>87%</td><td>2,00E-27</td><td>93%</td><td>Francisella tularensis subsp. holarctica PHIT-FT049, complete genome</td><td>163</td><td>88%</td><td>5,00E-37</td><td>84%</td></tr>
<tr><td>>contig185854 length=125   numreads=6
CGACAACAACCCATACAAATGTAAGTGGTATAAATATTTTCCAACCTAAACGCAT
AATTT
GGTCATATCTGTATCTTGGGTAAGTAGCTCTTATCCATAAAAACATAAACATAAA
TATAC
CTGTT</td><td>NADH dehydrogenase I subunit H [Francisella tularensis subsp. mediasiatica FSC147]</td><td>85.9</td><td>85.9</td><td>71%</td><td>8,00E-19</td><td>95%</td><td>Francisella tularensis subsp. holarctica PHIT-FT049, complete genome</td><td>150</td><td>68%</td><td>3,00E-33</td><td>90%</td></tr>
<tr><td>>contig152091 length=156   numreads=7
TTtCAAAATTGTTGGTTTTATATAGTTACCATCATTTGCAATAGCAGATGCTCCTG
CAAC
TAGTTGCATATCAGTAGCATTCATACCATAACCAAAAGATAATGTAGCAAGTTGG
AAATC
ACCCCACTTATCTTTAACAGGCACAAATCCTTCTCG</td><td>Cell division protein FtsI/penicillin-binding protein [Francisella noatunensis subsp. orientalis str. Toba 04] >ref|WP_014715143.1| cell division protein [Francisella noatunensis] >gb|AFJ43612.1| Cell division protein FtsI/penicillin-binding protein [Francisella noatunensis subsp. orientalis str. Toba 04]</td><td>104</td><td>104</td><td>75%</td><td>1,00E-24</td><td>92%</td><td>Francisella tularensis subsp. holarctica PHIT-FT049, complete genome</td><td>139</td><td>61%</td><td>9,00E-30</td><td>87%</td></tr>
<tr><td>>contig85599 length=239   numreads=12
TATTCCACCCTCTtttAGGAAGCTCTGTAATACTTGTTCATATGCTCCAATATGTTT
GAT
TAGATATTCACAAAATTCTCTAACAGCACCTTCGCCACCATTATTTTGACATCTTA
CATC
AGCATAATCCTTAACAATATCCATAGAATTCGCTGGAGCTGCAGATATCCCAACT
TTATT
CATAAGAGTTATATCAGGCAAATCATCACCCATATAAGCAATTTGTGAATCGTTG
AGAC</td><td>3-deoxy-D-manno-octulosonate 8-phosphate phosphatase [Francisella cf. novicida 3523]</td><td>115</td><td>115</td><td>85%</td><td>4,00E-30</td><td>65%</td><td>Francisella tularensis subsp. holarctica PHIT-FT049, complete genome</td><td>143</td><td>60%</td><td>1,00E-30</td><td>82%</td></tr>
<tr><td>>contig110799 length=198   numreads=7
TTCTCTTTTCAAGATGATTCGTAACACTACAAATTGCTCACCTGAAGGTGTACTTT
CTGC
ATACAAAGATAATGCcTCTGTGATAGAGGGTGCAACGGCTCAAAGGTTTTATAG
CAACCC
TGAAAACAATGTTTATAACTTCCATCAAGAAGAAGTTGATATCTTAATGAAGGTT
GAGAC
TCACAATCACCCTACAGC</td><td>phosphoribosylformylglycinamidine synthase [Francisella tularensis subsp. holarctica]</td><td>119</td><td>119</td><td>78%</td><td>8,00E-29</td><td>85%</td><td>Francisella tularensis subsp. holarctica PHIT-FT049, complete genome</td><td>130</td><td>59%</td><td>6,00E-27</td><td>83%</td></tr>
</table>

>contig134387 length=172 numreads=8<br>AAAATACTGTAAACCAGTATGAAGTTGATAATTCAAATGCCCATGCATCACCTGC<br>AGTTA<br>GAAAGCTTGCAAGAATCTTGAATGTAAACCTAAGCAAGGTAAAAGCGACTGGTC<br>GCAAAG<br>GTAGAGTTTCAAAAGAAGATTGTTATAACTATATTAAAAATGCTGTAACACA	dihydrolipoamide acetyltransferase component of pyruvate dehydrogenase complex [Francisella sp. TX077308] >ref|WP_013922551.1| dihydrolipoamide acetyltransferase [Francisella sp. TX077308] >gb|AEI35711.1| Dihydrolipoamide acetyltransferase component of pyruvate dehydrogenase complex [Francisella sp. TX077308]	100	100	75%	1,00E-23	88%	Francisella tularensis subsp. holarctica PHIT-FT049, complete genome	126	73%	7,00E-26	80%
>contig146319 length=160 numreads=9<br>CCTATTTCACTTCTtGGTTGTATAGCTATACTTTTGCTGATGCAACCAGATTTTGGT<br>TCT<br>ACAGTtgTTATATCAATATGTGTTCTGGGCATGCTTTTTGTTGGTGGTAATAAAGT<br>TCAA<br>TGGTATGGTTTACTTATTGTTGCTATGATAccAATGGCTG	cell division protein FtsW [Francisella sp. TX077308] >ref|WP_013923546.1| cell division protein FtsW [Francisella sp. TX077308] >sp|F8GAB4.1|FTSW_FRAST RecName: Full=Lipid II flippase FtsW; AltName: Full=Cell division protein FtsW >gb|AEI36717.1| Cell division protein FtsW [Francisella sp. TX077308]	92.0	92.0	85%	1,00E-20	85%	Francisella tularensis subsp. holarctica PHIT-FT049, complete genome	122	64%	8,00E-25	85%
>contig108310 length=201 numreads=8<br>GCAAAAGTATAAAAGTATCGCTTTGCATATTGACTGTTCAACTAGGAAGCAAAA<br>TATCGA<br>TCAAAAATATATTGATGCTTTAAATAaGTGTGCAAATGAAGTTGTAACTCCTGAA<br>ATGAT<br>ATATTGTTGTGGTTTTGCTGGTGATAAAGGCTTTAGTGTACCGGAGTtAAATGCT<br>AGTAG<br>TTTATCGGGATTGCAGTCTCA	4Fe-4S ferredoxin [Francisella tularensis] >gb|EBA52400.1| 4Fe-4S ferredoxin, FAD dependent [Francisella tularensis subsp. holarctica 257]	106	106	89%	2,00E-26	74%	Francisella tularensis subsp. holarctica PHIT-FT049, complete genome	89.8	76%	1,00E-14	77%
>contig59621 length=316 numreads=10<br>AATTATGAATATTGTGTATGGGTATCTTACGCTGCTGATATTTATGATCTTTAAAA<br>TAAT<br>ATACTATCCACCCTTAGCTAAAATAGCAGGGCGTTTAAGCAAGCTGGTAAATTTT<br>TAATC<br>AAAACTACCACATCAAAACCATAGTACTAAACTATGgTTTTTCTTAATGATAGCCA<br>TCTA<br>CTACTATCACATAaGCACACCATAAAATACTAAATTTTAGGTGAGTttATTAGATG<br>ATTT<br>ATTAcTTTtGTCAAAGCAAGTAAATAATCAAGCTAGTAGCACTAGCAAATATAAA<br>CCCCT<br>TTAGGCTACTTTATAA							Francisella tularensis subsp. holarctica PHIT-FT049, complete genome 3-dehydroquinate synthetase	75.0	27%	4,00E-10	83%
>contig134628 length=172 numreads=6<br>GACTTATGGTCTTTTTGGAAGTTTTTGAATAGCTTAGCTGATGCGCCTGGCGaTT<br>CCATa<br>gAAAGAGCAAGAacAAGAGGACCGACTAAAGCGtCATCAAGACACTCAAAGTCA<br>GTTCCT<br>TtAaTTGCTAAACGTGCTAAGTTGTTGCGTACAACTCTTAAATAAACTCCAG	50S ribosomal protein L10 [Francisella tularensis subsp. mediasiatica FSC147] >ref|WP_012429128.1| 50S ribosomal protein L10 [Francisella tularensis] >sp|B2SFD4.1|RL10_FRATM RecName: Full=50S ribosomal protein L10 >gb|ACD30287.1| 50S ribosomal protein L10 [Francisella tularensis subsp. mediasiatica FSC147]	103	103	82%	1,00E-25	84%	Francisella tularensis subsp. mediasiatica FSC147, complete genome	224	81%	2,00E-55	91%
>contig96801 length=218 numreads=12<br>AAAGGCGCAAAATATTTGTAATATACTCTTTTTCAACCTTTGTTATTTTGCCATTTT<br>TCC<br>AGTcaGTAACATCTTCAGAAAGATCAATTTCATCTTCTATCCAGTGAGCTTTTTCA<br>TGCT<br>TAACCGTTAGGTCAACAGCCCATGGATAACAAAAAGGTTTATAAGTTTTTGAAG<br>CAGTAG<br>TTAATGATGAGCCTTTAACTCGATTAATAACCTCACCA	bifunctional gluaredoxin/ribonucleoside-diphosphate reductase subunit [Francisella noatunensis subsp. orientalis str. Toba 04]	142	142	75%	2,00E-38	96%	Francisella tularensis subsp. mediasiatica FSC147, complete genome	222	76%	1,00E-54	85%
>contig87468 length=235 numreads=10<br>TGGCTTTATTTTTACTTGGCACATATATTATTTTGAAATAATAATCTGGCACAGCA<br>ACCT<br>TGTTATAACCGATAGTTTTATATACTTTTTGCTTTTTATATATTGGACCTGTATACA<br>CAT<br>AAATACTATCATACATATTTGCCCAGATTCTCTCATCTGTTTCTAATCTTTCCCAAC<br>CTT<br>GCCTATTTAAGCCCGCCTTTTGTGGCGACATATTTGATAGTAAAAATGATTCATC	DNA/RNA endonuclease G [Francisella philomiragia subsp. philomiragia ATCC 25017] >ref|WP_012279592.1| DNA/RNA endonuclease G [Francisella philomiragia] >gb|ABZ86251.1| DNA/RNA endonuclease G [Francisella philomiragia subsp. philomiragia ATCC 25017]	158	158	85%	7,00E-45	94%	Francisella tularensis subsp. holarctica PHIT-FT049, complete genome	226	84%	9,00E-56	84%
>contig106923 length=201 numreads=8<br>ATGCTCAGCATTAGCTATAGCTGAATTAAGAACTTCCTTAATTAGCACAGCAGCT<br>TTTTT<br>GTTACTGAATGTCAAAAGATTGaTAGCTTTCTCTACAGGTAGTCCTCTGATTTGAT<br>CAGC<br>AACTAACCTACACTTTTGAGGTGAGATtCttgCAAAttttAATTTAGCTTGTACTTCC<br>AT<br>AATTACCTCAACTATTTCTTC	50S ribosomal protein L22 [Francisella philomiragia] >gb|EET20366.1| 50S ribosomal protein L22 [Francisella philomiragia subsp. philomiragia ATCC 25015]	116	116	82%	2,00E-31	93%	Francisella tularensis subsp. mediasiatica FSC147, complete genome	207	88%	3,00E-50	86%

<table>
<tr><td>>contig97529 length=217   numreads=10
TAGTCAATTTAGGAAACAAAACGGTTTATTTATATCTATAAACCATAAAAGATAT
ATTGG
TCAAATTCTCGAAAGATACGATTATAACAGCATAGACCTTATTCTTGTAACAGAT
GGTGA
AGCTGTACTTGGgaTAGGAGATCAAGGTATAGGTGGAATGAATATCAGTATCGG
TAAGAT
TATGGTATATGTTGCTGCAAGTGGTATTGACCCAGCT</td><td>malate dehydrogenase [Francisella tularensis subsp. mediasiatica FSC147] >ref|WP_012429279.1| malate dehydrogenase [Francisella tularensis] >gb|ACD30522.1| NAD-dependent malic enzyme [Francisella tularensis subsp. mediasiatica FSC147]</td><td>135</td><td>135</td><td>84%</td><td>2,00E-35</td><td>89%</td><td>Francisella tularensis subsp. mediasiatica FSC147, complete genome</td><td>185</td><td>82%</td><td>1,00E-43</td><td>83%</td></tr>
<tr><td>>contig62529 length=303   numreads=14
tGCTTTGTATGAGCATAAAaTATTTGTTCAAGGTGTTATCTGGGAAGTCTATTCCT
TCGA
TCAGTGGGGCGTGCAGTTAGGCAAAGTCTTAGCGACGCAGTTGCTCTCTGATGT
GCAGTC
CACGCTGCCAGTCACTCAATACGACAGTTCAACCAATGGCCTACTCAACCATTTT
AAGGC
TATTAATAAACCCTAGTTTATTATTACTGTAGCTTGTGTCAGCGTACCCCGCGAA
GCGTT
GTGGGTATTTGCCCGACAAAATTTATCATCATGTAATGAATAATAATGCGGCTGT
AATCT
TCA</td><td>hypothetical protein EMIHUDRAFT_446110 [Emiliania huxleyi CCMP1516] >gb|EOD11446.1| cytosolic glucose-6-phosphate isomerase, partial CCMP1516]</td><td>97.1</td><td>97.1</td><td>57%</td><td>3,00E-21</td><td>68%</td><td>Francisella tularensis subsp. mediasiatica FSC147, complete genome</td><td>75.0</td><td>25%</td><td>4,00E-10</td><td>82%</td></tr>
<tr><td>>contig95667 length=220   numreads=11
ATATATCCTAGCTCAATTAGACGAGCCTATAGATGAGAAAAAAGAAGCTTATAA
ATTCTG
CAAAGATAATAaTTTTGATCcAGTTATTTCATCTATCACTATCGAGCGACTTAATC
GAGC
AGGtgAGAAAATTAAAGCTGATATACAAGCTGAGTTTGACACTGAAAACTCtAAA
AAGAA
ACCAAATCAAGAAAAGTtGACAGAGCTACAAGAGAAGTTA</td><td>transposase [Francisella tularensis subsp. tularensis NE061598]</td><td>129</td><td>129</td><td>85%</td><td>4,00E-36</td><td>89%</td><td>Francisella tularensis subsp. novicida U112, complete genome</td><td>307</td><td>86%</td><td>3,00E-80</td><td>92%</td></tr>
<tr><td>>contig137414 length=169   numreads=9
GTATGGAACTGTCCATCATTTAGTGAGCTCAATCTATCTGCTAATTTTTTACCAGC
TTTT
TGGTCAGCAAGAAGCTTATCCCAATCACTACTCCATAACACCTTGATAACATTCC
AACCA
GCACCTTCAAACACGCCTGCTAATTCTTCAACAATATTACCATTACCGT</td><td>Pyruvate dehydrogenase E1 component [Francisella cf. novicida 3523] >ref|WP_014548839.1| pyruvate dehydrogenase [Francisella novicida] >gb|AEE26858.1| Pyruvate dehydrogenase E1 component [Francisella cf. novicida 3523]</td><td>107</td><td>107</td><td>83%</td><td>4,00E-25</td><td>89%</td><td>Francisella tularensis subsp. novicida U112, complete genome</td><td>159</td><td>83%</td><td>7,00E-36</td><td>84%</td></tr>
<tr><td>>contig141718 length=165   numreads=9
AGTATTTACAAGCATtACACCCATTGGGTTACCAATGTTTTTACTTGTTACTTTTCT
AAA
GTTAGTAGCTGCACTTCTACTTAGCTCTACCGCTACTATAGGAGTACCTGTTTGAT
GATC
AACTGATGGACTAGCTCCAATAATATCAGCTCCACCAGCAACAGC</td><td>Protein-export membrane protein SecD [Francisella sp. TX077308] >ref|WP_013923354.1| preprotein translocase subunit SecD [Francisella sp. TX077308] >gb|AEI36523.1| Protein-export membrane protein SecD [Francisella sp. TX077308]</td><td>100</td><td>100</td><td>85%</td><td>5,00E-23</td><td>85%</td><td>Francisella tularensis subsp. novicida U112, complete genome</td><td>147</td><td>87%</td><td>5,00E-32</td><td>83%</td></tr>
<tr><td>>contig95191 length=221   numreads=10
CCATTCTCCTCTATAGATATTTGCTCTAAAACACTCTTAACCCACCTCCATCTAGTA
GAG
CCATCATGACTATAAGGtaTTTCCAACTCATGAAAGAATTTTGTTAAATTGGAACT
TGAG
CGATATATAAAATAGTCATATGGTTCATCTCCACAAATCATTTTTGCAAAATTATC
TAAA
AAATAGTTTCTGGATATTTCTAATGCTTGATCTATAAATTT</td><td>hypothetical protein FTN_1490 [Francisella novicida U112] >ref|WP_003040476.1| hypothetical protein [Francisella novicida] >gb|ABK90357.1| protein of unknown function [Francisella novicida U112] >gb|EDX20130.1| hypothetical protein FTE_0876 [Francisella tularensis subsp. novicida FTE]</td><td>141</td><td>141</td><td>89%</td><td>2,00E-39</td><td>88%</td><td>Francisella tularensis subsp. novicida U112, complete genome</td><td>289</td><td>89%</td><td>1,00E-74</td><td>90%</td></tr>
<tr><td>>contig80341 length=250   numreads=11
GAAACAGGCGAGACTGTATTCTTAAAAGCTAAGATAACAATTTTAGCTACAGGT
GGaGCT
GGTCGTATATATGAGTCAAGCACAAATGCTtATATcAaTACAGGtgATGGTATGG
GTCTA
GCCTTAaGAGCTGACATCCCATTGCAGGATATGGAATTTTGGCAGTTCCATCCAA
CAGGT
ATTGCTGGAGCTGGTGTACTTGTTACTGAAGGGTGCCGTGGTGAAGGTGGTATC
TTGCGT
AATAAAGATG</td><td>succinate dehydrogenase flavoprotein subunit [Francisella sp. TX077308] >ref|WP_013922344.1| succinate dehydrogenase flavoprotein subunit [Francisella sp. TX077308] >gb|AEI35501.1| Succinate dehydrogenase flavoprotein subunit [Francisella sp. TX077308]</td><td>171</td><td>171</td><td>87%</td><td>3,00E-48</td><td>98%</td><td>Francisella tularensis subsp. novicida U112, complete genome</td><td>257</td><td>87%</td><td>3,00E-65</td><td>85%</td></tr>
<tr><td>>contig109098 length=200   numreads=7
GCAGATGTATCAGAAATGTTACAGCCAGGTAGTATTCTttATACAGTTGTATTTGC
TGCT
ACGATTATATTCTTTTGTTTCTTCTACACTTCTTTAGTATTtAATCCTAAAGAAACG
GCG
GATAATCTTAAGAAATCTGGTGCTTATATTTCTGGTGTAAgaCCTGGCGAGCAAA
CTGCC
AAATATATTGATGCAGTAAT</td><td>preprotein translocase subunit SecY [Francisella cf. novicida 3523] >ref|WP_014547570.1| preprotein translocase subunit SecY [Francisella novicida] >gb|AEB28102.1| Preprotein translocase secY subunit [Francisella cf. novicida 3523]</td><td>137</td><td>137</td><td>84%</td><td>9,00E-37</td><td>97%</td><td>Francisella tularensis subsp. novicida U112, complete genome</td><td>193</td><td>84%</td><td>8,00E-46</td><td>84%</td></tr>
</table>

>contig87058 length=236 numreads=10<br>CAACTTCATTAACAGTCTTTCCTTTAGCTAAAGGTATACGATTTGCAAGCCTAAAA<br>ATAT<br>GTGTGTCAACAGCCATAACAGGTTTACCAAAAGCAGTATTTAAAACTACATTTGC<br>TGTTT<br>TACGACCAACACCGGCTAGAgATATTAGACTATCAAAATCATCAGGAACAGTACT<br>ATCAA<br>ACTTTTCTATCAAGTCTTTGCTAGTTTTGATTACATTTTTGGCTTTTGTCTTATAT	Endonuclease III [Francisella cf. novicida 3523] >ref|WP_014548454.1| endonuclease III [Francisella novicida] >gb|AEE26459.1| Endonuclease III [Francisella cf. novicida 3523]	146	146	91%	7,00E-42	91%	Francisella tularensis subsp. novicida U112, complete genome	187	92%	4,00E-44	81%
>contig87142 length=236 numreads=10<br>TTGAGCCTTTTTAAGAGCAACTGCAAGCACTTCTTTAGTGTTTATTACAaTAGTAT<br>CACT<br>TTCGCGTAAAAATCCCATTTCTTGTGCTTCATAACCACTTTTTGCAACTTGTGCGA<br>GGGC<br>TAAGTTTTTGTAGCGTTTTTCAAAGTCTTTCCATGAGTCTTGAGCATTAGATGCTC<br>GTTG<br>AGCCATTTCTTTAGAGCCACCCCAACCAGGGATAATTCCAACACCTGCTTCTACT<br>A	3-hydroxyacyl-CoA dehydrogenase [Francisella novicida] >gb|EDZ90840.1| fusion of 3-hydroxyacyl-CoA dehydrogenase and enoyl-CoA hydratase [Francisella novicida FTG]	135	135	87%	9,00E-35	82%	Francisella tularensis subsp. novicida U112, complete genome	150	86%	5,00E-33	79%
>contig152880 length=155 numreads=6<br>GGATTGATTTCTATATCAATATCATCATCAACTTCTGGTGAAATAAACGCCGATG<br>CAAAT<br>GAAGTGTGTCTTTTACTGTTTGAATCAAAAGGCGACTTTCTTACAAGTCTGTGAA<br>TACCT<br>GTCTCGGTGCGTAACCAACCATAAGCATATTCACC	peptide chain release factor 2 [Francisella sp. TX077308] >ref|WP_013921950.1| peptide chain release factor 2 [Francisella sp. TX077308] >gb|AEI35089.1| Peptide chain release factor 2 [Francisella sp. TX077308]	94.0	94.0	84%	1,00E-21	98%	Francisella tularensis subsp. novicida U112, complete genome	137	77%	3,00E-29	84%
>contig94026 length=223 numreads=10<br>ATAGAGCAAGTGTCAAATATCTAGTATATTGAGTGATTTTCTTTTGACCAGATTC<br>ACCCT<br>CTTTCTTTAACTCAATAAATTTCGGATAAACTGCTGACAGCATTTGAAATATAATT<br>GAGG<br>CAGAAATATATGGCATCACACCAAGGGCAAATATACTCATTTGAGTAAGAGCAC<br>CACCAG<br>AGAACATATTAAACATGCTCATCAAACCACCTGTGCCTGATGA	preprotein translocase subunit SecY [Francisella novicida] >gb|EDN35612.1| preprotein translocase [Francisella novicida GA99-3549]	145	145	85%	1,00E-39	99%	Francisella tularensis subsp. tularensis WY96-3418, complete genome	257	85%	3,00E-65	88%
>contig39042 length=466 numreads=20<br>GTGTTAGAGCATTTATAAATGCTATCGGTTGTATTGCGACTTTTATAGTCTTGCTG<br>ACAA<br>TTATTGAGAGTAAATTTTTTGAAGGTGTTTGGATTGTTATTATAGCTATATCTATA<br>ATCA<br>TGTACTGCTTGTATAAGGTAAAAAAACACTATCTCATAAGAGAACAAAATTTGAC<br>TATTA<br>GTGTTGATGAAGCAATAGTTAGTGCGTCGGTAAATAGAAGGACTAAGCCAAAG<br>ATTGTTC<br>TTTTAGTTTCTCGTATTCATAAAGGAACAATAGAAGCACTACAGTTGGCAAGAAA<br>TTTAT<br>CTGATGATATAACTCCAGTATATGTATCTGCAGATGAGAAAAAGATAGCTAAGA<br>TCAAAG<br>gACAATGGAAAGGGCTAGCTTTTCAGGAAAAGcTACTTATTTtGAGACCAGTTTA<br>TAATT<br>CCTTCATCACTCCTATTGTAAAAATTCTGCATAAAAATGATTTGCG	amino acid transporter [Francisella tularensis subsp. holarctica F92] >ref|WP_015083892.1| amino acid transporter [Francisella tularensis] >gb|AFX71477.1| amino acid transporter [Francisella tularensis subsp. holarctica F92]	233	233	92%	7,00E-71	74%	Francisella tularensis subsp. tularensis WY96-3418, complete genome	207	91%	6,00E-50	75%
>contig82784 length=244 numreads=11<br>TTGCAGAACACTCTATATGCCAACCTGGGCGACCAGCACCCCATGGTGAATCCC<br>AAGCAG<br>GTTCACCTTCTTTAGCCATTTTCCAAAGAACAAAGTCCATAGGATTTTCTTTAATA<br>TCAG<br>ATATTTCTACTCTTGCACCTTGTTGTAGAGCTTCTATATTTtGTTTACTAAGTTTAC<br>CAT<br>AGCCATCAAACTTATCTACACGATAAAAAACATCACCATTGGTAccttGATATGCA<br>AAAC<br>CTTT	cysteinyl-tRNA synthetase [Francisella noatunensis subsp. orientalis str. Toba 04]	161	161	83%	3,00E-45	86%	Francisella cf. novicida 3523, complete genome	251	99%	2E-63	83%
>contig101992 length=210 numreads=7<br>ATCACATGAAAATTACCACTTTCAAGCTTCCTCGTAATTACATTATATATAAGACC<br>TAGG<br>TCAACTATATTTACAGGTATTTCAGGATCATAAACAGTCTTCATCTGATCCCATAC<br>TGCA<br>TCCATATTAATAGGATCACCAGCTTTAATATTATACTCTTCTACTGGGTATTTAAC<br>TACC<br>TGCTTACCTATCGCATCAGCATCTTTACCA	conserved hypothetical protein [Francisella novicida] >gb|EDN36094.1| conserved hypothetical protein [Francisella novicida GA99-3549]	135	135	94%	4,00E-38	84%	Francisella tularensis TIGB03, complete genome	178	87%	2,00E-41	82%

>contig71088 length=275 numreads=10<br>AATGCTTGCACAGCACCTATtATCACACCAATAGAGCTAAAGTTTGCAAAAGAAA<br>CCAAG<br>AAAACTGATATAACGGCTTTGGTATGCTCAGAAATTTCTGTAGAATGCTTTGCTA<br>AATCT<br>TGCATTGCAACAAATTCATTTGTAACAATCTTTGTACCCATTATCTGGCTTGAAAG<br>CATC<br>ACCTCATTACCATGAATATTTAAAACCCATGCCAAAGGATAaaaTACATACCCTAT<br>AATT<br>TGGGTAAACGTAATGCCGATGACATGTGAAAAAAT	Nucleoside permease nupC [Francisella cf. novicida 3523] >ref|WP_014548943.1| nucleoside permease [Francisella novicida] >gb|AEE26966.1| Nucleoside permease nupC [Francisella cf. novicida 3523]	159	159	91%	1,00E-44	80%	Francisella tularensis subsp. holarctica PHIT-FT049, complete genome	165	86%	2,00E-37	78%
>contig195357 length=112 numreads=7<br>ATTATTTATttttAGATTTACCTCCTGGTACGGGTGATATACAACTAACAATTTCAA<br>AAA<br>ATATGCCTGTTACAGGAGCTGTAATTGTAACTACaCcACAAGATTTATCCTT	sodium:proton antiporter [Francisella philomiragia] >gb|EET21499.1| conserved hypothetical protein [Francisella philomiragia subsp. philomiragia ATCC 25015]	75.5	75.5	81%	3,00E-15	97%	Francisella tularensis TIGB03, complete genome	130	79%	3,00E-27	88%
>contig132323 length=174 numreads=8<br>GCTTCATTATGTATTGTTTGGTCTTTCATGTTTTCGGTGTCATAAGCTTGTATATAT<br>GCC<br>TCTGTCATGCAACCGCTTTGcAAaTAATACTTACAAAACTCTAATTgTTtAGATGTT<br>AGT<br>TTAGTAGCCATAGATTAATAATAGAACAAATTTAATACTAGTAATTATATCAGC	phage terminase small subunit [Francisella philomiragia subsp. philomiragia ATCC 25017] >ref|WP_012280385.1| terminase [Francisella philomiragia] >gb|ABZ87210.1| phage terminase, small subunit [Francisella philomiragia subsp. philomiragia ATCC 25017]	91.7	91.7	56%	2,00E-21	95%	Francisella cf. novicida 3523, complete genome	222	73%	1,00E-54	90%
